# Systemic Blockade of ACVR2B Ligands Protects Myocardium from Acute Ischemia-Reperfusion Injury

**DOI:** 10.1016/j.ymthe.2019.01.013

**Published:** 2019-01-24

**Authors:** Johanna Magga, Laura Vainio, Teemu Kilpiö, Juha J. Hulmi, Saija Taponen, Ruizhu Lin, Markus Räsänen, Zoltán Szabó, Erhe Gao, Lea Rahtu-Korpela, Tarja Alakoski, Johanna Ulvila, Mika Laitinen, Arja Pasternack, Walter J. Koch, Kari Alitalo, Riikka Kivelä, Olli Ritvos, Risto Kerkelä

**Affiliations:** 1 Research Unit of Biomedicine, Department of Pharmacology and Toxicology, University of Oulu, 90220 Oulu, Finland; 2 Biocenter Oulu, University of Oulu, 90220 Oulu, Finland; 3 Neuromuscular Research Center, Biology of Physical Activity, Faculty of Sport and Health Sciences, University of Jyväskylä, 40014 Jyväskylä, Finland; 4 Department of Physiology, Faculty of Medicine, University of Helsinki, 00290 Helsinki, Finland; 5 Medical Research Center Oulu, Oulu University Hospital and University of Oulu, 90220 Oulu, Finland; 6 Wihuri Research Institute and Translational Cancer Biology Program, Faculty of Medicine, University of Helsinki, 00290 Helsinki, Finland; 7 Center for Translational Medicine, Temple University School of Medicine, Philadelphia, PA 19140, USA; 8 Department of Medicine, University of Helsinki, 00029 Helsinki, Finland; 9 Department of Medicine, Helsinki University Hospital, 00029 Helsinki, Finland

**Keywords:** activins, ACVR2B, growth differentiation factors, ischemia-reperfusion injury

## Abstract

Activin A and myostatin, members of the transforming growth factor (TGF)-β superfamily of secreted factors, are potent negative regulators of muscle growth, but their contribution to myocardial ischemia-reperfusion (IR) injury is not known. The aim of this study was to investigate if activin 2B (ACVR2B) receptor ligands contribute to myocardial IR injury. Mice were treated with soluble ACVR2B decoy receptor (ACVR2B-Fc) and subjected to myocardial ischemia followed by reperfusion for 6 or 24 h. Systemic blockade of ACVR2B ligands by ACVR2B-Fc was protective against cardiac IR injury, as evidenced by reduced infarcted area, apoptosis, and autophagy and better preserved LV systolic function following IR. ACVR2B-Fc modified cardiac metabolism, LV mitochondrial respiration, as well as cardiac phenotype toward physiological hypertrophy. Similar to its protective role in IR injury *in vivo*, ACVR2B-Fc antagonized SMAD2 signaling and cell death in cardiomyocytes that were subjected to hypoxic stress. ACVR2B ligand myostatin was found to exacerbate hypoxic stress. In addition to acute cardioprotection in ischemia, ACVR2B-Fc provided beneficial effects on cardiac function in prolonged cardiac stress in cardiotoxicity model. By blocking myostatin, ACVR2B-Fc potentially reduces cardiomyocyte death and modifies cardiomyocyte metabolism for hypoxic conditions to protect the heart from IR injury.

## Graphical Abstract

**Figure:**
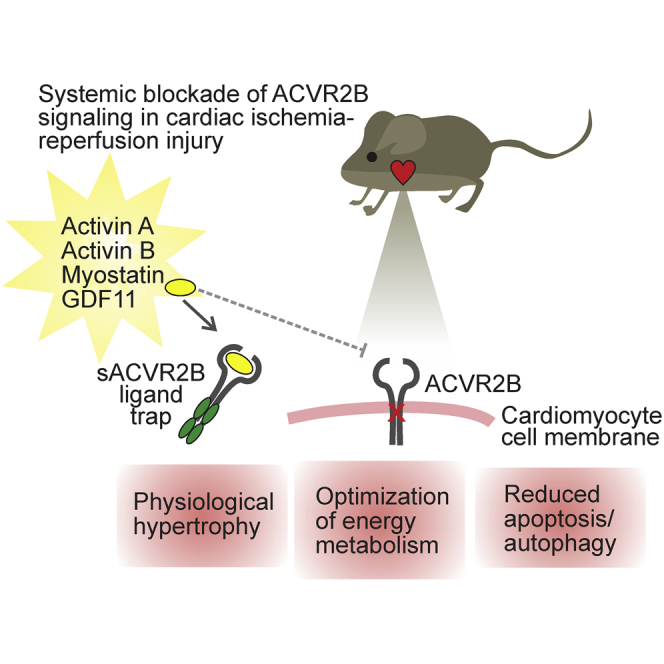

## Teaser

Magga et al. report that systemic blockade of activin receptor type 2 (ACVR2B) ligands protects from cardiac ischemia-reperfusion injury reducing infarct size, apoptosis, and autophagy. Mechanistically, systemic blockade of ACVR2B ligands reduced SMAD2 activation and modified cardiomyocyte metabolism for hypoxic conditions.

## Introduction

Development of the heart is guided by secreted morphogens including members of the transforming growth factor (TGF)-β superfamily.^1^ In addition to their regulatory function in organogenesis, the TGF-β family of growth factors, including activins, bone morphogenetic proteins (BMPs), and growth differentiation factors (GDF), are known to regulate cardiac physiology and pathophysiology in the adult heart. These factors signal through type I and type II receptors, both of which are transmembrane serine and threonine kinases. Activins and GDFs bind to activin receptor IIA and B (ACVR2A and ACVR2B),^2^, ^3^ which in turn activate type I receptors such as activin receptor-like kinases (ALK) ALK4 and ALK5, activating downstream molecule SMAD2/3.^4^, ^5^ SMADSs regulate a number of myogenic genes, such as myoD, myogenin, and Myf5, that are involved in cellular hypertrophy, proliferation, or differentiation.^6^ In addition to signaling via SMAD proteins, GDFs also signal through noncanonical pathways to regulate cardiomyocyte growth^7^ by upregulation of atrophy-related atrogenes or autophagy genes, resulting in proteasome-dependent muscle protein degradation. Noncanonical ACVR2B pathways have also been shown to regulate MAP kinases.^5^

Activin A is upregulated in the heart after myocardial infarction (MI) or ischemia-reperfusion (IR) injury.^8^, ^9^ Serum levels of activin A increase in MI, and its expression levels correlate with creatinine kinase, as a measure of infarct size.^10^ In an experimental model, cardiac myostatin (also known as GDF8) is upregulated immediately after MI.^11^ Follistatin, an endogenous antagonist to myostatin and activin, has been shown to reduce IR injury in mice.^9^ When utilizing myostatin-deficient mice, it was recently shown that the absence of myostatin improves cardiac function after MI.^12^ In contrast to reports suggesting activin A as a culprit in IR injury, overexpression of activin A has also been shown to be protective against cardiomyocyte death, and its antagonism by Fstl3 exacerbates IR injury.^8^ Myocardial stretch was recently shown to induce activin A in a genome-wide time series study of gene expression changes in stretched neonatal cardiomyocytes.^13^ Interestingly, activin A antagonists follistatin and Fstl3 were briefly upregulated after induction of stretch and substantially before activation of activin A.^13^ It is not understood how activins and GDFs contribute to cardiomyocyte viability and function in myocardial ischemia.

Reperfusion by percutaneous catheter angioplasty is the main treatment for MI. While necessary to salvage ischemic myocardium, reperfusion itself may impair microvascular function and paradoxically trigger further injury.^14^ Cardioprotection from reperfusion injury has been experimentally studied by local ischemic pre- and post-conditioning procedures as well as by remote ischemic preconditioning. This, achieved by short repetitive IR periods, is thought to activate pathways targeting mitochondria and thus reduce the formation of reactive oxygen species as well as inhibit the opening of mitochondrial permeability transition pore (mPTP).^14^

In addition, various growth factors and pharmacological agents have been explored for reducing reperfusion injury. Atrial natriuretic peptide (ANP), glucose-insulin-potassium therapy, glucagon like peptide-1 (GLP-1) analog exenatide or β-blocker metoprolol can potentially induce cardioprotection by inducing cGMP/PKG signaling, promoting glucose metabolism and utilization or reducing myocardial oxygen consumption, respectively.^15^ Furthermore, agents targeting mitochondria by enhancing mitochondrial energetics or inhibiting mPTP opening have shown efficacy in experimental models of IR injury. However, many of these have not (yet) been shown to be cardioprotective in clinical studies.^16^ Some of them have failed in clinical studies, possibly due to timing or dosing of the agent or heterologous patient population selected for the study.

Despite current invasive strategies to treat MI, novel cardioprotective agents are still needed to attenuate IR injury in order to prevent ischemia-induced heart failure and improve prognosis. In this study, we show that treating mice with ACVR2B-Fc decoy receptor protects the myocardium from IR injury. In earlier characterization, ACVR2B-Fc has been shown to bind endogenous ligands myostatin (GDF8), GDF11, and activin A with high affinity^17^ and inhibit physiological responses of ACVR2B ligands.^18^, ^19^, ^20^ Our results indicate that ACVR2B ligand myostatin activates SMAD2/3 and contributes to IR injury. This is salvageable by pharmacological inhibitor ACVR2B-Fc.

## Results

### Systemic Blockade of ACVR2B Ligands Reduces Ischemic Injury and Restores Cardiac Function in an Experimental Model of Ischemia Reperfusion

**Figure 1:**
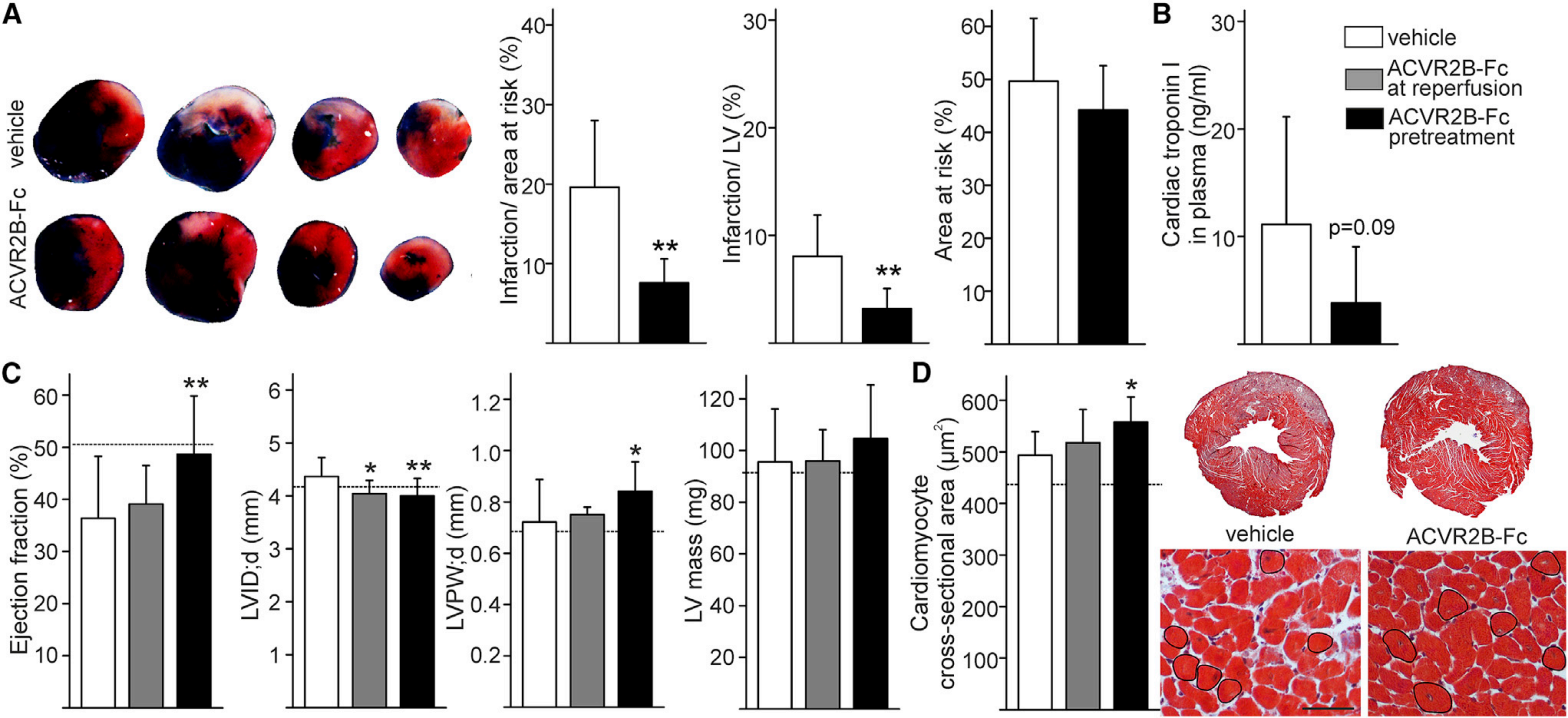
ACVR2B-Fc Reduces Ischemia-Reperfusion Injury and Restores Cardiac Function Mice were treated with vehicle (white columns) or with a soluble decoy receptor ACVR2B-Fc at reperfusion (ACVR2B-Fc at reperfusion, gray columns) or 24 h prior to/at reperfusion (ACVR2B-Fc pretreatment, black columns). IR was achieved by transient ligation of left anterior descending (LAD) coronary artery for 30 min, followed by reperfusion for 24 h. (A) ACVR2B-Fc reduced infarcted area as determined with triphenyltetrazolium chloride (TTC stain). n = 7, 8. (B) ACVR2B-Fc reduced cardiac troponin I release into plasma (p = 0.09). n = 8, 9. (C) As determined with echocardiography, ACVR2B-Fc preserved cardiac function after IR observed as preserved ejection fraction. ACVR2B-Fc also reduced left ventricular diameter in diastole (LVID;d) and increased LV posterior wall thickness (LVPW;d) in IR mice with slightly increased total LV mass. Sham values are shown as a dotted line. n = 19, 9, 18. Full echo data in Table S2. (D) ACVR2B-Fc increased cardiomyocyte cross-sectional area. Masson trichrome stained heart section cut horizontally (above) and representative pictures of single cross-sectional cardiomyocytes circled for cardiomyocyte area analysis (below). Sham values are shown as a dotted line. Scale bar, 50 μm. n = 10, 9, 8. Data are presented as mean ± SD. *p < 0.05, **p < 0.01.

To study the contribution of ACVR2B signaling to ischemic myocardial injury, we treated mice with a soluble decoy receptor of ACVR2B 24 h before ischemia (“ACVR2B-Fc pretreatment”) to block the function of ACVR2B ligands and subjected the mice to transient IR by ligation of LAD. 30 min of ischemia followed by 6 h or 24 h of reperfusion resulted in left ventricular (LV) cell death and deteriorated cardiac function 24 h after IR. When analyzed with triphenyltetrazolium chloride (TTC) stain, ACVR2B-Fc reduced infarcted area in LV (p < 0.01; Figure 1A). This was accompanied by reduced, although not statistically significant, release of cardiac troponin I into plasma (p = 0.09; Figure 1B). Treatment with ACVR2B-Fc preserved cardiac function after IR as measured by echocardiography, but not if the treatment was initiated at reperfusion (“ACVR2B-Fc at reperfusion”). Ejection fraction was significantly better preserved with ACVR2B-Fc (p < 0.01; Figure 1C; see also Figure S1). In addition, fractional shortening and endocardial fractional area change were improved after ACVR2B-Fc treatment in comparison to vehicle-treated IR mice (p < 0.01; Table S2). Administration of ACVR2B-Fc reduced LV diameter (p < 0.01) and increased LV posterior wall thickness in IR mice (p < 0.05, Figures 1C and 1D; see also Table S2). This was associated with increased cardiomyocyte cross-sectional area (p < 0.05; Figure 1D), slightly increased LV mass (Figure 1C), and total heart weight (146 ± 18 mg in vehicle-treated and 158 ± 19 mg in ACVR2B-Fc-treated IR mice). A similar effect on cardiac hypertrophy was detected in healthy mice treated with sACVR2B as cardiomyocyte cross-sectional area was increased (Figure S2). The same mice also showed a non-significant increase in skeletal muscle hypertrophy (Figure S2). Cardiac hypertrophy was accompanied by transient phosphorylation and inactivation of GSK3β (Figure S2), which promotes LV hypertrophy and enhances resistance of cardiomyocytes to oxidative stress.^21^

### Systemic Blockade of ACVR2B Ligands Reduces Ischemic Injury and Suppresses SMAD2, Apoptotic Stress-Induced Signaling Pathways, and Autophagy

**Figure 2:**
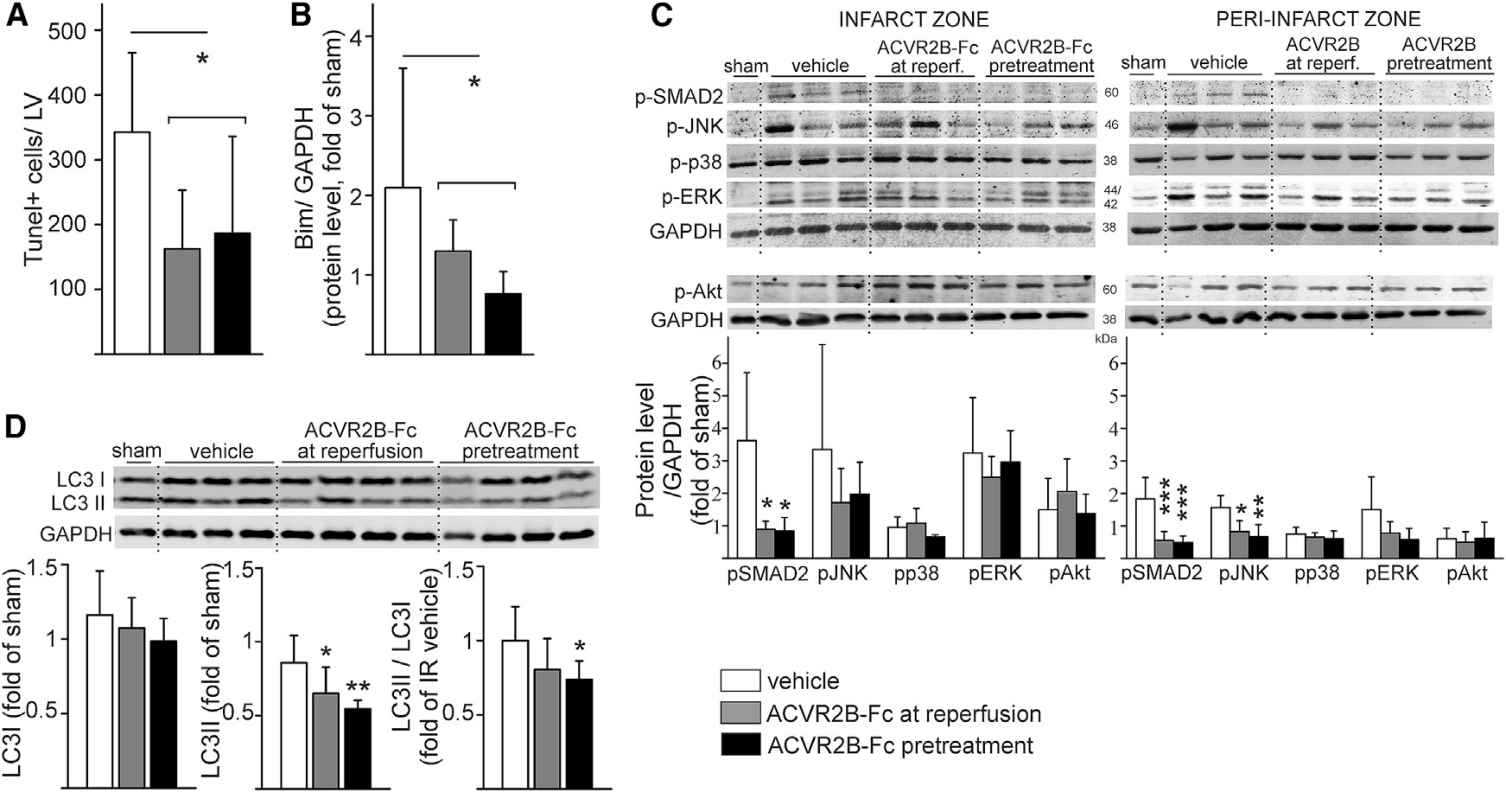
ACVR2B-Fc Reduces Activation of SMAD2, Apoptotic Stress-Induced Signaling Pathways, and Autophagy Mice were treated with vehicle (white columns), with soluble decoy receptor ACVR2B-Fc only at reperfusion (gray columns), or with pretreatment of ACVR2B-Fc 24 h prior to/at reperfusion (black columns). IR was achieved by transient ligation of left anterior descending (LAD) coronary artery for 30 min, followed by reperfusion for 6 h. (A) ACVR2B-Fc reduced apoptosis. n = 5, 6, 6. (B) ACVR2B-Fc reduced expression of Bcl-2 family pro-apoptotic protein Bim. n = 5, 6, 6. (C) ACVR2B-Fc inhibited IR-induced activation of SMAD2 protein in infarcted and peri-infarcted zone. ACVR2B-Fc also reduced IR-induced phosphorylation of JNK but had no effect on activation of p38, ERK1/2, or Akt. n = 5, 6, 6. (D) ACVR2B-Fc reduced autophagosomal lipidated LC3II form and decreased autophagosomal/cytoplasmic/LC3II/LC3I ratio in LV as analyzed 24 h after reperfusion. n = 7, 8, 8. Data are presented as mean ± SD. *p < 0.05, **p < 0.01, ***p < 0.001.

To study the mechanisms of ACVR2B-Fc-mediated protection, we performed further analysis for apoptotic pathways. A TUNEL stain was performed 6 h after reperfusion and showed that ACVR2B-Fc reduced apoptosis in LV (p < 0.05; Figure 2A; Figure S2). This was accompanied by reduced expression of Bcl-2 family pro-apoptotic protein Bim (p < 0.05; Figure 2B). IR-induced activation of SMAD2 protein in the infarcted and peri-infarcted zone was inhibited by ACVR2B-Fc (p < 0.001; Figure 2C). In addition, ACVR2B-Fc reduced IR-induced phosphorylation of JNK (p < 0.01; Figure 2C) while having no effect on activation of ERK1/2 or p38. Furthermore, ACVR2B-Fc did not affect activation of phosphatidylinositol 3-kinase (PI3K)/Akt pathway (Figure 2C). ACVR2B signaling is known to promote autophagy, leading to protein degradation. Systemic blockade of ACVR2B ligands by ACVR2B-Fc led to reduction of autophagosomal LC3II form and decreased autophagosomal/cytoplasmic/LC3II/LC3I ratio (p < 0.05; Figure 2D) indicating reduced autophagy in the heart.

Since ACVR2B ligand GDF11 negatively regulates erythrocyte maturation^7^ and activin A associates with inflammatory processes,^22^ we additionally studied whether erythropoiesis, leukocyte infiltration, or inflammatory response after IR are affected by ACVR2B-Fc. Blood cell count performed 24 h after IR did not reveal any difference in red blood cell count between ACVR2B-Fc and vehicle-treated mice (Table S3), and leukocyte counts were also not affected. We then determined if ACVR2B-Fc affects granulocyte infiltration after IR. As analyzed from neutrophil stain, ACVR2B-Fc did not affect neutrophil infiltration into LV (Figure S3). In addition, ACVR2B-Fc did not significantly reduce the expression of pro-inflammatory cytokines or chemokines in LV (Figure S3). Consequently, cardioprotection by ACVR2B-Fc was not explained by enhanced erythropoiesis or reduced inflammatory response.

### ACVR2B-Fc Inhibits Myostatin-Mediated Activation of SMAD2/3 Pathway in Cardiomyocytes

**Figure 3:**
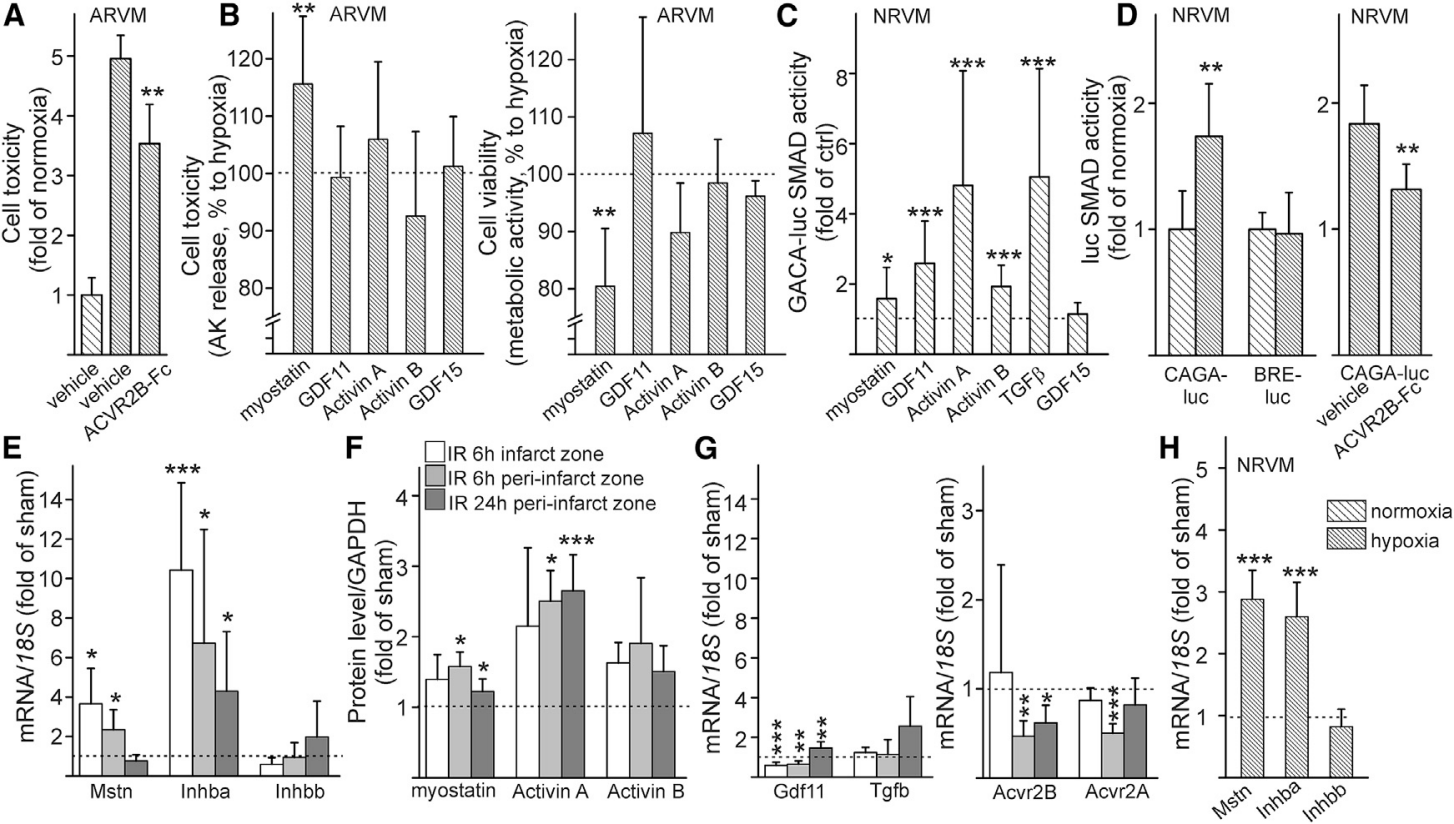
ACVR2B-Fc Protects Cardiomyocytes in Hypoxic Conditions and Inhibits Myostatin-Activated SMAD2/3 Pathway in Cardiomyocytes (A) ACVR2B-Fc protects adult rat ventricular cardiomyocytes (ARVM) from hypoxia-induced cell death as measured with adenylate kinase release. n = 6. (B) ACVR2B ligand myostatin reduces cell survival in hypoxia. Cell toxicity was assessed with adenylate kinase release, and cell viability was determined with resazurin assay measuring cell metabolism. The hypoxia value is shown as a dotted line. n = 6. (C) Neonatal rat ventricular cardiomyocytes (NRVM) were transfected with CAGA-luc SMAD2/3 reporter, and subsequently luciferase reporter activity was analyzed after treatment with ACVR2B ligands, TGF-β, or the GFRAL receptor ligand GDF15. n = 12. (D) Hypoxia induced SMAD2/3-dependent promoter activity in NRVM while SMAD1/5/8 signaling was not activated. ACVR2B-Fc reduced hypoxia-induced SMAD2/3-dependent promoter activity in NRVM. n = 6. (E) Expression of ACVR2B ligands myostatin (*Mstn*), activin A (*Inhba*), and activin B (*Inhbb*) in infarct and peri-infarct zone in LV 6 and 24 h after IR, analyzed by qPCR. The sham value is shown as a dotted line. n = 5–6 (6 h IR); n = 8–9 (24 h IR). (F) The levels of ACVR2B ligands in LV, analyzed by western blotting. n = 5 (6 h IR); n = 9 (24 h IR). (G) Expression of ACVR2B ligand *Gdf11* and *Tgfb*, as a control, and ACVR2B receptors *Acvr2B* and *Acvr2A* in LV 6 and 24 h after IR, analyzed by qPCR. n = 5–6 (6 h IR); n = 8–9 (24 h IR). (H) As confirmed in NRVM, myostatin and activin A were upregulated after hypoxia. The normoxia value is shown as dotted line. n = 6. Data are presented as mean ± SD. *p < 0.05, **p < 0.01, ***p < 0.001.

First, we investigated whether ACVR2B-Fc induces cardioprotection directly to cardiomyocytes during hypoxia. Similar to cardioprotection *in vivo*, ACVR2B-Fc provided protection to adult cardiomyocytes from hypoxia-induced cell death (p < 0.01; Figure 3A). We then wanted to decipher which ACVR2B ligands could contribute to ischemic injury in adult cardiomyocytes. Myostatin and, to smaller extent, activin A, exacerbated ischemic injury in adult cardiomyocytes, while GDF11, activin B, or GDF15 had no effect (Figure 3B).

To study the ACVR2B-Fc-mediated SMAD signaling in cellular level, we transfected neonatal cardiomyocytes with CAGA-luc SMAD2/3 reporter or BRE-luc SMAD1/5/8 reporter and performed luciferase promoter assay to detect respective SMAD activity. To validate the model, we stimulated neonatal cardiomyocytes with factors expected to activate SMADs and to confirm that this signaling occurs in cardiomyocytes. As expected, myostatin, GDF11, activin A, activin B, and TGF-β induced SMAD2/3-dependent promoter activity (Figure 3C). GDF15, which signals via GFRAL receptor (not via ACVR2B receptor), was used here as a negative control and did not induce SMAD2/3 activity (Figure 3C). None of these ligands stimulated BRE-luc, which was used to assess SMAD1/5/8 activity, and which was activated by BMP4 (Figure S4). To confirm the efficacy of ACVR2B-Fc in reduction of SMAD activation, primary neonatal cardiomyocytes were subjected to hypoxia. As seen in Figure 3D, hypoxia induced SMAD2/3-dependent promoter activity in neonatal cardiomyocytes, while SMAD1/5/8 signaling was not activated. Administration of ACVR2B-Fc, which reduced SMAD2 signaling *in vivo*, reduced hypoxia-induced SMAD2/3-dependent promoter activity (p < 0.01; Figure 3D).

We then analyzed the time course of expression of ACVR2B ligands in the heart 6 h and 24 h after IR *in vivo*. qPCR analysis showed that levels of myostatin (*Mstn*) and activin A (*Inhba*) were upregulated in the infarct and/or peri-infarct zones while activin B (*Inhbb*) was not affected (Figure 3E; Figure S4). This was accompanied by corresponding changes in protein levels measured from LV by western blotting (Figure 3F). Similarly, immunostaining of LV sections showed increased activin A levels in the infarct and peri-infarct zones (Figure S4), localization of activin A in cardiomyocytes and, to a smaller extent, in endothelial cells. Interestingly, activin B was mainly detected in the infarct and peri-infarct zones in leukocytes, apparently neutrophils, and was only found in cardiomyocytes 24 h after IR and to a smaller extent than observed in the leukocytes. Myostatin levels were already high at basal levels in sham hearts, and there was a minor increase in myostatin levels in infarct and peri-infarct zones at 6 h and 24 h after IR; myostatin expression was only localized to cardiomyocytes (Figure S4).

Different from other ACVR2B ligands, *Gdf11* was downregulated in the early phase after IR but upregulated at 24 h (Figure 3G). No change was observed in *Tgfb* expression following IR (Figure 3G). When determining the expression levels of activin receptors, both *Acvr2B* and *Acvr2A* receptors were downregulated in the peri-infarct zone at 6 h after IR (Figure 3G). After 24 h, *Acvr2B* downregulation sustained, while *Acvr2A* expression was elevated back to basal level. Expression of *Bmpr2*, the receptor for BMP ligands, was not affected (1 ± 0.17 in sham versus 0.93 ± 0.29 at IR 24 h). Finally, as confirmed in neonatal cardiomyocytes, myostatin and activin A were upregulated after hypoxia, while the expression of activin B was not changed (Figure 3H). In summary, our data suggest myostatin and activin A are elevated shortly after hypoxia and they activate ACVR2B in cardiomyocytes, leading to increased SMAD2/3 activity. Myostatin exacerbated hypoxic injury, which was associated with increased cardiomyocyte death. Similar to cardioprotective effects of ACVR2B-Fc observed in IR model *in vivo*, ACVR2B-Fc directly protected cardiomyocytes *in vitro*.

### Systemic Blockade of ACVR2B Ligands Optimizes Cardiac Metabolism to Hypoxic Conditions in the IR Model

**Figure 4:**
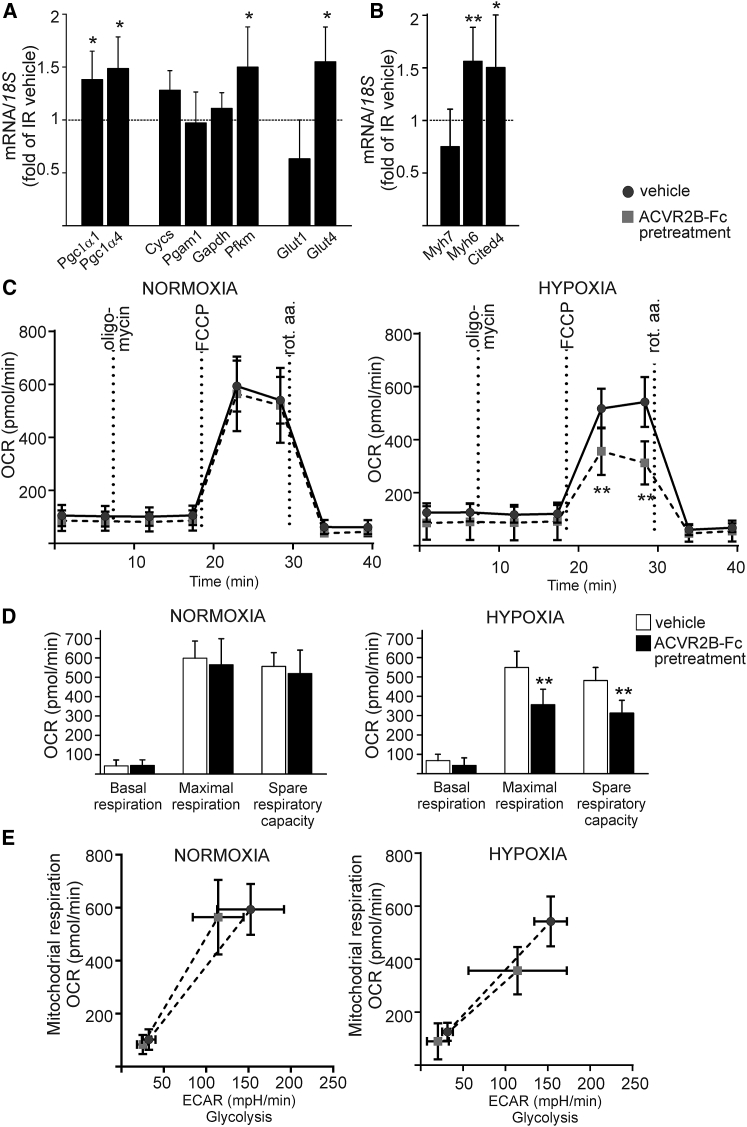
ACVR2B-Fc Optimizes Metabolism to Hypoxic Conditions in IR Expression of genes were analyzed with qPCR 24 h after IR from the peri-infarct zone. (A) ACVR2B-Fc upregulated expression of peroxisome proliferator-activated receptor gamma coactivator 1 isoforms PGC1α1 and PGC1α4 and did not affect the gene expression of oxidative phosphorylation enzyme cytochrome C (*Cycs*), but it increased expression of glycolytic phosphofructokinase enzyme *Pfkm* and insulin-regulated glucose uptake transporter *Glut4*. Vehicle-treated IR mouse values are shown as dotted line. n = 9, 8. (B) ACVR2B-Fc upregulated expression of MHC-α (*Myh6*) fast twitch myosin fibers and *Cited4*, a transcription factor involved in physiological hypertrophy. Vehicle-treated IR mouse values are shown as a dotted line. n = 9, 8. Data are presented as mean ± SD. *p < 0.05, **p < 0.01. (C) ACVR2B-Fc reduced metabolism in adult mouse cardiomyocytes after 4 h hypoxia followed by 1 h reperfusion as determined by oxygen consumption rate (OCR) with bioenergetic mito stress assay. (D) The values for basal and maximal respiration and spare respiratory capacity were calculated from OCR graphs. n = 13, 11 (normoxia); n = 6, 5 (hypoxia). (E) Bioenergetic phenotype profile visualized from mito stress assay data. Basal phenotype from the beginning of the experiment versus stressed phenotype (after oligomycin and FCCP injections) in cells subjected to normoxia or hypoxia. n = 13, 11 (normoxia); n = 6, 5 (hypoxia).

We next studied the effect of ACVR2B-Fc on cardiac metabolism following cardiac IR injury. We found that administration of ACVR2B-Fc upregulated the expression of peroxisome proliferator-activated receptor gamma coactivator 1 (*Ppargc1a*) isoforms Pgc1α1 and Pgc1α4 (p < 0.05; Figure 4A), which are central regulators of mitochondrial energy production. ACVR2B-Fc did not affect the gene expression of oxidative phosphorylation enzyme cytochrome C (*Cycs*) or glycolytic enzymes *Pgam1* or *Gapdh* (Figure 4A). However, improvement of energy metabolism by ACVR2B-Fc was associated with an increased expression of glycolytic phosphofructokinase enzyme *Pfkm* and upregulation of insulin-regulated glucose transporter *Glut4* (p < 0.05; Figure 4A), suggesting an increased glucose uptake and glycolysis. ACVR2B-Fc increased phosphorylation of acetyl-CoA carboxylase, reducing its enzymatic activity in the fatty acid synthesis pathway in healthy hearts (Figure S4). However, ACVR2B-Fc did not reduce fatty acid synthesis in IR hearts (Figure S4).

Administration of ACVR2B-Fc had no effect on acute IR-injury-induced increase in expression of atrial or B-type natriuretic peptides ANP (*Nppa*) (IR vehicle 3.2 ± 1.4 versus IR ACVR2B-Fc pretreatment 2.6 ± 1.1, p = 0.52) or BNP (*Nppb*) (IR vehicle 1.9 ± 0.8 versus IR ACVR2B-Fc pretreatment 1.5 ± 0.6, p = 0.44) but affected the composition of cardiomyocyte myosin fibers. ACVR2B-Fc slightly, although not significantly, decreased expression of myosin heavy chain (MHC)-β (*Myh7*) slow twitch isoform while increasing expression of MHC-α (*Myh6*) fast twitch myosin fibers (p < 0.01; Figure 4B). This was accompanied with increased expression of *Cited4* (p < 0.05; Figure 4B), a transcription factor involved in physiological hypertrophy.^23^, ^24^

To confirm the effect of ACVR2B-Fc on cardiomyocyte metabolism, we performed a bioenergetic assay in cardiomyocytes *in vitro*. Adult LV cardiomyocytes obtained from mice treated with ACVR2B-Fc for 48 h showed reduced metabolic activity with attenuated oxygen consumption when subjected to hypoxic conditions *in vitro* (Figure 4C). Cardiomyocytes of ACVR2B-Fc-treated mice showed both reduced maximal respiration and reduced spare respiratory capacity compared to cardiomyocytes from vehicle-treated mice (Figure 4D). We did not detect pronounced induction of glycolysis (Figure 4E), and upregulation of mitochondrial glycolytic enzymes detected by qPCR may thus represent a compensatory increase of metabolic enzymes after myocardial hibernation.

### Systemic Blockade of ACVR2B Ligands during Prolonged Cardiac Stress Improves LV Function

**Figure 5:**
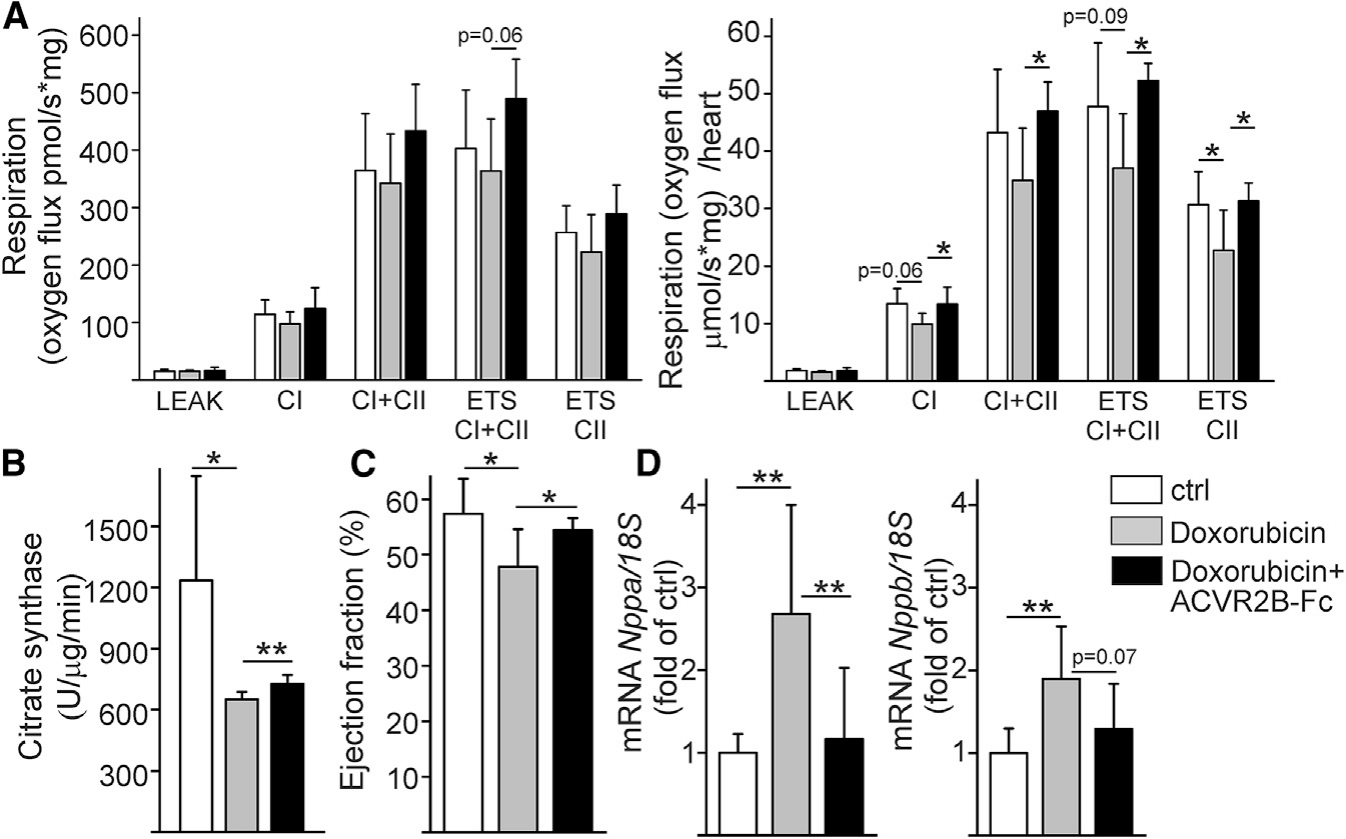
ACVR2B-Fc Improves LV Function under Prolonged Cardiac Stress (A) Mice were treated with anthracycline doxorubicin for 2-week period, which induces cardiotoxicity. Mice were treated simultaneously with vehicle or ACVR2B-Fc for 2 weeks and LV tissue was analyzed with high-resolution respirometer. ACVR2B-Fc improved cardiac respiration in doxorubin-stressed hearts. Right panel represents respiration capacity for the whole heart. CI, complex I; CII, complex II; ETS, electron transfer system (maximal uncoupled respiration). n = 7. (B) ACVR2B-Fc also slightly improved citrate synthase activity. n = 7. (C) As analyzed with echocardiography, ACVR2B-Fc preserved cardiac systolic function in cumulative doxorubicin-induced toxicity at 4 weeks. n = 9, 6, 9. Full echo data in Table S4. (D) As analyzed with qPCR from LV at 4 weeks, ACVR2B-Fc reduced doxorubicin-induced upregulation of natriuretic peptides ANP (*Nppa*) and BNP (*Nppb*). n = 9, 7, 8. Data are presented as mean ± SD. *p < 0.05, **p < 0.01.

To determine the long-term effects of ACVR2B-Fc-induced metabolic changes on cardiac function, we measured mitochondrial respiration in LV *ex vivo*. We treated mice with anthracycline antitumor agent doxorubicin, which induces cardiotoxicity by increased oxidative stress, alterations in ion homeostasis, inhibition of protein synthesis, and eventually, mitochondrial failure.^25^ To study if ACVR2B-Fc could protect the heart from doxorubicin-induced deterioration of cardiac metabolic function, we measured oxidative function from LV utilizing a high-resolution respirometer. We found that ACVR2B-Fc treatment improved cardiac respiration in doxorubin-stressed hearts (Figure 5A). ACVR2B-Fc also slightly, but significantly, improved citrate synthase activity (Figure 5B). This result may be independent of mitochondrial number, as doxorubicin or ACVR2B-Fc had no significant effect on the mitochondrial respiratory chain (OXPHOS and cytochrome C) protein contents (data not shown). Furthermore, utilizing echocardiography, we assessed the effect of ACVR2B-Fc treatment on cardiac function after cumulative doxorubicin-induced toxicity. We found that treatment of mice with ACVR2B-Fc inhibited the doxorubicin-induced deterioration of cardiac systolic function (Figure 5C; see also Table S4). qPCR analysis of cardiac samples showed that administration of ACVR2B-Fc reduced doxorubicin-induced upregulation of natriuretic peptides ANP (*Nppa*) and BNP (*Nppb*) (Figure 5D), indicators of pathological cardiac remodeling.^26^

## Discussion

### ACVR2B-Fc Contributes to Ischemia Protection by Regulation of Myostatin Signaling in Cardiomyocytes

Repetitive ischemic preconditioning downregulates expression of myostatin in both ischemic and remote myocardium. This suggests downregulation of myostatin is associated with preconditioning-induced cardioprotection in reperfusion injury.^27^ Here, we demonstrate that systemic blockade of ACVR2B ligands protects the heart from IR injury by reducing hypoxia-activated myostatin/SMAD2 signaling. Treatment with ACVR2B-Fc reduces apoptosis and optimizes cardiomyocyte energy metabolism in hypoxic conditions.

In addition to BMPs,^28^, ^29^ myostatin and activin A are potent regulators of muscle growth.^30^ Myostatin is predominantly expressed in the skeletal muscle but also to lower extent in the heart. Myostatin is an important negative regulator of muscle growth, as evidenced by massive skeletal muscle hypertrophy in myostatin knockout mice.^31^ Constitutive myostatin knockout induces myofiber hypertrophy and hyperplasia during development and^32^ results in downregulation of genes encoding slow isoforms of contractile proteins and genes encoding proteins involved in energy metabolism.^33^ We also detected expression of these genes modified in our ACVR2B-Fc-treated IR hearts. In contrast, earlier studies with post-developmental reduction of myostatin expression by Cre-lox recombination or by ACVR2B-Fc resulted in hypertrophy, but not to the downregulation of genes encoding slow isoforms of skeletal muscle contractile proteins or genes encoding proteins involved in energy metabolism.^34^, ^35^

Activins mainly reside in gonadal tissue and regulate reproduction by stimulating FSH release from the pituitary gland. In addition, activin A is expressed in the skeletal muscle and the heart and regulates essential biological functions, such as cell proliferation and differentiation, immune response, and angiogenesis.^5^ Similarly to myostatin, activin A negatively and prominently regulates muscle growth.^9^, ^30^ In a recent study in zebrafish, activin A and myostatin were shown to have opposite effects on cardiac repair after cryoinjury: activin A led to accelerated recovery, whereas myostatin hindered the regeneration process. Furthermore, myostatin was suggested to bind predominantly to ACVR2B and activate SMAD2, while activin A was suggested to bind to ACVR2A and promote activation of SMAD3.^36^ This is in line with our findings showing that myostatin exacerbates ischemic injury and ACVR2B-Fc blocks the activation of myostatin-activated SMAD2. However, our data does not allow us to determine to which extent the ACVR2B-Fc-mediated cardioprotection stems from inhibition of canonical SMAD2 pathway or inhibition of non-canonical JNK pathway. SMAD2/3 controls genes regulating hypertrophy and atrophy, even apoptosis. JNK can also regulate hypertrophy, metabolism, and mitochondria-mediated cell death in response to oxidative stress.^37^ JNK can even interact with SMAD2 pathway in regulation of muscle remodeling.^38^ Apparently, the anti-apoptotic effect of ACVR2B-Fc is mediated by inhibition of JNK activity but occurs in cross-talk with other signaling pathways, including SMAD2.

BMPs have been shown to contribute to ischemic injury,^39^ but we did not detect immediate BMP activation in cardiomyocytes in response to hypoxia by measuring BRE-luc promoter activity. Fstl1, inhibiting BMPs and their signaling via the SMAD1/5/8 pathway, has been shown to reduce IR injury by reduction of apoptosis and inflammatory response.^40^, ^41^ In a recent study, Fstl1 was also shown to alter energy substrate metabolism and increase oxidative respiration in the heart.^42^ It remains to be shown how BMP and GDF pathways, via SMAD1/5/8 and SMAD2/3, respectively, or via noncanonical pathways, overlap in their contribution to IR injury.

GDF11, although a close homolog to myostatin, has versatile effects partly divergent from myostatin. Myostatin predominantly affects muscle mass, while the ablation of GDF11 results in defects in skeletal patterning during embryogenesis, resulting in perinatal lethality.^43^, ^44^ Unlike myostatin, GDF11 also participates in erythropoiesis in adult. GDF11 was formerly considered as a rejuvenation factor, and restoration of levels of GDF11 were shown to provide protection from age-related pathological cardiac hypertrophy.^45^ In subsequent studies, aging was not associated with a decrease in circulating GDF11 levels,^46^ and elevation of GDF11 did not provide therapeutic effect for cardiac hypertrophy.^47^ In skeletal muscle, GDF11 expression was even shown to increase during aging and inhibit muscle regeneration, similar to inhibitory effects of myostatin.^48^ Inducing supraphysiological levels of GDF11 led to both skeletal and cardiac muscle atrophy, while myostatin reduced only skeletal muscle growth.^49^ We detected upregulation of cardiac myostatin and activin A expression immediately after ischemia, while expression of GDF11 was upregulated at a later phase. This is of interest since GDF11 was recently shown to reduce cardiac remodeling after IR injury.^50^

Our results thus confirm that myostatin and its close homolog GDF11 are differently expressed in the heart in response to ischemia and indicate that they may participate in different processes. Furthermore, GDF11 did not affect cardiomyocyte survival in acute hypoxia, while administration of myostatin exacerbated cardiomyocyte death. This suggests that, blocking the effects of GDF11 as a bystander, ACVR2B-Fc does not exacerbate myocardial ischemic injury. We cannot fully rule out that additionally, blockade of GDF11 could partly contribute to benefits of ACVR2B-Fc in IR. GDF11 is known to negatively affect erythrocyte maturation,^7^ and its blockade by ACVR2B-Fc increases extramedullary hematopoiesis leading to splenomegaly.^30^ When performing erythrocyte count 24 h post-MI, we found short-term treatment with ACVR2B-Fc did not increase the number of erythrocytes, excluding increased red blood cell availability as a possible benefit during IR.

### ACVR2B-Fc Contributes to Ischemia Protection by Regulation of Catabolic Pathways and Hypertrophy

In the present study, administration of ACVR2B-Fc rapidly induced cardiomyocyte growth. This was not accompanied by downregulation of atrogenes or induction of Akt, although GSK3β phosphorylation was transiently increased. In a previous study using our ACVR2B-Fc, blocking ACVR2B ligands did not increase protein synthesis in the healthy heart as it does in the skeletal muscle.^20^ Here, administration of ACVR2B-Fc reduced lipidated LC3, a marker of autophagy, in the heart, suggesting that decreased autophagy may be a mechanism of increased cardiomyocyte size by ACVR2B-Fc. The administration of ACVR2B-Fc has also reduced LC3 lipidation in skeletal muscle,^19^ which probably occurs through blocking of myostatin, as myostatin can induce autophagy.^51^ It is also possible that ACVR2B-Fc-induced higher glucose concentration or uptake contributed to increased hypertrophy, especially under apparent sympathetic stimulus.^52^ CITED4 overexpression in cardiomyocytes was recently shown to be sufficient for the induction of cardiac hypertrophy and reduction of autophagy, reduced adverse cardiac remodeling, and reduced fibrosis after ischemic injury.^24^ Interestingly, we detected rapid induction of cardiomyocyte hypertrophy by ACVR2B-Fc in IR hearts, which was accompanied by upregulation of CITED4.

### ACVR2B-Fc Contributes to Ischemia Protection by Regulation of Cardiomyocyte Metabolism

Approaches to alleviate IR injury aim at pharmaceutical compounds that reduce fatty acid uptake into mitochondria, inhibit mitochondrial fatty acid oxidation, or increase glucose oxidation.^53^ Blocking myostatin signaling in the heart by genetically inactivating myostatin from cardiomyocytes results in enhanced glycolysis, augmented glycogen storage, and cardiac hypertrophy in adult mice.^54^ In that study, however, myostatin deletion led to LV dilatation, impaired cardiac function, and increased mortality in otherwise healthy mice.^54^ Authors of the study showed this metabolic switch leading to cardiac hypertrophy to be mediated by AMPK activation. Similarly, we found AMPK target acetyl CoA carboxylase to be phosphorylated by myostatin inhibition by ACVR2B-Fc in healthy hearts. Phosphorylation of acetyl CoA carboxylase downregulates fatty acid synthesis.^55^ However, we did not detect an increase in acetyl CoA carboxylase phosphorylation in IR hearts, suggesting that in contrast to healthy hearts, ACVR2B-Fc did not affect fatty acid synthesis in ischemic hearts.

Upregulation of PGC1α results in increased mitochondrial biogenesis and oxidative phosphorylation.^56^ PGC1α4 isoform that results from alternative promoter usage and splicing of the primary PGC1α transcript induces robust skeletal muscle hypertrophy without producing a metabolic phenotype similar to what PGC1α1 isoform produces.^57^ In our study, upregulation of PGC1α1 and PGC1α4 isoforms by ACVR2B-Fc (together with inhibition of GSK3β) at least partly explain the increased hypertrophy but may represent compensatory response to metabolic changes in the heart. Inhibition of myostatin signaling has also been shown to have beneficial metabolic effects in obesity and diabetes, including enhanced glucose tolerance, improved brown adipogenesis, and reduced fat mass.^7^, ^58^

Our data shows that ACVR2B-Fc treatment reduced metabolic activity in adult mouse cardiomyocytes after IR, reducing oxygen consumption. This resembles the phenomenon known as myocardial hibernation, in which the heart downregulates metabolism in order to adapt to ischemic conditions.^59^ At reperfusion, this may reduce the burst of reactive oxygen species and calcium overload in cardiomyocytes, and this approach has actually been suggested as a possible therapy for reperfusion injury. Basheer et al.^60^ recently demonstrated a similar cardioprotective phenotype in ischemic injury with pretreatment of mice with adenoviral infection of a mitochondrial targeting factor. The approach mimicked the cardioprotective effect of ischemic preconditioning by inducing metabolic quiescence and limiting production of damaging levels of reactive oxygen species in the mitochondria. Our results suggest ACVR2B-Fc may promote entering of the cardiomyocytes to an adaptive hibernating state that reduces energy substrate utilization and oxygen demand to match the oxygen availability in IR.

Finally, we determined whether the acute cardioprotective effect and metabolic changes induced by ACVR2B-Fc could provide beneficial effects on cardiac function after prolonged cardiac stress. Efficacy of ACVR2B-Fc on mitochondria function was determined in a cardiotoxicity model, as doxorubicin is known to alter cardiac metabolism.^25^ Besides the disruption of mitochondrial oxidative respiration, which includes inhibition of complex I activity, many other proteins in metabolic pathways, such as mitochondrial creatine kinases, are affected by doxorubicin.^25^ We found that treatment of mice with ACVR2B-Fc improved mitochondrial function and prevented the deterioration of cardiac systolic function in doxorubicin-treated mice. This suggests that, in addition to its acute pro-survival and metabolic effects in IR injury, ACVR2B-Fc has more broad beneficial effects on the heart.

### Cardiac Preconditioning by ACVR2B-Fc Is Required for Full Cardioprotection

Activin A and mature myostatin reside in a latent complex extracellularly, ready to bind ACVR2B when released from their binding peptides. Activin and myostatin activities are endogenously inhibited by follistatin or follistatin-like proteins or, in case of myostatin, by GDF-associated serum proteins that bind to ligands and neutralize their effects. Activins are also inhibited by inhibins that interfere ACVR2A/ACVR2B binding to type I receptors and activation of intracellular signal transduction cascades such as SMAD2/3.

We chose to study ACVR2B-Fc as a therapeutical approach due to its benefits in hypertrophic muscle growth and its broader ligand specificity. ACVR2B-Fc blocks signaling of myostatin, its close homolog GDF11, as well as activin A, activin B, and BMP10.^2^ Inhibition of myostatin signaling by ACVR2B-Fc has been studied in muscle-wasting conditions. Myostatin signaling blockade has been achieved by specific antibodies,^61^ by antibodies targeted to ACVR2,^62^ ligand traps including ACVR2B-Fc,^18^, ^19^, ^20^, ^63^, ^64^ or by overexpression of natural inhibitors such as follistatin.^40^, ^42^ However, myostatin and other TGF-β family members, especially GDF11 and activin A, share a high degree of similarity in receptor recognition sites thus lacking target specificity. Recently, human monoclonal antibodies to pro-myostatin and pro-GDF11 were developed.^65^ These inhibit their targets by blocking growth factor release from the prodomain and work with higher specificity, since pro-domains are much less conserved than mature domains. It remains to be shown whether pro-domain targeting approach, which reduced glucocorticoid-induced muscle wasting,^65^ could also work in protection from IR injury and whether it offers extra benefits when inhibiting myostatin specifically.

To conclude, the changes described in this study, which improve cardiomyocyte response to hypoxia, predominantly require ACVR2B-Fc to be administered prior to IR. Although administration of ACVR2B-Fc at reperfusion inhibits canonical SMAD2/3 and non-canonical pathways, this is sufficient to only partially inhibit pathological pathways. Therefore, transcriptional modification toward metabolically optimized cardiac function is needed to achieve full cardioprotective effect of ACVR2B-Fc. Our findings resemble ischemic preconditioning, which is known to protect the heart from IR injury and is associated with priming of mitochondria into a metabolically altered stress-resistant state. According to our results, systemic blockade of ACVR2B ligands was sufficient to promote protection from IR in the heart. In addition to ACVR2B, myostatin and activin A also signal through ACVR2A receptors. In a recent study, dual blockade of ACVR2A/ACVR2B by bimagrumab was shown to be required for full anabolic response in skeletal muscle.^3^ It remains to be shown whether dual blockade of ACVR2 receptors could offer extra benefit in treatment of IR injury.

## Materials and Methods

An expanded methods section is provided in the Supplemental Information. Experimental protocols were approved by the Animal Use and Care Committee of the University of Oulu and the national Animal Experiment Board of Finland. 8- to 10-week-old male C57BL/6J mice were anaesthetized with isoflurane and subjected to IR by ligation of the left anterior descending coronary artery (LAD) for 30 min, after which the slip knot was released, allowing reperfusion of the ischemic myocardium for 6 or 24 h, as previously described.^66^ The experimental timeline is depicted in Figure S1. ACVR2B-Fc recombinant fusion protein^18^ was administered as 10 mg/kg subcutaneously (s.c.) 24 h prior to IR and at reperfusion (termed “ACVR2B-Fc pretreatment”) or only at reperfusion (termed “ACVR2B-Fc at reperfusion”). Data are expressed as mean ± SD. *p < 0.05, **p < 0.01 and ***p < 0.001.

## Author Contributions

J.M. participated in experimental studies, analyzed the results, and wrote the manuscript. L.V., T.K., and Z.S. performed experimental studies and participated in analysis of the results. R.L. and L.R.-K. performed histological analysis. J.J.H., M.R., K.A., and R. Kivelä designed, performed, and/or analyzed experimental studies on cardiotoxicity. E.G., W.J.K., and R. Kerkelä designed and/or performed experimental IR studies. S.T., T.A., and J.U. performed *in vitro* studies. M.L., A.P., and O.R. designed and produced the pharmacological agent and participated in design of the study. J.J.H., L.V., R. Kivelä, and R. Kerkelä critically revised the manuscript. All authors have read and approved final manuscript.

## Conflicts of Interest

The authors declare no competing interests.

## Supplemental Information

Document S1. Figures S1–S4, Tables S1–S4, and Supplemental Materials and Methods

Document S2. Article plus Supplemental Information
