# Supplementary material for: Systemic Blockade of ACVR2B Ligands Protects Myocardium from Acute Ischemia-Reperfusion Injury
Source: Mol Ther. 2019 Jan 24;27(3):600–10. doi: 10.1016/j.ymthe.2019.01.013 (PMC6404100; doi:10.1016/j.ymthe.2019.01.013)
Supplement: Document S1. Figures S1–S4, Tables S1–S4, and Supplemental Materials and Methods [file mmc1.pdf]

YMTHE, Volume *27*

## Supplemental Information

# Systemic Blockade of ACVR2B Ligands Protects Myocardium from Acute Ischemia-Reperfusion Injury

**Johanna Magga, Laura Vainio, Teemu Kilpiö, Juha J. Hulmi, Saija Taponen, Ruizhu Lin, Markus Räsänen, Zoltán Szabó, Erhe Gao, Lea Rahtu-Korpela, Tarja Alakoski, Johanna Ulvila, Mika Laitinen, Arja Pasternack, Walter J. Koch, Kari Alitalo, Riikka Kivelä, Olli Ritvos, and Risto Kerkelä**

## Supplemental Methods and Materials

### Figure S1.

(**A**) Timeline for the IR study. (**B**) Echocardiography analysis at 24h after IR injury. Representative M mode echocardiography images are shown. Mice were treated with vehicle, soluble decoy receptor ACVR2B-Fc only at reperfusion or pretreatment of ACVR2B-Fc 24h prior to/at reperfusion. IR was achieved by transient ligation of the left anterior descending coronary artery for 30 min, followed by reperfusion for 24h.

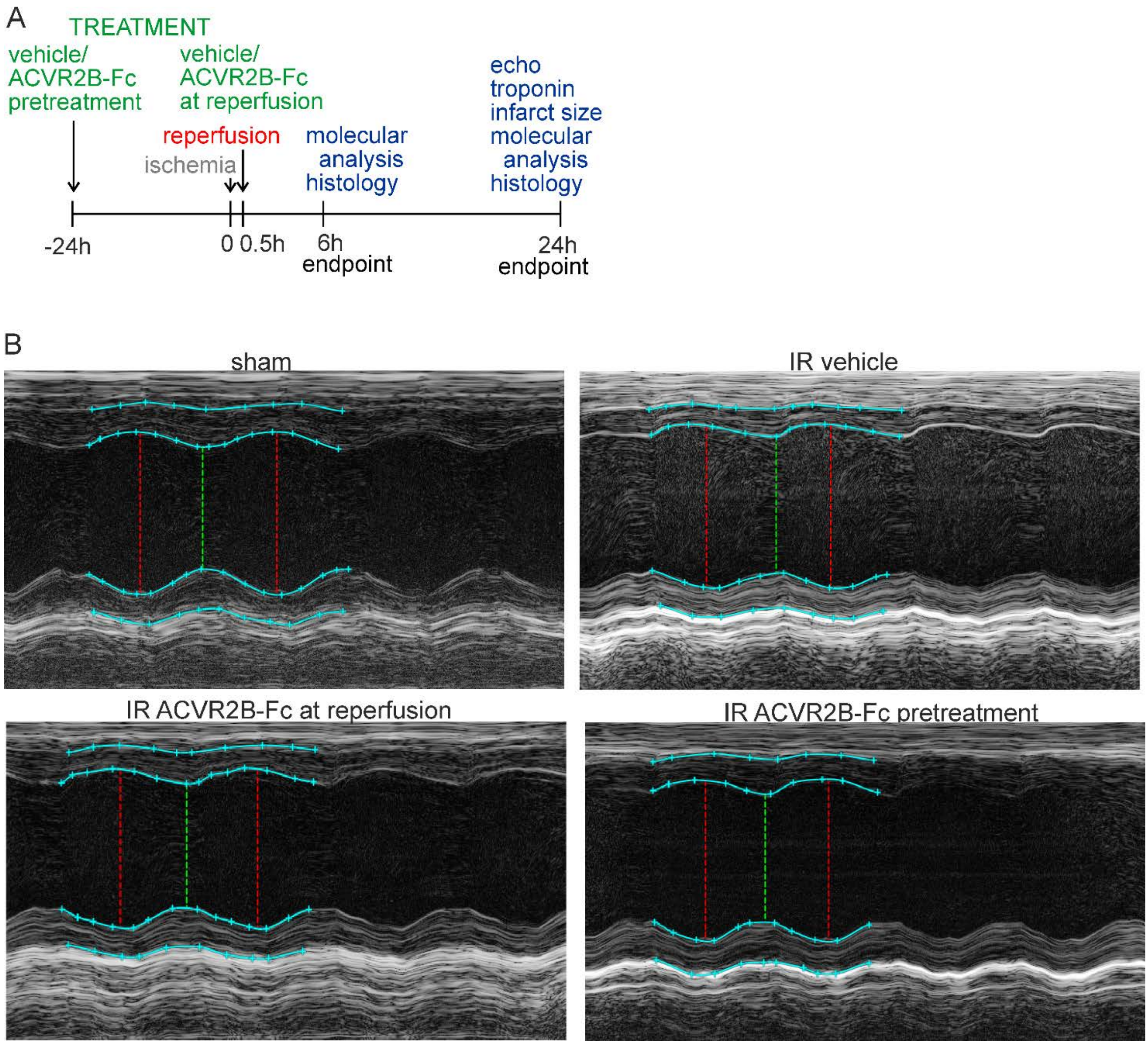

**Figure S2.**

(**A)** ACVR2B treatment (single dose of 10 mg/kg s.c.) for 48h increased cardiomyocyte cross-sectional area in left ventricles of the healthy mice. (**B**) ACVR2B increases skeletal muscle cross-sectional area in the quadriceps of the healthy mice. (**C**) ACVR2B-Fc induced hypertrophy was accompanied by increased, but transient phosphorylation of GSK3β at Ser9. (**D**) ACVR2B-Fc reduced apoptosis in the left ventricles 6h after IR injury as analyzed by TUNEL. (E) TUNEL positive nuclei were localized in cardiomyocytes stained with sarcomeric α-actinin. Scale bar 50 µm. n=5 and 6 (A, C), n=4 and 5 (B). Data are presented as mean ± SD. *P<0.05, **P<0.01.

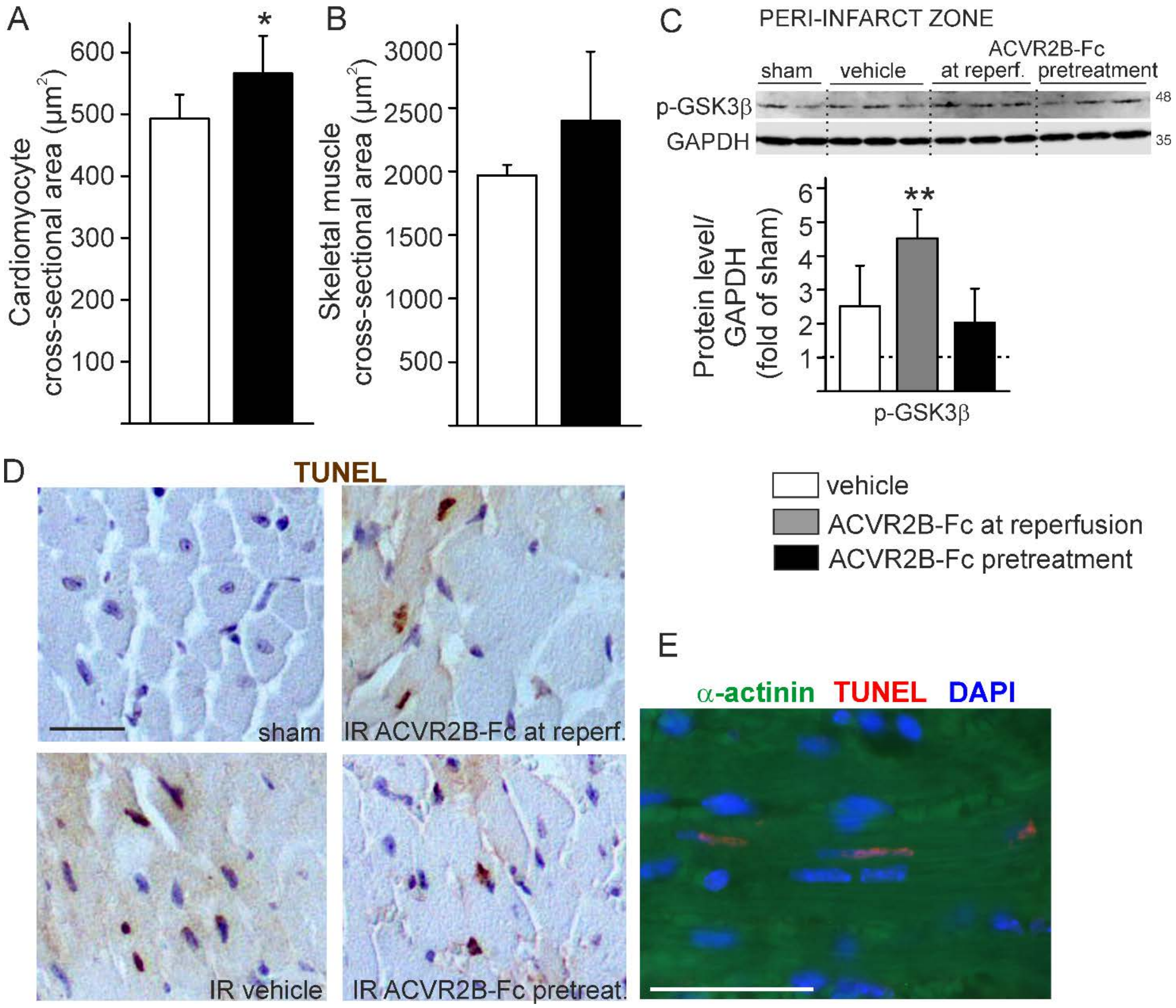

**Figure S3.**

(**A**) ACVR2B-Fc did not affect infiltration of inflammatory cells into LV at 24h after IR as analyzed with granulocyte stain. (**B**) ACVR2B-Fc did not affect inflammatory response at 6 or 24h after IR as analyzed with qPCR from left ventricle. Scale bar 50 µm. n=5, 6 (A, 6h IR), n=10, 6 (A, 24h IR), n=5, 6 (B, 6h IR), n=9, 8 (B, 24h IR). Data are presented as mean ± SD.

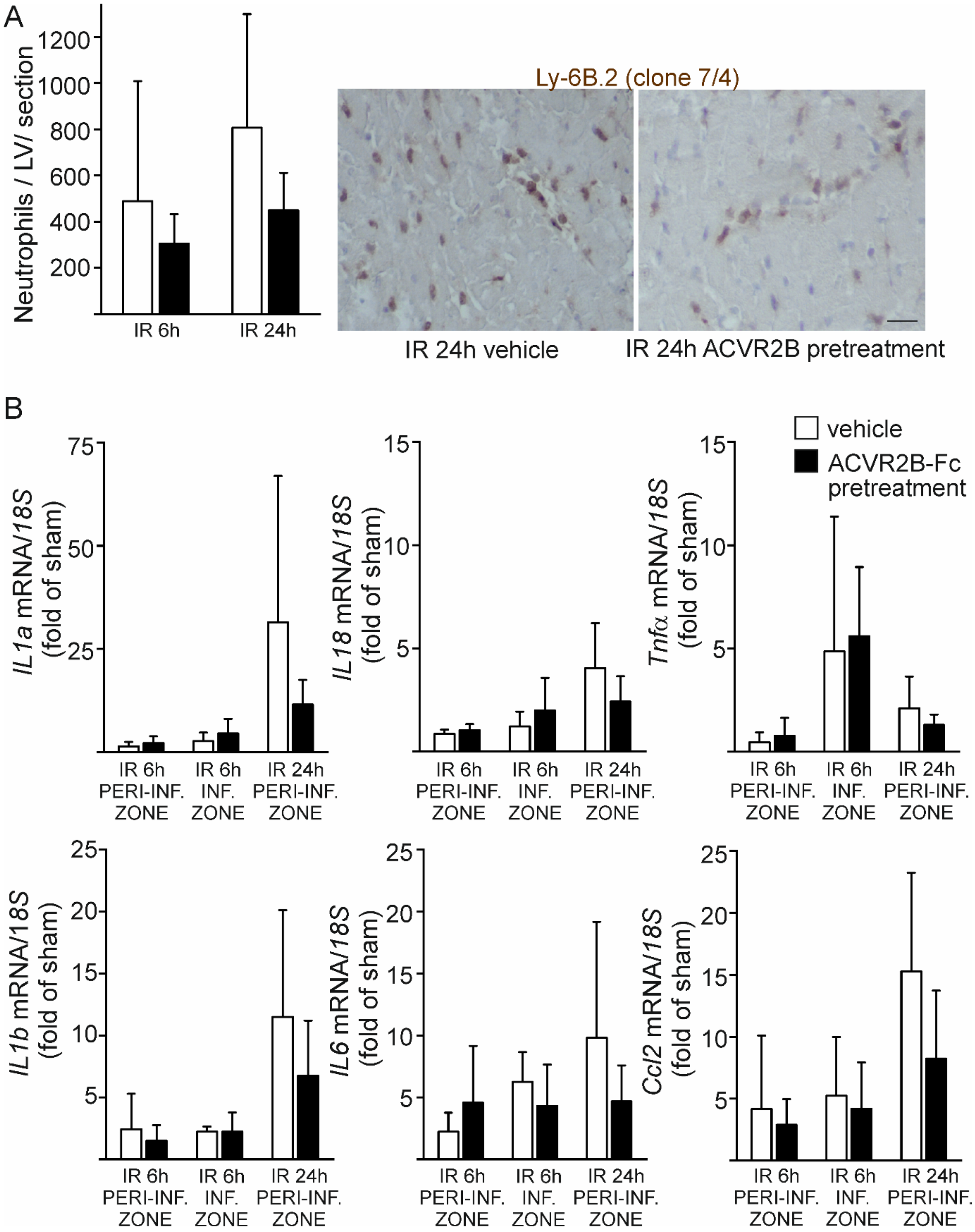

**Figure S4.**

(**A**) ACVR2B ligands did not induce BRE-luc SMAD activity unlike BMPR ligand BMP4 as determined in neonatal rat ventricular cardiomyocytes. (**B-C**) ACVR2B ligands activin A, activin B and myostatin levels were increased in LV infarct and peri-infarct area, but not remote area, 6h and 24h after IR. (**D**) ACVR2B-Fc increased phosphorylation of acetyl CoA-carboxylase in sham-operated mice but not in IR-operated mice analyzed 24h after IR and 48h after ACVR2B-Fc treatment. Scale bar 50 µm. n=6 (A). Data are presented as mean ± SD. *P<0.05, ***P<0.001.

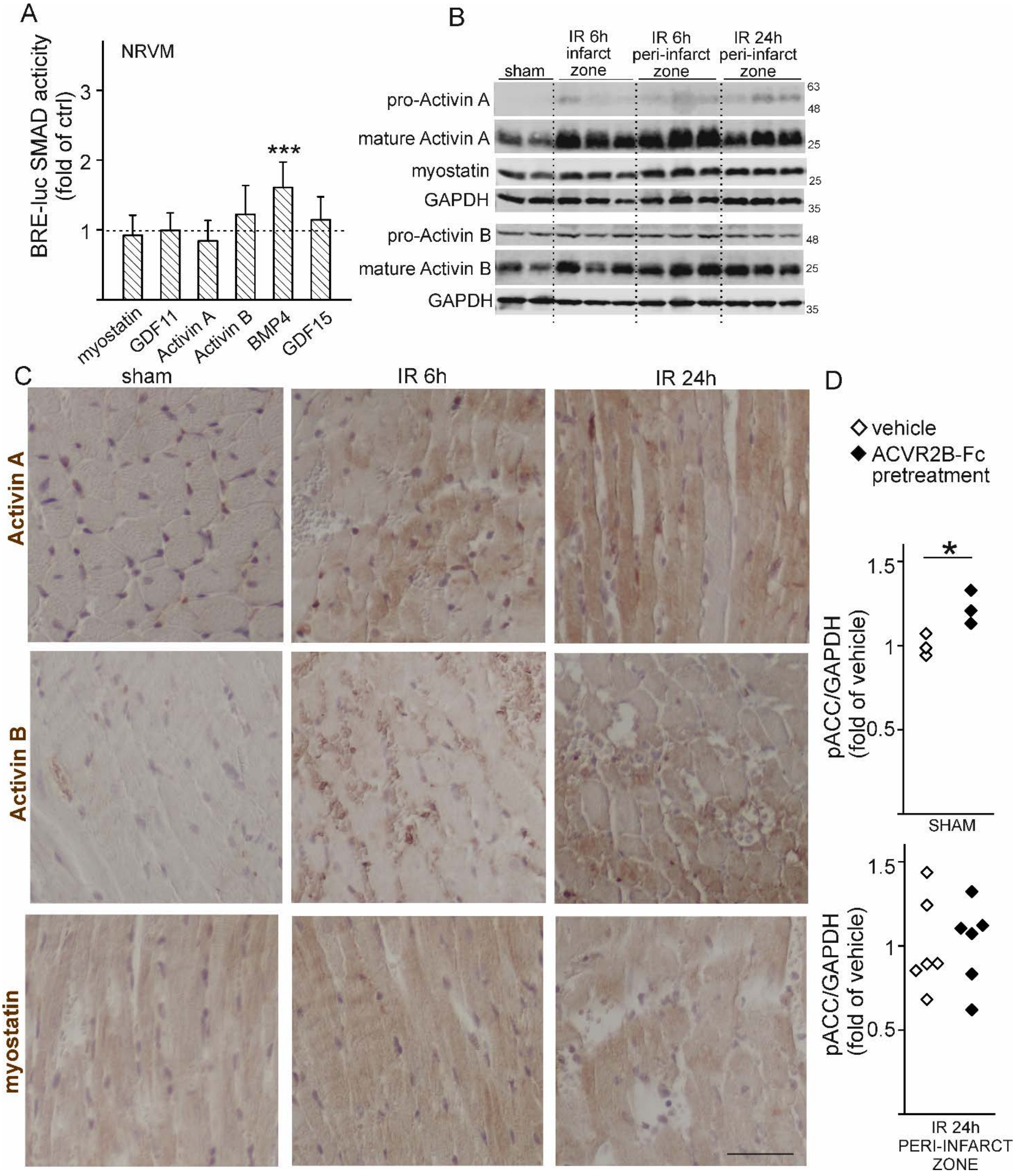

**Table S1.**

Oligonucleotide primer and detection probe sequences used for mRNA quantitation by qPCR.

| Gene | Sequences<br>Fluorogenic Probe (5’-[FAM…TAMRA]) | Sense (5’-…) | Antisense (5’-…) |
|---|---|---|---|
| **Activin A (Inhba)** | - | GAACGGGTATGTGGAGATAG | TGAAATAGACGGATGGTGAC |
| **Activin B (Inhbb)** | - | CTTCGTCTCTAATGAAGGCAACC | CTCCACCACATTCCACCTGTC |
| **Acvr2A** | - | GTTACACCGAAGCCACCCTA | AACCAAATCTTCCCCTTGCT |
| **Acvr2B** | - | TTAAGGATCACTGGCTGAAACA | GGATACCCGCTCTTCTACACAG |
| **ANP (Nppa)** | TCGCTGGCCCTCGGAGCCT | GAAAAGCAAACTGAGGGCTCTG | CCTACCCCCGAAGCAGCT |
| **Bmpr2** | - | TTGGACTCATCTACTGGGAGGT | TGGACACAAGAACCTGCATATC |
| **BNP (Nppb)** | CATCATTGCCTGGCCCATCGC | AGGCGAGACAAGGGAGAACA | GGAGATCCATGCCGCAGA |
| **Ccl2** | CACTCACCTGCTGCTACTCATTCACTGGC | CTCAGCCAGATGCAGTTAATGC | AGCCGACTCATTGGGATCAT |
| **Cited4** | - | CCTGGCATACGGCTCCTTC | AGACTGCAGGTGCGTGCTAC |
| **Col1a1** | CTTTGCTTCCCAGATGTCCTATGGCTATGATG | CCCTGGCCTTGGAGGAA | CACGGAAACTCCAGCTGATTTT |
| **Col3a1** | TGGTGAACGTGGCTCTAATGGCATCA | CCACGAGGTGACAAAGGTGA | GCCAGGGAATCCTCGATGT |
| **Cytohrome C (Cycs)** | . | GGAGGCAAGCATAAGACTGG | TCCATCAGGGTATCCTCTCC |
| **Gapdh** | CTGCCGATGCCCCCATGTTTG | GGTCATCATCTCCGCCCC | TTCTCGTGGTTCACACCCATC |
| **Gdf11** | - | CTTGGAAGAGGACGAGTACCAC | CTGAAGTGGAAATGACAGCAGA |
| **Glut1** | - | CTCTGTCGGCCTCTTTGTTAAT | CCAGTTTGGAGAAGCCCATAAG |
| **Glut4** | - | GTGACTGGAACACTGGTCCTA | CCAGCCACGTTGCATTGTAG |
| **IL1a** | - | CGAAGACTACAGTTCTGCCATT | GACGTTTCAGAGGTTCTCAGAG |
| **IL1b** | - | TCTTTGAAGTTGACGGACCC | TGAGTGATACTGCCTGCCTG |
| **IL6** | CAGAATTGCCATTGCACAACTCTTTTCTCA | ACATGTTCTCTGGGAAATCGTGGAA | TGCATCATCGTTGTTCATACAA |
| **IL18** | - | GACTCTTGCGTCAACTTCAAGG | CAGGCTGTCTTTTGTCAACGA |
| **MHCα (Myh6)** | CGAGGAATAACCTCTCCAGCAGACCCTC | GGTGCCAAGAAGATGCACG | TTATGTTTATTGTGTATTGGCCACAG |
| **MHCβ (Myh7)** | AGCCCTCAGACCTGGAGCCTTTGC | AGCTCTAAGGGTGCCCGTG | TGCTTCCACCTAAAGGGCTG |
| **Mstn** | - | AAGATGGGCTGAATCCCTTT | GCAGTCAAGCCCAAAGTCTC |
| **Pfkm** | CTGACACAGCACTGAACACC | CGTCCCTGGGTCAGACTTC | CAGACTGCTTGATTCGGTCAC |

| | | | |
|---|---|---|---|
| | ATCTGCAC | AG | A |
| **Pgam1** | CCCTTCTACAGCAACATCAGCAAGGATC | TTATGATGTCCCACCGCCT | GGTCTTCAGTAAGGTCTGCGTACCT |
| **Pgc1a1** | - | GGACATGTGCAGCCAAGACTCT | CACTTCAATCCACCCAGAAAGCT |
| **Pgc1a4** | - | TCACACCAAACCCACAGAAA | CTGGAAGATATGGCACAT |
| **Tgfβ1 (Tgfb1)** | ACGGAAGCGCATCGAAGCCATC | CATCGACATGGAGCTGGTGA | TTGGACAGGATCTGGCCAC |
| **Tnfα (Tnf)** | TGCTCCTCACCCACACCGTCAGC | GACAAGGCTGCCCCGACTA | CTCCTGGTATGAGATAGCAAATC |
| **18S** | CCTGGTGGTGCCCTTCCGTCA | TGGTTGCAAAGCTGAAACTTAAAG | AGTCAAATTAAGCCGCAGGC |
| **18S** | - | CGCCGCTAGAGGTGAAATTC | CCAGTCGGCATCGTTTATGG |

**Table S2.** ACVR2B-Fc preserves cardiac function in ischemia-reperfusion injury.

Mice were subjected to a sham operation or ischemia-reperfusion injury (IR) and treated with either vehicle or ACVR2B-Fc only at reperfusion or pretreatment with ACVR2B-Fc 24h prior to/at reperfusion. After 24h mice were subjected to echocardiography analysis. Ejection fraction (EF), fractional shortening (FS), heart rate (HR), left ventricular (LV) mass, LV internal diameter in diastole (LVID;d) and systole (LVID;s), LV posterior wall thicknesses (LVPW;d, LVPW;s), endocardial areas (ENDO;d, ENDO;s) and endocardial fractional area change (ENDO-FAC). Data are presented as mean±SD; *P<0.05, ** P<0.01, *** P<0.001 versus sham, # p<0.05, ## p<0.01, ### p<0.001 versus IR vehicle.

| | **sham**<br>**n=13** | **IR**<br>**vehicle**<br>**n=19** | **IR**<br>**ACVR2B-Fc**<br>**at reperfusion**<br>**n=9** | **IR**<br>**ACVR2B-Fc**<br>**pretreatment**<br>**n=18** |
|---|---|---|---|---|
| **EF (%)** | 50.4±8.9 | 36.4±11.9** | 39.1±7.4 | 48.7±11.2## |
| **FS (%)** | 25.6±5.4 | 17.6±6.5** | 18.8±4.1 | 24.5±6.7## |
| **HR (BPM)** | 415±25 | 460±48 | 477±35* | 445±66 |
| **LV mass (mg)** | 91±18 | 96±20 | 96±12 | 105±21 |
| **LVID;d (mm)** | 4.19±0.25 | 4.36±0.36 | 4.04±0.25# | 4.00±0.33## |
| **LVID;s (mm)** | 3.12±0.33 | 3.61±0.51* | 3.29±0.33 | 3.03±0.44### |
| **LVPW;d (mm)** | 0.70±0.07 | 0.72±0.17 | 0.75±0.03 | 0.84±0.11*# |
| **LVPW;s (mm)** | 0.99±0.16 | 0.89±0.29 | 0.94±0.08 | 1.11±0.18*# |
| **ENDO;d ($mm^2$)** | 12.6±1.2 | 13.5±1.9 | 12.8±1.1 | 12.0±1.8# |
| **ENDO;s ($mm^2$)** | 7.1±1.3 | 9.5±2.3** | 8.8±1.3 | 7.3±2.2## |
| **ENDO-FAC (%)** | 43.4±6.9 | 30.1±9.0*** | 31.7±6.8* | 40.3±11.9## |

**Table S3.** Blood cell count of ACVR2B-Fc and vehicle-treated mice.

Mice were treated with vehicle or ACVR2B-Fc 24h prior to/at reperfusion and subjected to ischemia-reperfusion injury (IR). After 24h blood was collected and analyzed with veterinary hematology analyzer for red blood cell analysis and white blood cell differential count. Red blood cell count (RBC), mean cell volume (MCV), hematocrit (HCT), mean cell hemoglobin (MCH), MCH concentration (MCHC), RBC distribution width (RDWR), RDWR absolute volume (RDWA), hemoglobin (HGB), white blood cell count (WBC), absolute counts for lymphocytes (LA), monocytes (MA) and granulocytes (GA). Data are presented as mean±SD.

| | **IR vehicle n=8** | **IR ACVR2B-Fc pretreatment n=6** |
|---|---|---|
| **RBC ($10^9$/ml)** | 8.3±0.4 | 8.3±1.2 |
| **MCV (fL)** | 39.4±0.2 | 39.2±0.4 |
| **HCT (%)** | 32.6±1.6 | 32.4±5.0 |
| **MCH (pg)** | 15.7±0.2 | 15.7±0.2 |
| **MCHC (g/dl)** | 39.8±0.4 | 40.0±0.7 |
| **RDWR (%)** | 19.9±0.3 | 19.7±0.2 |
| **RDWA (fL)** | 26.4±0.3 | 26.0±0.6 |
| **HGB (g/dl)** | 13.0±0.6 | 13.0±1.9 |
| **WBC ($10^6$/ml)** | 6.1±1.7 | 5.0±1.9 |
| **LA ($10^6$/ml)** | 4.6±1.4 | 3.2±1.0 |
| **MA ($10^6$/ml)** | 0.35±0.12 | 0.32±0.12 |
| **GA ($10^6$/ml)** | 1.14±0.44 | 1.53±0.87 |

**Table S4.** ACVR2B-Fc preserves cardiac function in doxorubicin-induced cardiomyopathy.

Mice were treated with doxorubicin for two weeks to induce cumulative cardiotoxicity, followed by two additional weeks to develop drug-induced cardiomyopathy. Mice were treated with ACVR2B-Fc or vehicle throughout 4-week study, and subjected to echocardiography analysis. Ejection fraction (EF), fractional shortening (FS), left ventricular (LV) mass, LV internal diameter in diastole (LVID;d) and systole (LVID;s) and LV posterior wall thicknesses (LVPW;d, LVPW;s). Data are presented as mean±SD; *P<0.05, versus control, # P<0.05 versus doxorubicin with vehicle.

| | **control**<br><br>**n=9** | **Doxorubicin vehicle**<br>**n=6** | **Doxorubicin ACVR2B-Fc**<br>**n=9** |
|---|---|---|---|
| **EF (%)** | 57.4±6.3 | 47.8±6.8* | 54.4±2.2# |
| **FS (%)** | 30.1±4.2 | 23.9±4.0* | 27.1±2.5 |
| **LV mass (mg)** | 109±19 | 91±25 | 91±12 |
| **LVID;d (mm)** | 4.36±0.35 | 4.25±0.23 | 4.16±0.15 |
| **LVID;s (mm)** | 3.05±0.38 | 3.23±0.23 | 3.03±0.16 |
| **LVPW;d (mm)** | 0.78±0.07 | 0.74±0.14 | 0.75±0.10 |
| **LVPW;s (mm)** | 1.13±0.09 | 0.97±0.15 | 1.05±0.11 |

## Supplemental Methods

### Ischemia-reperfusion

Experimental protocols were approved by the Animal Use and Care Committee of the University of Oulu and the Regional State Administrative Agency of Southern Finland (ESAVI-2010-03931/ Ym-23). The mice were maintained in plastic cages at a constant 21°C temperature with a 12 h light–dark cycle and had free access to food (Teklad Global Rodent diet, Harlan) and water. Immediately after operation, mice were kept in warm room at 25°C temperature overnight.

8-10 week old male C57BL/6J mice (Harlan) were subjected to ischemia-reperfusion (IR) by ligation of the left anterior descending coronary artery (LAD) for 30 minutes, after which the slip knot was released allowing reperfusion of the ischemic myocardium for 6 or 24h as previously described.[1] Male mice were used for the study to have more homogenous population for quantitation of the IR injury. Sham-operated mice were subjected to the same surgical procedure without ligation of the LAD. All operations were performed under isoflurane anaesthesia (Baxter, Vetequip evaporiser, 2% isoflurane with 1 l/min oxygen flow). Carprofen (Rimadyl) 5 mg/kg s.c. (injection volume 0.125-0.15 ml) and buprenorphine (Vetergesic) 0.05 mg/kg s.c. (injection volume 0.1-0.3 ml) were administred as peri-operation analgesia. Post-operation dehydration was prevented with s.c. administration of 5% glucose solution (injection volume 0.5-1.0 ml). All animals were monitored after the surgery and received a dose of buprenorphine (0.05 mg/kg) in the evening of the operation day. Another dose of buprenorphine and a dose of carprofen (5mg/kg) were administered the following morning.

### ACVR2B-Fc treatment

ACVR2B-Fc recombinant fusion protein was produced as described earlier.[2] The ectodomain of human ACVR2B was fused with a human IgG1 Fc domain and expressed in Chinese hamster ovary cells grown in suspension culture. The protein is similar but not identical to that originally generated by Lee et al.[3]. ACVR2B-Fc was administered as 10 mg/kg s.c. 24h prior to IR and at reperfusion (termed “ACVR2B-Fc pretreatment”) or only at reperfusion (termed “ACVR2B-Fc at reperfusion”). The same volume of PBS or human IgG1-Fc control was used as a vehicle control. Experimental timeline is depicted in Figure S1.

The littermates were randomly and equally divided into treatment groups to avoid any bias between the mouse litters. *6h endpoint:* In 6h endpoint group, one mouse from IR vehicle group died at operation. Group sizes for the analysis were following: sham n=6, IR vehicle n=5, IR ACVR2B-Fc pretreatment n=6, ACVR2B-Fc at reperfusion n=6. *24h endpoint:* In the 24h endpoint group, one mouse from IR vehicle group and 4 mice from ACVR2B-Fc pretreatment group died during the operation. Group sizes for the analysis were following: sham n=13, IR vehicle n=19, IR ACVR2B-Fc pretreatment n=18, ACVR2B-Fc at reperfusion n=9. All mice in 24h endpoint were subjected to echocardiography. A subgroup of sham n=5, IR vehicle n=9, IR ACVR2B-Fc pretreatment n=10 mice were used for cardiac troponin I assay and infarct size determination by TTC stain. The rest of the mice were used for qPCR, western blotting and histological analysis. The person operating on the mice was blinded from treatment. All analysis to assess functional, histological and other outcome were performed in a blinded manner.

## Echocardiography

Mice were anaesthetized with isoflurane and transthoracic echocardiography was performed with Vevo 2100 high frequency, high resolution linear array ultrasound system using a MS-550S transducer (Visual Sonics Vevo 2100, 40 MHz, axial resolution 40 µm, lateral resolution 90 µm). B-mode, M-mode, transmitral flow - Pulse wave and tissue Doppler images were recorded and analyzed with Vevo Workstation software 1.7 by a blinded observer.

## Determination of infarct size

Determination of area at risk (AR) and infarct size was performed 24h after IR as described.[1] Mice were reanaesthetized and the ligature around the LAD was retied at the previous ligation site. Following this, 2% Evans blue dye in PBS was injected into the aorta and allowed to circulate uniformly into areas of the heart perfused by the open coronary arteries. The heart was then quickly excised, snap frozen on dry ice and cut into five sections from the apex to the base. Sections were then incubated in 1% triphenyltetrazolium chloride (TTC) (Sigma) solution in PBS 20 min at 37°C, fixed with phosphate-buffered 10% formalin (pH 7.0) for 20 min, rinsed in PBS and photographed. The area not at risk (ANAR; Evans blue-stained area), AR (TTC staining-positive, non-infarct, red) and infarct (TTC staining-negative, white) areas were quantified with the Nikon NIS-Elements BR 2.30 program. Blood samples were collected 24h after IR and cardiac troponin I was measured from plasma with high-sensitivity mouse cardiac troponin I ELISA kit (Life Diagnostics) according to manufacturer's instructions.

## Cardiomyocyte cell cultures

*Hypoxia and cell survival assays in adult ventricular cardiomyocytes*

Adult mouse ventricular cardiomyocytes (AMVMs) and rat ventricular cardiomyocytes (ARVMs) were isolated from 8-12 week old male C57BL/6 mice or SD rats by retrograde perfusion and enzymatic digestion as described before.[4] Mice were deeply anesthetized with isoflurane. Rats were euthanized with $CO_2$, decapitated and after the excision of the heart aorta was cannulated. For retrograde perfusion, a perfusion buffer (Tyrode's buffer; 24.1 mM HEPES, 127.5 mM NaCl, 5.1 mM KCl, 0.4 mM $NaH_2PO_4$, 0.55 mM $MgCl_2$) supplemented with 10 mM 2,3-butandione monoxime (BDM, Sigma) and 5.5 mM glucose (Sigma) was used at a flow rate of 3 ml/min/mouse heart or 5 ml/min/rat heart for 1 min before switching to perfusion buffer with 1 mg/ml collagenase type 2 (Worthington) and 12.6 µM $CaCl_2$ (Sigma) and perfused at a flow rate of 3ml/min/mouse heart for 8 min or 10 ml/min/rat heart for 18 min. Then hearts were cut into small pieces and collected to a tube containing perfusion buffer with 5 % FBS, where gently homogenized with trituration. The suspension was filtered through 200 µm mesh before cardiomyocytes were pelleted (300 rpm (18G), 2 min) and resuspended into perfusion buffer including 5 % FBS. $Ca^{2+}$ concentration was gradually increased to 1.26 mM. After $Ca^{2+}$ reintroduction cardiomyocytes were pelleted and resuspended in plating medium (AMVM: αMEM with Hank's salt (21575-022, Thermo Fisher Scientific) supplemented with 5 % FBS, insulin-transferrin-selenium supplement (Thermo Fisher Scientific), 10 mM BDM, 2 mM L-glutamine (Sigma) and penicillin-streptomycin (Sigma); ARVM: αMEM with Earle's salt (10370-047, Thermo Fisher Scientific) supplemented with 5 % FBS, 10 mM HEPES, insulin-transferrin-selenium supplement, 10 mM BDM, 2 mM L-glutamine and penicillin-streptomycin. Cardiomyocytes were plated onto laminin-coated wells at

12 500 cells/cm$^2$ and incubated in humidified atmosphere at 37°C containing 2% or 5% $CO_2$ for AMVM and ARVM, respectively. After 2h incubation, nonattached cells were gently removed.

For ARVM hypoxia experiment on the following day, medium was changed to αMEM with Earle's salt supplemented with insulin-transferrin-selenium, 10 mM BDM and 2 mM L-glutamine. Oxygen levels were controlled at 0.1% $O_2$ in 5% $CO_2$ for 4h, then medium was changed back to normal cultivation medium in normoxic conditions for reperfusion. Control cells were treated with same media in normoxic conditions. To assess the efficacy of ACVR2B-Fc in hypoxia, 200 ng/ml ACVR2B-Fc or control IgG1-Fc was added 30 min before hypoxia and to reperfusion medium. Medium samples were collected 4h after reperfusion for cell toxicity assay while cell viability was assessed 20h later.

To determine the effect of ligands on cell viability in hypoxic conditions, 100 ng/ml myostatin, GDF11, activin A, activin B, TGFβ or GDF15 were applied for cells 30 min before hypoxia. Thereafter, ARVM were exposed to 4h hypoxia followed by 4h reperfusion as described above. Medium samples were collected 4h after reperfusion for cell toxicity assay while cell viability was assessed 20h later.

Cell toxicity was determined with Toxilight (Lonza) to measure cell membrane integrity by quantifying adenylate kinase released into the medium. Cell viability was measured with resazurin assay (Sigma) which quantifies the reduction of resazurin to a fluorescent compound resorufin within viable cells. For analysis, 10 µM resazurin (Sigma) was added to the cell culture medium and 1h later, medium samples were collected into 96-well plate and measured by excitation at 544 nm and emission at 595 nm on a multiplate reader (Victor Wallac).

*Luciferase reporter assays and hypoxia in neonatal rat ventricular cardiomyocytes*

Neonatal rat ventricular cardiomyocytes (NRVMs) were isolated from 2-4 day-old Sprague-Dawley rats as described earlier.[5] Rats were sacrificed by quick decapitation, the hearts were excised, the ventricles were minced and digested in 2 mg/ml collagenase type 2 (Worthington) in PBS supplemented with 50 µM $CaCl_2$. Freshly isolated cells were preplated in DMEM/F12 supplemented with 10% fetal bovine serum (FBS) and with penicillin-streptomycin for 2h to remove fibroblasts. Nonattached cells were collected, plated at 150 000 cells/cm$^2$ and incubated overnight before changing to serum-free culture media (CSFM; DMEM/F12, insulin-transferrin-selenin, 1 mM sodium pyruvate, 0.25% bovine serum albumin (BSA), 1 nM $T_3$, penicillin-streptomycin). 1-2 days after isolation, NRVMs were transfected with CAGA-luc[6] or BRE-luc luciferase constructs[7] (1.78 µg/ml) or control plasmid pEF-IRESp, combined with pRL-TK Renilla luciferase control reporter (0.89 µg/ml) with Lipofectamine (1:100, Invitrogen) in OptiMEM and incubated for 6h. Two days after transfection, cells were treated with 100 ng/ml myostatin, GDF11, activin A, activin B, TGFβ or GDF15 (Peprotech) for 2h. Thereafter, cells were rinsed with PBS and frozen at -70°C. Luciferase was measured with Dual Luciferase Reporter Assay System (#E1910, Promega) with Luminoskan microplate luminometer (Thermo Fisher Scientific). To determine SMAD-promoter activity, Firefly luciferase activity was divided by Renilla luciferase activity.

For NRVM hypoxia experiment, medium was changed to DMEM (31885-023, Thermo Fisher Scientific), supplemented with 2% FBS, penicillin-streptomycin after luc-transfection. Two days after transfection, NRVM were incubated in DMEM (11966-025, Thermo Fisher Scientific) supplemented with 10 mM deoxyglucose and 1 mM sodium dithionite (Fluka), placed in C-Chamber with oxygen levels controlled at 0.1% in 5% $CO_2$ with ProOx C21 $O_2$/$CO_2$ controller (BioSpherix). Control (normoxia) cells were incubated in DMEM supplemented with 10 mM

glucose. 200 ng/ml ACVR2B-Fc or control IgG1-Fc was added 30 min before hypoxia. SMAD-promoter activity was measured with luciferase reporter assay 4 h after hypoxia as described above. Gene expression changes in NRVM were analyzed with qPCR after 4h hypoxia followed by 5h reperfusion.

*Bioenergetic assay for adult mouse ventricular cardiomyocytes*

Vehicle or ACVR2B-Fc (10 mg/kg s.c.) was administered to 8-10 week old male C57BL/6 mice 48h prior to AMVM isolation. Assessment of cell energy metabolism was performed by Seahorse XFp (Agilent Technologies). Primary AMVM were seeded onto 8-well microplates at a density of 1500 cells/well in plating medium. After a 2-hour pre-plating, the cells were washed with plating medium and then incubated in αMEM medium supplemented with insulin-transferrin-selenium supplement (Thermo Fisher Scientific), 10 mM BDM and 2 mM L-glutamine. The cells were then subjected to either 4 hours of hypoxia controlled at 0.1% $O_2$ in 2% $CO_2$, or normoxia in 2% $CO_2$. After hypoxia, the medium was immediately changed to plating medium and incubated for 1h in 2% $CO_2$. Seahorse assay medium (Agilent) was supplemented with 10 mM BDM, 2 mM L-glutamine, 10 mM glucose and 1 mM sodium pyruvate, pH adjusted to 7.4 with 0.1 mM NaOH. The experiments were then carried out as per Agilent Seahorse XFp cell mito stress test protocol using 1 µM oligomycin, 1.5 µM FCCP and 0.5 µM rotenone + antimycin A. Homogeneity of cell seeding density among wells was verified by light microscopy prior to the assay. The test protocol was modified to perform 2 min measurements instead of the standard 3 min in order to avoid consuming the media oxygen reserve and possible restriction of cell oxidative metabolism. The results were analyzed using Agilent Wave and Graphpad Prism 7.

**RNA isolation and quantitative real-time PCR**

Mice were euthanized with $CO_2$ and sacrificed by decapitation. Heart was excised, frozen in liquid nitrogen, and stored at -70°C. RNA was isolated with Trizol (Invitrogen). cDNA was synthesized from 500 ng of RNA with Transcriptor First Strand cDNA synthesis kit (Roche). The expression levels were evaluated on an ABI Prism 7700 Sequence Detection System (Applied Biosystems) as previously described.[8] Oligonucleotide primer and detection probe sequences used for mRNA quantitation are presented in Table S1.

**Western blotting**

Total protein content was extracted from LV peri-infarct zone as described earlier.[9] The protein concentrations were determined with colorimetric protein assay (Bio-Rad Laboratories). Protein samples (40 µg) were denatured at 97°C for 5 min and loaded on sodium dodecyl sulfate polyacrylamide gel electrophoresis (SDS-PAGE), run at 200 V and transferred to 0.45 µm Optitran BA-S 85 nitrocellulose membrane (Whatman, GE Healthcare) at 100 V for 2 h. The membranes were incubated for 1 h at RT in blocking buffer (Odyssey, LI-COR) diluted into Tris-buffered saline (TBS, 50 mM Tris, 200 mM NaCl, pH 7.4). Membranes were then incubated overnight with primary antibody pSMAD2 (Ser465/467) (Cell Signaling #3108, used as 1:1000 dilution), pJNK (Promega #V7931, 1:5000), p-p38 (Thr180/Tyr182) (Cell Signaling #9211, 1:1000), pERK44/42 (Thr202/Tyr204) (Cell Signaling #9106, 1:2000), pAkt (Ser473) (Cell Signaling #9271, 1:1000), pACC (Cell Signaling #3661, 1:1000), Bim (Cell Signaling #2933, 1:1000), LC3 (Sigma, #L7543, 1:1000), pGSK3β (Ser9) (Cell Signaling #9336, 1:1000), Activin A (AnshLabs 18/16, A1006, 1:5000), Activin B (AnshLabs 12/9A, A1005, 1:2500), myostatin (Abcam ab3239, 1:1000) or GAPDH (Millipore #MAB374, 1:500 000) in blocking buffer, washed with TBS-0.05% Tween-20 and incubated with secondary antibody (goat-anti-rabbit Alexa Fluor 680; Molecular Probes or

goat-anti-mouse IRDye 800; Rockland Immunochemicals) 1 h at RT. Antibody binding was detected by Odyssey Infrared Imaging System (LI-COR) and quantified using the public domain NIH Image program (developed at the U.S. National Institutes of Health, Bethesda, MD).

**Immunohistochemistry**

After excision of the heart, a transversal section of the LV was fixed overnight in phosphate-buffered 10 % formaline (pH 7.0) and thereafter prepared as paraffin-embedded sections. Five µm thick sections were cut from the mid-section of the heart at the level of the papillary muscles. For staining, sections were deparaffinized in xylene and dehydrated in graded ethanol series. The sections were stained with Masson's trichrome stain to visualize cardiomyocytes, cell nuclei and fibrous tissue. Cross-sectional myocyte cell size was quantified (Nikon NIS-Elements) as an average of 50 cardiomyocytes/section obtained from five representative fields of each section (with 20× objective) from epicardial and endocardial sides of the LV. Apoptotic cells were determined by terminal deoxynucleotidyl transferase –mediated dUTP nick end labeling (TUNEL) method according to manufacturer's instructions (ApopTag in situ apoptosis detection kit, Chemicon). Sections were stained with anti-neutrophil (7/4, Abcam ab53453) with EnVision Detection System (Dako) for secondary detection to determine the number of infiltrated granulocytes. LV sections were stained with Activin A (AnshLabs 18/16, A1006), Activin B (AnshLabs 12/9A, A1005) and myostatin (Abcam ab3239) to determine the levels of ACVR2B ligands in heart after IR.

**Doxorubicin treatment and analysis of mitochondrial function**

The long-term effects of ACVR2B-Fc on cardiac function were analyzed in cardiotoxicity model. Male C57BL/6J mice aged 9-10 weeks were treated with vehicle, doxorubicin (Sigma, doxorubicin hydrochloride D1515) or doxorubicin and ACVR2B-Fc. Doxorubicin was injected i.p. four times every third day for 2 weeks (each injection 6 mg/kg) followed by two additional weeks to develop drug-induced cardiomyopathy. The doxorubicin administration protocol mimics the treatment of human patients with low doxorubicin doses, which induces cardiotoxicity. Mice were treated with ACVR2B-Fc or vehicle throughout 4-week study, administered as 5 mg/kg twice a week during the first 2 weeks and once a week after that. Heart mitochondrial function was measured using high-resolution respirometry (OROBOROS Oxygraph-2k) after 2 weeks of doxorubicin treatment. Five milligrams of cardiac LV tissue was homogenized with a shredder and analyzed using carbohydrate SUIT-protocol as described before.[10] FCCP was used at a concentration of 1 µM (titrated at 0.5, 1 and 1.5 µM). To estimate whether the changes in mitochondrial function may be at the level of Krebs cycle, mitochondrial citrate synthase (CS) activity was measured from the heart. The effect of cumulative doxorubicin toxicity was assessed with qPCR and with echocardiography, performed after 4 weeks of treatment under isoflurane anesthesia with Vevo 2100 ultrasound system.

**Statistics**

Data are expressed as mean±SD. The data were analyzed with SPSS software using Student *t* test, Mann–Whitney *U* test, or one-way ANOVA when appropriate, followed by Dunnett or Tukey post hoc test. *$p<0.05$, **$p<0.01$ and ***$p<0.001$.

## Supplemental References


1. Gao, E., Lei, Y.H., Shang, X., Huang, Z.M., Zuo, L., Boucher, M. et al. (2010). A novel and efficient model of coronary artery ligation and myocardial infarction in the mouse. Circulation research *107*, 1445-1453.
2. Hulmi, J.J., Oliveira, B.M., Silvennoinen, M., Hoogaars, W.M., Ma, H., Pierre, P. et al. (2013). Muscle protein synthesis, mTORC1/MAPK/Hippo signaling, and capillary density are altered by blocking of myostatin and activins. Am. J. Physiol Endocrinol. Metab *304*, E41-E50.
3. Lee, S.J., Reed, L.A., Davies, M.V., Girgenrath, S., Goad, M.E., Tomkinson, K.N. et al. (2005). Regulation of muscle growth by multiple ligands signaling through activin type II receptors. Proc. Natl. Acad. Sci. U. S. A *102*, 18117-18122.
4. Kerkela, R., Karsikas, S., Szabo, Z., Serpi, R., Magga, J., Gao, E. et al. (2013). Activation of hypoxia response in endothelial cells contributes to ischemic cardioprotection. Molecular and cellular biology *33*, 3321-3329.
5. Kaikkonen, L., Magga, J., Ronkainen, V.P., Koivisto, E., Perjes, A., Chuprun, J.K. et al. (2014). p38alpha regulates SERCA2a function. Journal of molecular and cellular cardiology *67*, 86-93.
6. Dennler, S., Itoh, S., Vivien, D., ten Dijke, P., Huet, S., and Gauthier, J.M. (1998). Direct binding of Smad3 and Smad4 to critical TGF beta-inducible elements in the promoter of human plasminogen activator inhibitor-type 1 gene. The EMBO journal *17*, 3091-3100.
7. Korchynskyi, O., and ten Dijke, P. (2002). Identification and functional characterization of distinct critically important bone morphogenetic protein-specific response elements in the Id1 promoter. The Journal of biological chemistry *277*, 4883-4891.
8. Szabo, Z., Magga, J., Alakoski, T., Ulvila, J., Piuhola, J., Vainio, L. et al. (2014). Connective tissue growth factor inhibition attenuates left ventricular remodeling and dysfunction in pressure overload-induced heart failure. Hypertension *63*, 1235-1240.
9. Tolonen, A.M., Magga, J., Szabo, Z., Viitala, P., Gao, E., Moilanen, A.M. et al. (2014). Inhibition of Let-7 microRNA attenuates myocardial remodeling and improves cardiac function postinfarction in mice. Pharmacology research & perspectives *2*, e00056.
10. Rasanen, M., Degerman, J., Nissinen, T.A., Miinalainen, I., Kerkela, R., Siltanen, A. et al. (2016). VEGF-B gene therapy inhibits doxorubicin-induced cardiotoxicity by endothelial protection. Proceedings of the National Academy of Sciences of the United States of America *113*, 13144-13149.
