# Supplementary material for: Systemic Blockade of ACVR2B Ligands Protects Myocardium from Acute Ischemia-Reperfusion Injury
Source: Mol Ther. 2019 Jan 24;27(3):600–10. doi: 10.1016/j.ymthe.2019.01.013 (PMC6404100; doi:10.1016/j.ymthe.2019.01.013)
Supplement: Document S2. Article plus Supplemental Information [file mmc2.pdf]

Molecular Therapy
Original Article

AMERICAN SOCIETY of GENE & CELL THERAPY

Systemic Blockade of ACVR2B Ligands Protects Myocardium from Acute Ischemia-Reperfusion Injury

Johanna Magga,[1,2] Laura Vainio,[1] Teemu Kilpiö,[1] Juha J. Hulmi,[3,4] Saija Taponen,[1] Ruizhu Lin,[1,5] Markus Räsänen,[6] Zoltán Szabó,[1] Erhe Gao,[7] Lea Rahtu-Korpela,[1] Tarja Alakoski,[1] Johanna Ulvila,[1] Mika Laitinen,[8,9] Arja Pasternack,[4] Walter J. Koch,[7] Kari Alitalo,[6] Riikka Kivelä,[6] Olli Ritvos,[4] and Risto Kerkelä[1,5]

[1]Research Unit of Biomedicine, Department of Pharmacology and Toxicology, University of Oulu, 90220 Oulu, Finland; [2]Biocenter Oulu, University of Oulu, 90220 Oulu, Finland; [3]Neuromuscular Research Center, Biology of Physical Activity, Faculty of Sport and Health Sciences, University of Jyväskylä, 40014 Jyväskylä, Finland; [4]Department of Physiology, Faculty of Medicine, University of Helsinki, 00290 Helsinki, Finland; [5]Medical Research Center Oulu, Oulu University Hospital and University of Oulu, 90220 Oulu, Finland; [6]Wihuri Research Institute and Translational Cancer Biology Program, Faculty of Medicine, University of Helsinki, 00290 Helsinki, Finland; [7]Center for Translational Medicine, Temple University School of Medicine, Philadelphia, PA 19140, USA; [8]Department of Medicine, University of Helsinki, 00029 Helsinki, Finland; [9]Department of Medicine, Helsinki University Hospital, 00029 Helsinki, Finland

Activin A and myostatin, members of the transforming growth factor (TGF)-β superfamily of secreted factors, are potent negative regulators of muscle growth, but their contribution to myocardial ischemia-reperfusion (IR) injury is not known. The aim of this study was to investigate if activin 2B (ACVR2B) receptor ligands contribute to myocardial IR injury. Mice were treated with soluble ACVR2B decoy receptor (ACVR2B-Fc) and subjected to myocardial ischemia followed by reperfusion for 6 or 24 h. Systemic blockade of ACVR2B ligands by ACVR2B-Fc was protective against cardiac IR injury, as evidenced by reduced infarcted area, apoptosis, and autophagy and better preserved LV systolic function following IR. ACVR2B-Fc modified cardiac metabolism, LV mitochondrial respiration, as well as cardiac phenotype toward physiological hypertrophy. Similar to its protective role in IR injury *in vivo*, ACVR2B-Fc antagonized SMAD2 signaling and cell death in cardiomyocytes that were subjected to hypoxic stress. ACVR2B ligand myostatin was found to exacerbate hypoxic stress. In addition to acute cardioprotection in ischemia, ACVR2B-Fc provided beneficial effects on cardiac function in prolonged cardiac stress in cardiotoxicity model. By blocking myostatin, ACVR2B-Fc potentially reduces cardiomyocyte death and modifies cardiomyocyte metabolism for hypoxic conditions to protect the heart from IR injury.

INTRODUCTION

Development of the heart is guided by secreted morphogens including members of the transforming growth factor (TGF)-β superfamily.[1] In addition to their regulatory function in organogenesis, the TGF-β family of growth factors, including activins, bone morphogenetic proteins (BMPs), and growth differentiation factors (GDF), are known to regulate cardiac physiology and pathophysiology in the adult heart. These factors signal through type I and type II receptors, both of which are transmembrane serine and threonine kinases. Activins and GDFs bind to activin receptor IIA and B (ACVR2A and ACVR2B),[2,3] which in turn activate type I receptors such as activin receptor-like kinases (ALK) ALK4 and ALK5, activating downstream molecule SMAD2/3.[4,5] SMADSs regulate a number of myogenic genes, such as myoD, myogenin, and Myf5, that are involved in cellular hypertrophy, proliferation, or differentiation.[6] In addition to signaling via SMAD proteins, GDFs also signal through noncanonical pathways to regulate cardiomyocyte growth[7] by upregulation of atrophy-related atrogenes or autophagy genes, resulting in proteasome-dependent muscle protein degradation. Noncanonical ACVR2B pathways have also been shown to regulate MAP kinases.[5]

Activin A is upregulated in the heart after myocardial infarction (MI) or ischemia-reperfusion (IR) injury.[8,9] Serum levels of activin A increase in MI, and its expression levels correlate with creatinine kinase, as a measure of infarct size.[10] In an experimental model, cardiac myostatin (also known as GDF8) is upregulated immediately after MI.[11] Follistatin, an endogenous antagonist to myostatin and activin, has been shown to reduce IR injury in mice.[9] When utilizing myostatin-deficient mice, it was recently shown that the absence of myostatin improves cardiac function after MI.[12] In contrast to reports suggesting activin A as a culprit in IR injury, overexpression of activin A has also been shown to be protective against cardiomyocyte death, and its antagonism by Fstl3 exacerbates IR injury.[8] Myocardial stretch was recently shown to induce activin A in a genome-wide time series study of gene expression changes in stretched neonatal

Received 15 June 2018; accepted 16 January 2019;
https://doi.org/10.1016/j.ymthe.2019.01.013.

Correspondence: Johanna Magga, PhD (Pharm), Research Unit of Biomedicine, Department of Pharmacology and Toxicology, University of Oulu, Aapistie 5B, P.O. Box 5000, 90220 Oulu, Finland.
E-mail: johanna.magga@oulu.fi

This is an open access article under the CC BY license (http://creativecommons.org/licenses/by/4.0/).

cardiomyocytes.[13] Interestingly, activin A antagonists follistatin and Fstl3 were briefly upregulated after induction of stretch and substantially before activation of activin A.[13] It is not understood how activins and GDFs contribute to cardiomyocyte viability and function in myocardial ischemia.

Reperfusion by percutaneous catheter angioplasty is the main treatment for MI. While necessary to salvage ischemic myocardium, reperfusion itself may impair microvascular function and paradoxically trigger further injury.[14] Cardioprotection from reperfusion injury has been experimentally studied by local ischemic pre- and post-conditioning procedures as well as by remote ischemic preconditioning. This, achieved by short repetitive IR periods, is thought to activate pathways targeting mitochondria and thus reduce the formation of reactive oxygen species as well as inhibit the opening of mitochondrial permeability transition pore (mPTP).[14]

In addition, various growth factors and pharmacological agents have been explored for reducing reperfusion injury. Atrial natriuretic peptide (ANP), glucose-insulin-potassium therapy, glucagon like peptide-1 (GLP-1) analog exenatide or β-blocker metoprolol can potentially induce cardioprotection by inducing cGMP/PKG signaling, promoting glucose metabolism and utilization or reducing myocardial oxygen consumption, respectively.[15] Furthermore, agents targeting mitochondria by enhancing mitochondrial energetics or inhibiting mPTP opening have shown efficacy in experimental models of IR injury. However, many of these have not (yet) been shown to be cardioprotective in clinical studies.[16] Some of them have failed in clinical studies, possibly due to timing or dosing of the agent or heterologous patient population selected for the study.

Despite current invasive strategies to treat MI, novel cardioprotective agents are still needed to attenuate IR injury in order to prevent ischemia-induced heart failure and improve prognosis. In this study, we show that treating mice with ACVR2B-Fc decoy receptor protects the myocardium from IR injury. In earlier characterization, ACVR2B-Fc has been shown to bind endogenous ligands myostatin (GDF8), GDF11, and activin A with high affinity[17] and inhibit physiological responses of ACVR2B ligands.[18–20] Our results indicate that ACVR2B ligand myostatin activates SMAD2/3 and contributes to IR injury. This is salvageable by pharmacological inhibitor ACVR2B-Fc.

RESULTS

Systemic Blockade of ACVR2B Ligands Reduces Ischemic Injury and Restores Cardiac Function in an Experimental Model of Ischemia Reperfusion

To study the contribution of ACVR2B signaling to ischemic myocardial injury, we treated mice with a soluble decoy receptor of ACVR2B 24 h before ischemia ("ACVR2B-Fc pretreatment") to block the function of ACVR2B ligands and subjected the mice to transient IR by ligation of LAD. 30 min of ischemia followed by 6 h or 24 h of reperfusion resulted in left ventricular (LV) cell death and deteriorated cardiac function 24 h after IR. When analyzed with triphenyltetrazolium chloride (TTC) stain, ACVR2B-Fc reduced infarcted area in LV ($p < 0.01$; Figure 1A). This was accompanied by reduced, although not statistically significant, release of cardiac troponin I into plasma ($p = 0.09$; Figure 1B). Treatment with ACVR2B-Fc preserved cardiac function after IR as measured by echocardiography, but not if the treatment was initiated at reperfusion ("ACVR2B-Fc at reperfusion"). Ejection fraction was significantly better preserved with ACVR2B-Fc ($p < 0.01$; Figure 1C; see also Figure S1). In addition, fractional shortening and endocardial fractional area change were improved after ACVR2B-Fc treatment in comparison to vehicle-treated IR mice ($p < 0.01$; Table S2). Administration of ACVR2B-Fc reduced LV diameter ($p < 0.01$) and increased LV posterior wall thickness in IR mice ($p < 0.05$, Figures 1C and 1D; see also Table S2). This was associated with increased cardiomyocyte cross-sectional area ($p < 0.05$; Figure 1D), slightly increased LV mass (Figure 1C), and total heart weight (146 ± 18 mg in vehicle-treated and 158 ± 19 mg in ACVR2B-Fc-treated IR mice). A similar effect on cardiac hypertrophy was detected in healthy mice treated with sACVR2B as cardiomyocyte cross-sectional area was increased (Figure S2). The same mice also showed a non-significant increase in skeletal muscle hypertrophy (Figure S2). Cardiac hypertrophy was accompanied by transient phosphorylation and inactivation of GSK3β (Figure S2), which promotes LV hypertrophy and enhances resistance of cardiomyocytes to oxidative stress.[21]

Systemic Blockade of ACVR2B Ligands Reduces Ischemic Injury and Suppresses SMAD2, Apoptotic Stress-Induced Signaling Pathways, and Autophagy

To study the mechanisms of ACVR2B-Fc-mediated protection, we performed further analysis for apoptotic pathways. A TUNEL stain was performed 6 h after reperfusion and showed that ACVR2B-Fc reduced apoptosis in LV ($p < 0.05$; Figure 2A; Figure S2). This was accompanied by reduced expression of Bcl-2 family pro-apoptotic protein Bim ($p < 0.05$; Figure 2B). IR-induced activation of SMAD2 protein in the infarcted and peri-infarcted zone was inhibited by ACVR2B-Fc ($p < 0.001$; Figure 2C). In addition, ACVR2B-Fc reduced IR-induced phosphorylation of JNK ($p < 0.01$; Figure 2C) while having no effect on activation of ERK1/2 or p38. Furthermore, ACVR2B-Fc did not affect activation of phosphatidylinositol 3-kinase (PI3K)/Akt pathway (Figure 2C). ACVR2B signaling is known to promote autophagy, leading to protein degradation. Systemic blockade of ACVR2B ligands by ACVR2B-Fc led to reduction of autophagosomal LC3II form and decreased autophagosomal/cytoplasmic/LC3II/LC3I ratio ($p < 0.05$; Figure 2D) indicating reduced autophagy in the heart.

Since ACVR2B ligand GDF11 negatively regulates erythrocyte maturation[7] and activin A associates with inflammatory processes,[22] we additionally studied whether erythropoiesis, leukocyte infiltration, or inflammatory response after IR are affected by ACVR2B-Fc. Blood cell count performed 24 h after IR did not reveal any difference in red blood cell count between ACVR2B-Fc and vehicle-treated mice (Table S3), and leukocyte counts were also not affected. We then determined if ACVR2B-Fc affects granulocyte infiltration after IR. As analyzed from neutrophil stain, ACVR2B-Fc did not affect neutrophil infiltration into LV (Figure S3). In addition, ACVR2B-Fc did not

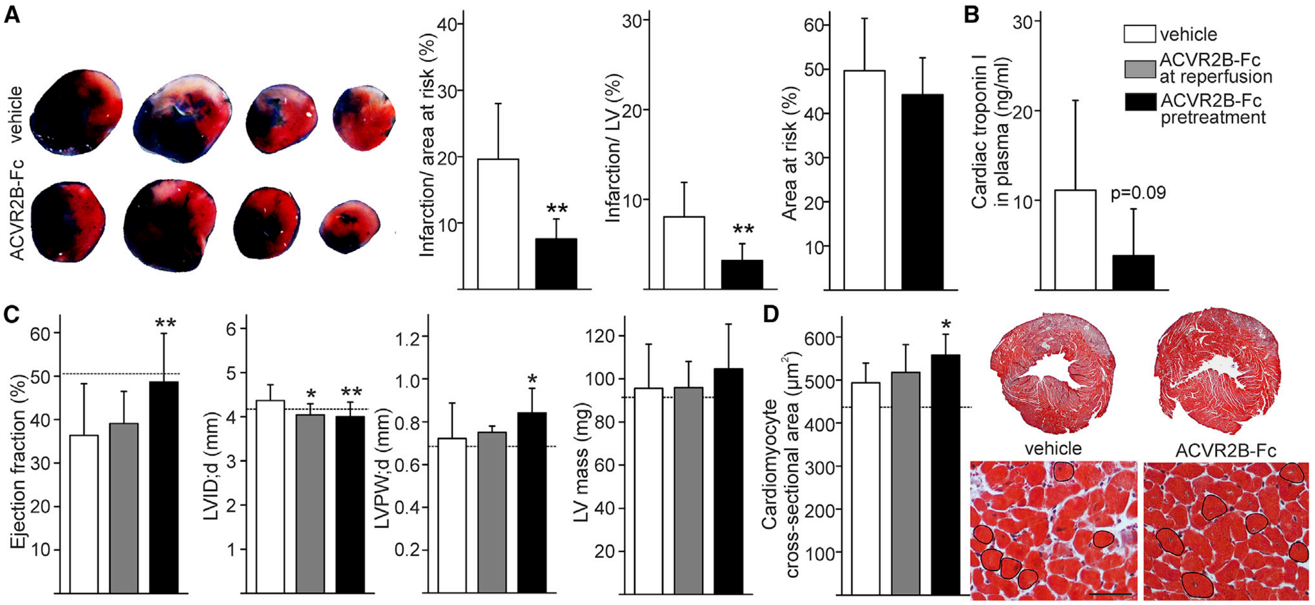

Figure 1. ACVR2B-Fc Reduces Ischemia-Reperfusion Injury and Restores Cardiac Function

Mice were treated with vehicle (white columns) or with a soluble decoy receptor ACVR2B-Fc at reperfusion (ACVR2B-Fc at reperfusion, gray columns) or 24 h prior to/at reperfusion (ACVR2B-Fc pretreatment, black columns). IR was achieved by transient ligation of left anterior descending (LAD) coronary artery for 30 min, followed by reperfusion for 24 h. (A) ACVR2B-Fc reduced infarcted area as determined with triphenyltetrazolium chloride (TTC stain). n = 7, 8. (B) ACVR2B-Fc reduced cardiac troponin I release into plasma (p = 0.09). n = 8, 9. (C) As determined with echocardiography, ACVR2B-Fc preserved cardiac function after IR observed as preserved ejection fraction. ACVR2B-Fc also reduced left ventricular diameter in diastole (LVID;d) and increased LV posterior wall thickness (LVPW;d) in IR mice with slightly increased total LV mass. Sham values are shown as a dotted line. n = 19, 9, 18. Full echo data in Table S2. (D) ACVR2B-Fc increased cardiomyocyte cross-sectional area. Masson trichrome stained heart section cut horizontally (above) and representative pictures of single cross-sectional cardiomyocytes circled for cardiomyocyte area analysis (below). Sham values are shown as a dotted line. Scale bar, 50 µm. n = 10, 9, 8. Data are presented as mean ± SD. $^{*}p < 0.05$, $^{**}p < 0.01$.

significantly reduce the expression of pro-inflammatory cytokines or chemokines in LV (Figure S3). Consequently, cardioprotection by ACVR2B-Fc was not explained by enhanced erythropoiesis or reduced inflammatory response.

ACVR2B-Fc Inhibits Myostatin-Mediated Activation of SMAD2/3 Pathway in Cardiomyocytes

First, we investigated whether ACVR2B-Fc induces cardioprotection directly to cardiomyocytes during hypoxia. Similar to cardioprotection *in vivo*, ACVR2B-Fc provided protection to adult cardiomyocytes from hypoxia-induced cell death ($p < 0.01$; Figure 3A). We then wanted to decipher which ACVR2B ligands could contribute to ischemic injury in adult cardiomyocytes. Myostatin and, to smaller extent, activin A, exacerbated ischemic injury in adult cardiomyocytes, while GDF11, activin B, or GDF15 had no effect (Figure 3B).

To study the ACVR2B-Fc-mediated SMAD signaling in cellular level, we transfected neonatal cardiomyocytes with CAGA-luc SMAD2/3 reporter or BRE-luc SMAD1/5/8 reporter and performed luciferase promoter assay to detect respective SMAD activity. To validate the model, we stimulated neonatal cardiomyocytes with factors expected to activate SMADs and to confirm that this signaling occurs in cardiomyocytes. As expected, myostatin, GDF11, activin A, activin B, and TGF-β induced SMAD2/3-dependent promoter activity (Figure 3C). GDF15, which signals via GFRAL receptor (not via ACVR2B receptor), was used here as a negative control and did not induce SMAD2/3 activity (Figure 3C). None of these ligands stimulated BRE-luc, which was used to assess SMAD1/5/8 activity, and which was activated by BMP4 (Figure S4). To confirm the efficacy of ACVR2B-Fc in reduction of SMAD activation, primary neonatal cardiomyocytes were subjected to hypoxia. As seen in Figure 3D, hypoxia induced SMAD2/3-dependent promoter activity in neonatal cardiomyocytes, while SMAD1/5/8 signaling was not activated. Administration of ACVR2B-Fc, which reduced SMAD2 signaling *in vivo*, reduced hypoxia-induced SMAD2/3-dependent promoter activity ($p < 0.01$; Figure 3D).

We then analyzed the time course of expression of ACVR2B ligands in the heart 6 h and 24 h after IR *in vivo*. qPCR analysis showed that levels of myostatin (*Mstn*) and activin A (*Inhba*) were upregulated in the infarct and/or peri-infarct zones while activin B (*Inhbb*) was not affected (Figure 3E; Figure S4). This was accompanied by corresponding changes in protein levels measured from LV by western blotting (Figure 3F). Similarly, immunostaining of LV sections showed increased activin A levels in the infarct and peri-infarct zones (Figure S4), localization of activin A in cardiomyocytes and, to a smaller extent, in endothelial cells. Interestingly, activin B was mainly detected in the infarct and peri-infarct zones in leukocytes, apparently

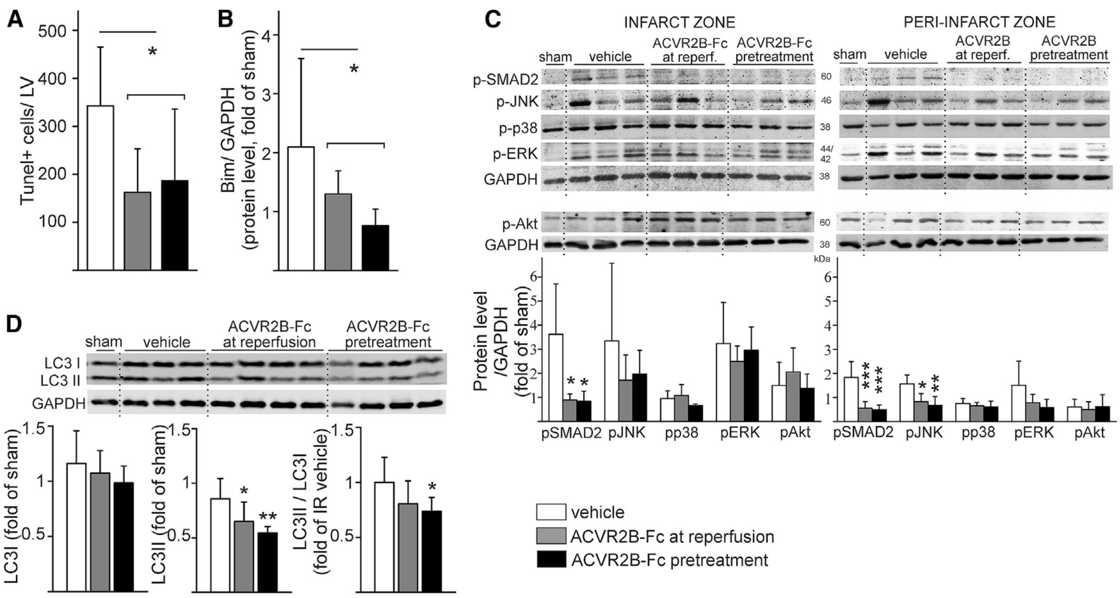

Figure 2. ACVR2B-Fc Reduces Activation of SMAD2, Apoptotic Stress-Induced Signaling Pathways, and Autophagy

Mice were treated with vehicle (white columns), with soluble decoy receptor ACVR2B-Fc only at reperfusion (gray columns), or with pretreatment of ACVR2B-Fc 24 h prior to/at reperfusion (black columns). IR was achieved by transient ligation of left anterior descending (LAD) coronary artery for 30 min, followed by reperfusion for 6 h. (A) ACVR2B-Fc reduced apoptosis. n = 5, 6, 6. (B) ACVR2B-Fc reduced expression of Bcl-2 family pro-apoptotic protein Bim. n = 5, 6, 6. (C) ACVR2B-Fc inhibited IR-induced activation of SMAD2 protein in infarcted and peri-infarcted zone. ACVR2B-Fc also reduced IR-induced phosphorylation of JNK but had no effect on activation of p38, ERK1/2, or Akt. n = 5, 6, 6. (D) ACVR2B-Fc reduced autophagosomal lipidated LC3II form and decreased autophagosomal/cytoplasmic/LC3II/LC3I ratio in LV as analyzed 24 h after reperfusion. n = 7, 8, 8. Data are presented as mean ± SD. *p < 0.05, **p < 0.01, ***p < 0.001.

neutrophils, and was only found in cardiomyocytes 24 h after IR and to a smaller extent than observed in the leukocytes. Myostatin levels were already high at basal levels in sham hearts, and there was a minor increase in myostatin levels in infarct and peri-infarct zones at 6 h and 24 h after IR; myostatin expression was only localized to cardiomyocytes (Figure S4).

Different from other ACVR2B ligands, *Gdf11* was downregulated in the early phase after IR but upregulated at 24 h (Figure 3G). No change was observed in *Tgfb* expression following IR (Figure 3G). When determining the expression levels of activin receptors, both *Acvr2B* and *Acvr2A* receptors were downregulated in the peri-infarct zone at 6 h after IR (Figure 3G). After 24 h, *Acvr2B* downregulation sustained, while *Acvr2A* expression was elevated back to basal level. Expression of *Bmpr2*, the receptor for BMP ligands, was not affected (1 ± 0.17 in sham versus 0.93 ± 0.29 at IR 24 h). Finally, as confirmed in neonatal cardiomyocytes, myostatin and activin A were upregulated after hypoxia, while the expression of activin B was not changed (Figure 3H). In summary, our data suggest myostatin and activin A are elevated shortly after hypoxia and they activate ACVR2B in cardiomyocytes, leading to increased SMAD2/3 activity. Myostatin exacerbated hypoxic injury, which was associated with increased cardiomyocyte death. Similar to cardioprotective effects of ACVR2B-Fc observed in IR model *in vivo*, ACVR2B-Fc directly protected cardiomyocytes *in vitro*.

Systemic Blockade of ACVR2B Ligands Optimizes Cardiac Metabolism to Hypoxic Conditions in the IR Model

We next studied the effect of ACVR2B-Fc on cardiac metabolism following cardiac IR injury. We found that administration of ACVR2B-Fc upregulated the expression of peroxisome proliferator-activated receptor gamma coactivator 1 (*Ppargc1a*) isoforms Pgc1α1 and Pgc1α4 ($p < 0.05$; Figure 4A), which are central regulators of mitochondrial energy production. ACVR2B-Fc did not affect the gene expression of oxidative phosphorylation enzyme cytochrome C (*Cycs*) or glycolytic enzymes *Pgam1* or *Gapdh* (Figure 4A). However, improvement of energy metabolism by ACVR2B-Fc was associated with an increased expression of glycolytic phosphofructokinase enzyme *Pfkm* and upregulation of insulin-regulated glucose transporter *Glut4* ($p < 0.05$; Figure 4A), suggesting an increased glucose uptake and glycolysis. ACVR2B-Fc increased phosphorylation of acetyl-CoA carboxylase, reducing its enzymatic activity in the fatty acid synthesis pathway in healthy hearts (Figure S4). However, ACVR2B-Fc did not reduce fatty acid synthesis in IR hearts (Figure S4).

Administration of ACVR2B-Fc had no effect on acute IR-injury-induced increase in expression of atrial or B-type natriuretic peptides ANP (*Nppa*) (IR vehicle 3.2 ± 1.4 versus IR ACVR2B-Fc pretreatment 2.6 ± 1.1, p = 0.52) or BNP (*Nppb*) (IR vehicle 1.9 ± 0.8 versus IR ACVR2B-Fc pretreatment 1.5 ± 0.6, p = 0.44) but affected the

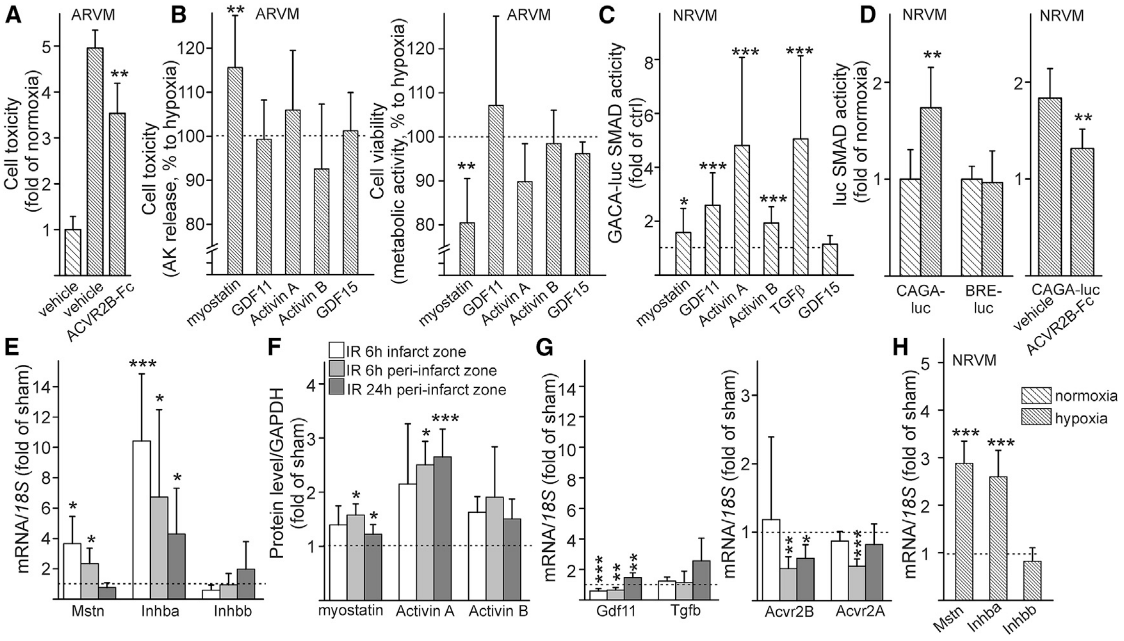

Figure 3. ACVR2B-Fc Protects Cardiomyocytes in Hypoxic Conditions and Inhibits Myostatin-Activated SMAD2/3 Pathway in Cardiomyocytes

(A) ACVR2B-Fc protects adult rat ventricular cardiomyocytes (ARVM) from hypoxia-induced cell death as measured with adenylate kinase release. n = 6. (B) ACVR2B ligand myostatin reduces cell survival in hypoxia. Cell toxicity was assessed with adenylate kinase release, and cell viability was determined with resazurin assay measuring cell metabolism. The hypoxia value is shown as a dotted line. n = 6. (C) Neonatal rat ventricular cardiomyocytes (NRVM) were transfected with CAGA-luc SMAD2/3 reporter, and subsequently luciferase reporter activity was analyzed after treatment with ACVR2B ligands, TGF-β, or the GFRAL receptor ligand GDF15. n = 12. (D) Hypoxia induced SMAD2/3-dependent promoter activity in NRVM while SMAD1/5/8 signaling was not activated. ACVR2B-Fc reduced hypoxia-induced SMAD2/3-dependent promoter activity in NRVM. n = 6. (E) Expression of ACVR2B ligands myostatin (*Mstn*), activin A (*Inhba*), and activin B (*Inhbb*) in infarct and peri-infarct zone in LV 6 and 24 h after IR, analyzed by qPCR. The sham value is shown as a dotted line. n = 5–6 (6 h IR); n = 8–9 (24 h IR). (F) The levels of ACVR2B ligands in LV, analyzed by western blotting. n = 5 (6 h IR); n = 9 (24 h IR). (G) Expression of ACVR2B ligand *Gdf11* and *Tgfb*, as a control, and ACVR2B receptors *Acvr2B* and *Acvr2A* in LV 6 and 24 h after IR, analyzed by qPCR. n = 5–6 (6 h IR); n = 8–9 (24 h IR). (H) As confirmed in NRVM, myostatin and activin A were upregulated after hypoxia. The normoxia value is shown as dotted line. n = 6. Data are presented as mean ± SD. *p < 0.05, **p < 0.01, ***p < 0.001.

composition of cardiomyocyte myosin fibers. ACVR2B-Fc slightly, although not significantly, decreased expression of myosin heavy chain (MHC)-β (*Myh7*) slow twitch isoform while increasing expression of MHC-α (*Myh6*) fast twitch myosin fibers (p < 0.01; Figure 4B). This was accompanied with increased expression of *Cited4* (p < 0.05; Figure 4B), a transcription factor involved in physiological hypertrophy.[23,24]

To confirm the effect of ACVR2B-Fc on cardiomyocyte metabolism, we performed a bioenergetic assay in cardiomyocytes *in vitro*. Adult LV cardiomyocytes obtained from mice treated with ACVR2B-Fc for 48 h showed reduced metabolic activity with attenuated oxygen consumption when subjected to hypoxic conditions *in vitro* (Figure 4C). Cardiomyocytes of ACVR2B-Fc-treated mice showed both reduced maximal respiration and reduced spare respiratory capacity compared to cardiomyocytes from vehicle-treated mice (Figure 4D). We did not detect pronounced induction of glycolysis (Figure 4E), and upregulation of mitochondrial glycolytic enzymes detected by qPCR may thus represent a compensatory increase of metabolic enzymes after myocardial hibernation.

Systemic Blockade of ACVR2B Ligands during Prolonged Cardiac Stress Improves LV Function

To determine the long-term effects of ACVR2B-Fc-induced metabolic changes on cardiac function, we measured mitochondrial respiration in LV *ex vivo*. We treated mice with anthracycline antitumor agent doxorubicin, which induces cardiotoxicity by increased oxidative stress, alterations in ion homeostasis, inhibition of protein synthesis, and eventually, mitochondrial failure.[25] To study if ACVR2B-Fc could protect the heart from doxorubicin-induced deterioration of cardiac metabolic function, we measured oxidative function from LV utilizing a high-resolution respirometer. We found that ACVR2B-Fc treatment improved cardiac respiration in doxorubin-stressed hearts (Figure 5A). ACVR2B-Fc also slightly, but significantly, improved citrate synthase activity (Figure 5B). This result may be independent of mitochondrial number, as doxorubicin or ACVR2B-Fc had no significant effect on the mitochondrial respiratory chain (OXPHOS and cytochrome C) protein contents (data not shown). Furthermore, utilizing echocardiography, we assessed the effect of ACVR2B-Fc treatment on cardiac function after cumulative doxorubicin-induced toxicity. We found that treatment of mice with ACVR2B-Fc inhibited the doxorubicin-induced deterioration of cardiac systolic function

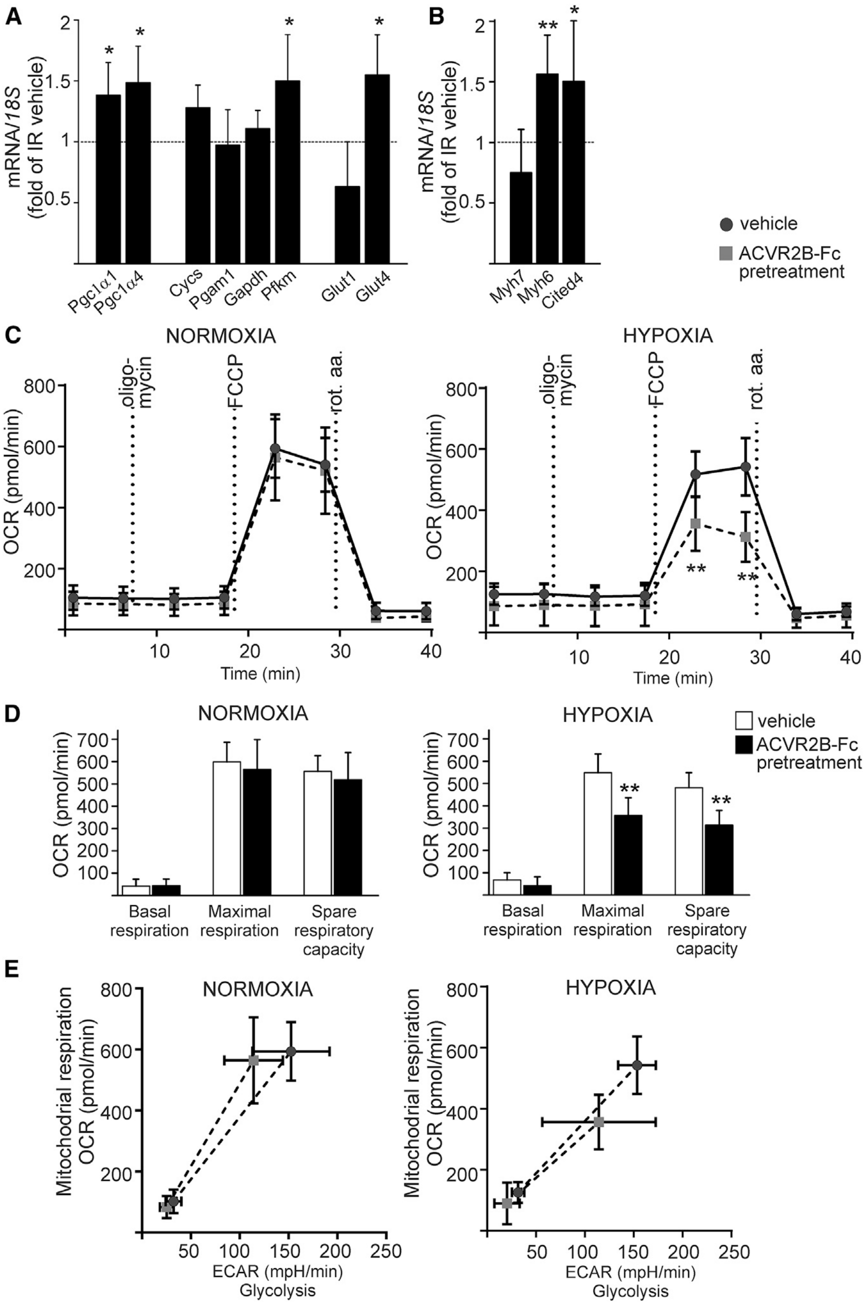

Figure 4. ACVR2B-Fc Optimizes Metabolism to Hypoxic Conditions in IR

Expression of genes were analyzed with qPCR 24 h after IR from the peri-infarct zone. (A) ACVR2B-Fc upregulated expression of peroxisome proliferator-activated receptor gamma coactivator 1 isoforms PGC1α1 and PGC1α4 and did not affect the gene expression of oxidative phosphorylation enzyme cytochrome C (*Cycs*), but it increased expression of glycolytic phosphofructokinase enzyme *Pfkm* and insulin-regulated glucose uptake transporter *Glut4*. Vehicle-treated IR mouse values are shown as dotted line. n = 9, 8. (B) ACVR2B-Fc upregulated expression of MHC-α (*Myh6*) fast twitch myosin fibers and *Cited4*, a transcription factor involved in physiological hypertrophy. Vehicle-treated IR mouse values are shown as a dotted line. n = 9, 8. Data are presented as mean ± SD. $^{*}p < 0.05$, $^{**}p < 0.01$. (C) ACVR2B-Fc reduced metabolism in adult mouse cardiomyocytes after 4 h hypoxia followed by 1 h reperfusion as determined by oxygen consumption rate (OCR) with bioenergetic mito stress assay. (D) The values for basal and maximal respiration and spare respiratory capacity were calculated from OCR graphs. n = 13, 11 (normoxia); n = 6, 5 (hypoxia). (E) Bioenergetic phenotype profile visualized from mito stress assay data. Basal phenotype from the beginning of the experiment versus stressed phenotype (after oligomycin and FCCP injections) in cells subjected to normoxia or hypoxia. n = 13, 11 (normoxia); n = 6, 5 (hypoxia).

(Figure 5C; see also Table S4). qPCR analysis of cardiac samples showed that administration of ACVR2B-Fc reduced doxorubicin-induced upregulation of natriuretic peptides ANP (*Nppa*) and BNP (*Nppb*) (Figure 5D), indicators of pathological cardiac remodeling.[26]

DISCUSSION

ACVR2B-Fc Contributes to Ischemia Protection by Regulation of Myostatin Signaling in Cardiomyocytes

Repetitive ischemic preconditioning downregulates expression of myostatin in both ischemic and remote myocardium. This suggests downregulation of myostatin is associated with preconditioning-induced cardioprotection in reperfusion injury.[27] Here, we demonstrate that systemic blockade of ACVR2B ligands protects the heart from IR injury by reducing hypoxia-activated myostatin/SMAD2 signaling. Treatment with ACVR2B-Fc reduces apoptosis and optimizes cardiomyocyte energy metabolism in hypoxic conditions.

In addition to BMPs,[28,29] myostatin and activin A are potent regulators of muscle growth.[30] Myostatin is predominantly expressed in the skeletal muscle but also to lower extent in the heart. Myostatin is an important negative regulator of muscle growth, as evidenced by massive skeletal muscle hypertrophy in myostatin knockout mice.[31] Constitutive myostatin knockout induces myofiber hypertrophy and hyperplasia during development and[32] results in downregulation of genes encoding slow isoforms of contractile proteins and genes encoding proteins involved in energy metabolism.[33] We also detected expression of these genes modified in our ACVR2B-Fc-treated IR hearts. In contrast, earlier studies with post-developmental reduction of myostatin expression by Cre-lox recombination or by ACVR2B-Fc resulted in hypertrophy, but not to the downregulation of genes encoding slow isoforms of skeletal muscle

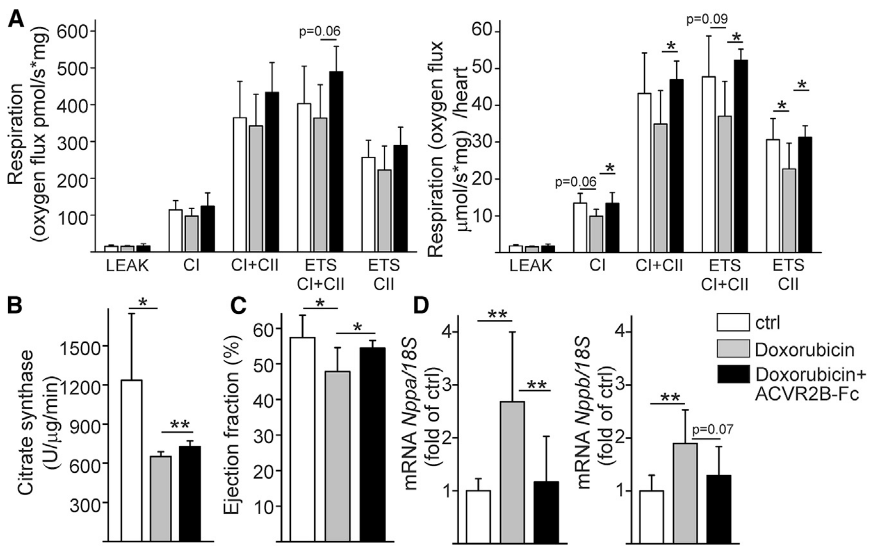

Figure 5. ACVR2B-Fc Improves LV Function under Prolonged Cardiac Stress

(A) Mice were treated with anthracycline doxorubicin for 2-week period, which induces cardiotoxicity. Mice were treated simultaneously with vehicle or ACVR2B-Fc for 2 weeks and LV tissue was analyzed with high-resolution respirometer. ACVR2B-Fc improved cardiac respiration in doxorubicin-stressed hearts. Right panel represents respiration capacity for the whole heart. CI, complex I; CII, complex II; ETS, electron transfer system (maximal uncoupled respiration). n = 7. (B) ACVR2B-Fc also slightly improved citrate synthase activity. n = 7. (C) As analyzed with echocardiography, ACVR2B-Fc preserved cardiac systolic function in cumulative doxorubicin-induced toxicity at 4 weeks. n = 9, 6, 9. Full echo data in Table S4. (D) As analyzed with qPCR from LV at 4 weeks, ACVR2B-Fc reduced doxorubicin-induced upregulation of natriuretic peptides ANP (*Nppa*) and BNP (*Nppb*). n = 9, 7, 8. Data are presented as mean ± SD. *$p < 0.05$, **$p < 0.01$.

contractile proteins or genes encoding proteins involved in energy metabolism.[34,35]

Activins mainly reside in gonadal tissue and regulate reproduction by stimulating FSH release from the pituitary gland. In addition, activin A is expressed in the skeletal muscle and the heart and regulates essential biological functions, such as cell proliferation and differentiation, immune response, and angiogenesis.[5] Similarly to myostatin, activin A negatively and prominently regulates muscle growth.[9,30] In a recent study in zebrafish, activin A and myostatin were shown to have opposite effects on cardiac repair after cryoinjury: activin A led to accelerated recovery, whereas myostatin hindered the regeneration process. Furthermore, myostatin was suggested to bind predominantly to ACVR2B and activate SMAD2, while activin A was suggested to bind to ACVR2A and promote activation of SMAD3.[36] This is in line with our findings showing that myostatin exacerbates ischemic injury and ACVR2B-Fc blocks the activation of myostatin-activated SMAD2. However, our data does not allow us to determine to which extent the ACVR2B-Fc-mediated cardioprotection stems from inhibition of canonical SMAD2 pathway or inhibition of non-canonical JNK pathway. SMAD2/3 controls genes regulating hypertrophy and atrophy, even apoptosis. JNK can also regulate hypertrophy, metabolism, and mitochondria-mediated cell death in response to oxidative stress.[37] JNK can even interact with SMAD2 pathway in regulation of muscle remodeling.[38] Apparently, the anti-apoptotic effect of ACVR2B-Fc is mediated by inhibition of JNK activity but occurs in cross-talk with other signaling pathways, including SMAD2.

BMPs have been shown to contribute to ischemic injury,[39] but we did not detect immediate BMP activation in cardiomyocytes in response to hypoxia by measuring BRE-luc promoter activity. Fstl1, inhibiting BMPs and their signaling via the SMAD1/5/8 pathway, has been shown to reduce IR injury by reduction of apoptosis and inflammatory response.[40,41] In a recent study, Fstl1 was also shown to alter energy substrate metabolism and increase oxidative respiration in the heart.[42] It remains to be shown how BMP and GDF pathways, via SMAD1/5/8 and SMAD2/3, respectively, or via noncanonical pathways, overlap in their contribution to IR injury.

GDF11, although a close homolog to myostatin, has versatile effects partly divergent from myostatin. Myostatin predominantly affects muscle mass, while the ablation of GDF11 results in defects in skeletal patterning during embryogenesis, resulting in perinatal lethality.[43,44] Unlike myostatin, GDF11 also participates in erythropoiesis in adult. GDF11 was formerly considered as a rejuvenation factor, and restoration of levels of GDF11 were shown to provide protection from age-related pathological cardiac hypertrophy.[45] In subsequent studies, aging was not associated with a decrease in circulating GDF11 levels,[46] and elevation of GDF11 did not provide therapeutic effect for cardiac hypertrophy.[47] In skeletal muscle, GDF11 expression was even shown to increase during aging and inhibit muscle regeneration, similar to inhibitory effects of myostatin.[48] Inducing supraphysiological levels of GDF11 led to both skeletal and cardiac muscle atrophy, while myostatin reduced only skeletal muscle growth.[49] We detected upregulation of cardiac myostatin and activin A expression immediately after ischemia, while expression of GDF11 was upregulated at a later phase. This is of interest since GDF11 was recently shown to reduce cardiac remodeling after IR injury.[50]

Our results thus confirm that myostatin and its close homolog GDF11 are differently expressed in the heart in response to ischemia and indicate that they may participate in different processes. Furthermore, GDF11 did not affect cardiomyocyte survival in acute hypoxia, while administration of myostatin exacerbated cardiomyocyte death. This suggests that, blocking the effects of GDF11 as a bystander, ACVR2B-Fc does not exacerbate myocardial ischemic injury. We cannot fully rule out that additionally, blockade of GDF11 could partly contribute to benefits of ACVR2B-Fc in IR. GDF11 is known to negatively affect erythrocyte maturation,[7] and its blockade by

ACVR2B-Fc increases extramedullary hematopoiesis leading to splenomegaly.[30] When performing erythrocyte count 24 h post-MI, we found short-term treatment with ACVR2B-Fc did not increase the number of erythrocytes, excluding increased red blood cell availability as a possible benefit during IR.

ACVR2B-Fc Contributes to Ischemia Protection by Regulation of Catabolic Pathways and Hypertrophy

In the present study, administration of ACVR2B-Fc rapidly induced cardiomyocyte growth. This was not accompanied by downregulation of atrogenes or induction of Akt, although GSK3β phosphorylation was transiently increased. In a previous study using our ACVR2B-Fc, blocking ACVR2B ligands did not increase protein synthesis in the healthy heart as it does in the skeletal muscle.[20] Here, administration of ACVR2B-Fc reduced lipidated LC3, a marker of autophagy, in the heart, suggesting that decreased autophagy may be a mechanism of increased cardiomyocyte size by ACVR2B-Fc. The administration of ACVR2B-Fc has also reduced LC3 lipidation in skeletal muscle,[19] which probably occurs through blocking of myostatin, as myostatin can induce autophagy.[51] It is also possible that ACVR2B-Fc-induced higher glucose concentration or uptake contributed to increased hypertrophy, especially under apparent sympathetic stimulus.[52] CITED4 overexpression in cardiomyocytes was recently shown to be sufficient for the induction of cardiac hypertrophy and reduction of autophagy, reduced adverse cardiac remodeling, and reduced fibrosis after ischemic injury.[24] Interestingly, we detected rapid induction of cardiomyocyte hypertrophy by ACVR2B-Fc in IR hearts, which was accompanied by upregulation of CITED4.

ACVR2B-Fc Contributes to Ischemia Protection by Regulation of Cardiomyocyte Metabolism

Approaches to alleviate IR injury aim at pharmaceutical compounds that reduce fatty acid uptake into mitochondria, inhibit mitochondrial fatty acid oxidation, or increase glucose oxidation.[53] Blocking myostatin signaling in the heart by genetically inactivating myostatin from cardiomyocytes results in enhanced glycolysis, augmented glycogen storage, and cardiac hypertrophy in adult mice.[54] In that study, however, myostatin deletion led to LV dilatation, impaired cardiac function, and increased mortality in otherwise healthy mice.[54] Authors of the study showed this metabolic switch leading to cardiac hypertrophy to be mediated by AMPK activation. Similarly, we found AMPK target acetyl CoA carboxylase to be phosphorylated by myostatin inhibition by ACVR2B-Fc in healthy hearts. Phosphorylation of acetyl CoA carboxylase downregulates fatty acid synthesis.[55] However, we did not detect an increase in acetyl CoA carboxylase phosphorylation in IR hearts, suggesting that in contrast to healthy hearts, ACVR2B-Fc did not affect fatty acid synthesis in ischemic hearts.

Upregulation of PGC1α results in increased mitochondrial biogenesis and oxidative phosphorylation.[56] PGC1α4 isoform that results from alternative promoter usage and splicing of the primary PGC1α transcript induces robust skeletal muscle hypertrophy without producing a metabolic phenotype similar to what PGC1α1 isoform produces.[57] In our study, upregulation of PGC1α1 and PGC1α4 isoforms by ACVR2B-Fc (together with inhibition of GSK3β) at least partly explain the increased hypertrophy but may represent compensatory response to metabolic changes in the heart. Inhibition of myostatin signaling has also been shown to have beneficial metabolic effects in obesity and diabetes, including enhanced glucose tolerance, improved brown adipogenesis, and reduced fat mass.[7,58]

Our data shows that ACVR2B-Fc treatment reduced metabolic activity in adult mouse cardiomyocytes after IR, reducing oxygen consumption. This resembles the phenomenon known as myocardial hibernation, in which the heart downregulates metabolism in order to adapt to ischemic conditions.[59] At reperfusion, this may reduce the burst of reactive oxygen species and calcium overload in cardiomyocytes, and this approach has actually been suggested as a possible therapy for reperfusion injury. Basheer et al.[60] recently demonstrated a similar cardioprotective phenotype in ischemic injury with pretreatment of mice with adenoviral infection of a mitochondrial targeting factor. The approach mimicked the cardioprotective effect of ischemic preconditioning by inducing metabolic quiescence and limiting production of damaging levels of reactive oxygen species in the mitochondria. Our results suggest ACVR2B-Fc may promote entering of the cardiomyocytes to an adaptive hibernating state that reduces energy substrate utilization and oxygen demand to match the oxygen availability in IR.

Finally, we determined whether the acute cardioprotective effect and metabolic changes induced by ACVR2B-Fc could provide beneficial effects on cardiac function after prolonged cardiac stress. Efficacy of ACVR2B-Fc on mitochondria function was determined in a cardiotoxicity model, as doxorubicin is known to alter cardiac metabolism.[25] Besides the disruption of mitochondrial oxidative respiration, which includes inhibition of complex I activity, many other proteins in metabolic pathways, such as mitochondrial creatine kinases, are affected by doxorubicin.[25] We found that treatment of mice with ACVR2B-Fc improved mitochondrial function and prevented the deterioration of cardiac systolic function in doxorubicin-treated mice. This suggests that, in addition to its acute prosurvival and metabolic effects in IR injury, ACVR2B-Fc has more broad beneficial effects on the heart.

Cardiac Preconditioning by ACVR2B-Fc Is Required for Full Cardioprotection

Activin A and mature myostatin reside in a latent complex extracellularly, ready to bind ACVR2B when released from their binding peptides. Activin and myostatin activities are endogenously inhibited by follistatin or follistatin-like proteins or, in case of myostatin, by GDF-associated serum proteins that bind to ligands and neutralize their effects. Activins are also inhibited by inhibins that interfere ACVR2A/ACVR2B binding to type I receptors and activation of intracellular signal transduction cascades such as SMAD2/3.

We chose to study ACVR2B-Fc as a therapeutical approach due to its benefits in hypertrophic muscle growth and its broader ligand

specificity. ACVR2B-Fc blocks signaling of myostatin, its close homolog GDF11, as well as activin A, activin B, and BMP10.[2] Inhibition of myostatin signaling by ACVR2B-Fc has been studied in muscle-wasting conditions. Myostatin signaling blockade has been achieved by specific antibodies,[61] by antibodies targeted to ACVR2,[62] ligand traps including ACVR2B-Fc,[18–20,63,64] or by overexpression of natural inhibitors such as follistatin.[40,42] However, myostatin and other TGF-β family members, especially GDF11 and activin A, share a high degree of similarity in receptor recognition sites thus lacking target specificity. Recently, human monoclonal antibodies to pro-myostatin and pro-GDF11 were developed.[65] These inhibit their targets by blocking growth factor release from the prodomain and work with higher specificity, since pro-domains are much less conserved than mature domains. It remains to be shown whether pro-domain targeting approach, which reduced glucocorticoid-induced muscle wasting,[65] could also work in protection from IR injury and whether it offers extra benefits when inhibiting myostatin specifically.

To conclude, the changes described in this study, which improve cardiomyocyte response to hypoxia, predominantly require ACVR2B-Fc to be administered prior to IR. Although administration of ACVR2B-Fc at reperfusion inhibits canonical SMAD2/3 and non-canonical pathways, this is sufficient to only partially inhibit pathological pathways. Therefore, transcriptional modification toward metabolically optimized cardiac function is needed to achieve full cardioprotective effect of ACVR2B-Fc. Our findings resemble ischemic preconditioning, which is known to protect the heart from IR injury and is associated with priming of mitochondria into a metabolically altered stress-resistant state. According to our results, systemic blockade of ACVR2B ligands was sufficient to promote protection from IR in the heart. In addition to ACVR2B, myostatin and activin A also signal through ACVR2A receptors. In a recent study, dual blockade of ACVR2A/ACVR2B by bimagrumab was shown to be required for full anabolic response in skeletal muscle.[3] It remains to be shown whether dual blockade of ACVR2 receptors could offer extra benefit in treatment of IR injury.

MATERIALS AND METHODS

An expanded methods section is provided in the Supplemental Information. Experimental protocols were approved by the Animal Use and Care Committee of the University of Oulu and the national Animal Experiment Board of Finland. 8- to 10-week-old male C57BL/6J mice were anaesthetized with isoflurane and subjected to IR by ligation of the left anterior descending coronary artery (LAD) for 30 min, after which the slip knot was released, allowing reperfusion of the ischemic myocardium for 6 or 24 h, as previously described.[66] The experimental timeline is depicted in Figure S1. ACVR2B-Fc recombinant fusion protein[18] was administered as 10 mg/kg subcutaneously (s.c.) 24 h prior to IR and at reperfusion (termed "ACVR2B-Fc pretreatment") or only at reperfusion (termed "ACVR2B-Fc at reperfusion"). Data are expressed as mean ± SD. $^{*}p < 0.05$, $^{**}p < 0.01$ and $^{***}p < 0.001$.

SUPPLEMENTAL INFORMATION

Supplemental Information includes four figures and four tables, and Supplemental Materials and Methods and can be found with this article online at https://doi.org/10.1016/j.ymthe.2019.01.013.

AUTHOR CONTRIBUTIONS

J.M. participated in experimental studies, analyzed the results, and wrote the manuscript. L.V., T.K., and Z.S. performed experimental studies and participated in analysis of the results. R.L. and L.R.-K. performed histological analysis. J.J.H., M.R., K.A., and R. Kivelä designed, performed, and/or analyzed experimental studies on cardiotoxicity. E.G., W.J.K., and R. Kerkelä designed and/or performed experimental IR studies. S.T., T.A., and J.U. performed *in vitro* studies. M.L., A.P., and O.R. designed and produced the pharmacological agent and participated in design of the study. J.J.H., L.V., R. Kivelä, and R. Kerkelä critically revised the manuscript. All authors have read and approved final manuscript.

CONFLICTS OF INTEREST

The authors declare no competing interests.

ACKNOWLEDGMENTS

We thank Marja Arbelius, Sirpa Rutanen, and Kirsi Salo (University of Oulu) for excellent technical assistance. We also acknowledge Joni Degerman, Maria Arrano de Kivikko, and Nada Bechara-Hirvonen (Wihuri Research Institute, University of Helsinki) and Tuuli Nissinen (University of Jyväskylä) for excellent technical assistance. This work was supported by research funding from Academy of Finland grants 268505 (J.M.), 275922 (J.J.H.), and 297094 (R. Kerkelä); the People Programme (Marie Curie Actions) of the European Union's Seventh Framework Programme FP7/2007-2013 under REA grant 317250 (M.R.); the Emil Aaltonen Foundation (T.K.); and the Finnish Foundation for Cardiovascular Research (J.M., L.V., T.K., Z.S., R. Kerkelä).

REFERENCES

1. Noseda, M., Peterkin, T., Simões, F.C., Patient, R., and Schneider, M.D. (2011). Cardiopoietic factors: extracellular signals for cardiac lineage commitment. Circ. Res. *108*, 129–152.

2. Souza, T.A., Chen, X., Guo, Y., Sava, P., Zhang, J., Hill, J.J., Yaworsky, P.J., and Qiu, Y. (2008). Proteomic identification and functional validation of activins and bone morphogenetic protein 11 as candidate novel muscle mass regulators. Mol. Endocrinol. *22*, 2689–2702.

3. Morvan, F., Rondeau, J.M., Zou, C., Minetti, G., Scheufler, C., Scharenberg, M., Jacobi, C., Brebbia, P., Ritter, V., Toussaint, G., et al. (2017). Blockade of activin type II receptors with a dual anti-ActRIIA/IIB antibody is critical to promote maximal skeletal muscle hypertrophy. Proc. Natl. Acad. Sci. USA *114*, 12448–12453.

4. Shi, Y., and Massagué, J. (2003). Mechanisms of TGF-beta signaling from cell membrane to the nucleus. Cell *113*, 685–700.

5. Han, H.Q., Zhou, X., Mitch, W.E., and Goldberg, A.L. (2013). Myostatin/activin pathway antagonism: molecular basis and therapeutic potential. Int. J. Biochem. Cell Biol. *45*, 2333–2347.

6. Dschietzig, T.B. (2014). Myostatin - From the Mighty Mouse to cardiovascular disease and cachexia. Clinica Chim Acta *433*, 216–224.

7. Rochette, L., Zeller, M., Cottin, Y., and Vergely, C. (2015). Growth and differentiation factor 11 (GDF11): Functions in the regulation of erythropoiesis and cardiac regeneration. Pharmacol. Ther. *156*, 26–33.

8. Oshima, Y., Ouchi, N., Shimano, M., Pimentel, D.R., Papanicolaou, K.N., Panse, K.D., Tsuchida, K., Lara-Pezzi, E., Lee, S.J., and Walsh, K. (2009). Activin A and follistatin-like 3 determine the susceptibility of heart to ischemic injury. Circulation *120*, 1606–1615.

9. Chen, Y., Rothnie, C., Spring, D., Verrier, E., Venardos, K., Kaye, D., Phillips, D.J., Hedger, M.P., and Smith, J.A. (2014). Regulation and actions of activin A and follistatin in myocardial ischaemia-reperfusion injury. Cytokine *69*, 255–262.

10. Miyoshi, T., Hirohata, S., Uesugi, T., Hirota, M., Ohnishi, H., Nogami, K., Hatanaka, K., Ogawa, H., Usui, S., and Kusachi, S. (2009). Relationship between activin A level and infarct size in patients with acute myocardial infarction undergoing successful primary coronary intervention. Clin. Chim. Acta *401*, 3–7.

11. Castillero, E., Akashi, H., Wang, C., Najjar, M., Ji, R., Kennel, P.J., Sweeney, H.L., Schulze, P.C., and George, I. (2015). Cardiac myostatin upregulation occurs immediately after myocardial ischemia and is involved in skeletal muscle activation of atrophy. Biochem. Biophys. Res. Commun. *457*, 106–111.

12. Lim, S., McMahon, C.D., Matthews, K.G., Devlin, G.P., Elston, M.S., and Conaglen, J.V. (2018). Absence of Myostatin Improves Cardiac Function Following Myocardial Infarction. Heart Lung Circ. *27*, 693–701.

13. Rysä, J., Tokola, H., and Ruskoaho, H. (2018). Mechanical stretch induced transcriptomic profiles in cardiac myocytes. Sci. Rep. *8*, 4733.

14. Heusch, G., and Gersh, B.J. (2017). The pathophysiology of acute myocardial infarction and strategies of protection beyond reperfusion: a continual challenge. Eur. Heart J. *38*, 774–784.

15. Ferdinandy, P., Hausenloy, D.J., Heusch, G., Baxter, G.F., and Schulz, R. (2014). Interaction of risk factors, comorbidities, and comedications with ischemia/reperfusion injury and cardioprotection by preconditioning, postconditioning, and remote conditioning. Pharmacol. Rev. *66*, 1142–1174.

16. Hausenloy, D.J., Garcia-Dorado, D., Bøtker, H.E., Davidson, S.M., Downey, J., Engel, F.B., Jennings, R., Lecour, S., Leor, J., Madonna, R., et al. (2017). Novel targets and future strategies for acute cardioprotection: Position Paper of the European Society of Cardiology Working Group on Cellular Biology of the Heart. Cardiovasc. Res. *113*, 564–585.

17. Sako, D., Grinberg, A.V., Liu, J., Davies, M.V., Castonguay, R., Maniatis, S., Andreucci, A.J., Pobre, E.G., Tomkinson, K.N., Monnell, T.E., et al. (2010). Characterization of the ligand binding functionality of the extracellular domain of activin receptor type IIb. J. Biol. Chem. *285*, 21037–21048.

18. Hulmi, J.J., Oliveira, B.M., Silvennoinen, M., Hoogaars, W.M., Ma, H., Pierre, P., Pasternack, A., Kainulainen, H., and Ritvos, O. (2013). Muscle protein synthesis, mTORC1/MAPK/Hippo signaling, and capillary density are altered by blocking of myostatin and activins. Am. J. Physiol. Endocrinol. Metab. *304*, E41–E50.

19. Nissinen, T.A., Degerman, J., Räsänen, M., Poikonen, A.R., Koskinen, S., Mervaala, E., Pasternack, A., Ritvos, O., Kivelä, R., and Hulmi, J.J. (2016). Systemic blockade of ACVR2B ligands prevents chemotherapy-induced muscle wasting by restoring muscle protein synthesis without affecting oxidative capacity or atrogenes. Sci. Rep. *6*, 32695.

20. Hulmi, J.J., Nissinen, T.A., Räsänen, M., Degerman, J., Lautaoja, J.H., Hemanthakumar, K.A., Backman, J.T., Ritvos, O., Silvennoinen, M., and Kivelä, R. (2018). Prevention of chemotherapy-induced cachexia by ACVR2B ligand blocking has different effects on heart and skeletal muscle. J. Cachexia Sarcopenia Muscle *9*, 417–432.

21. Juhaszova, M., Zorov, D.B., Yaniv, Y., Nuss, H.B., Wang, S., and Sollott, S.J. (2009). Role of glycogen synthase kinase-3beta in cardioprotection. Circ. Res. *104*, 1240–1252.

22. de Kretser, D.M., O'Hehir, R.E., Hardy, C.L., and Hedger, M.P. (2012). The roles of activin A and its binding protein, follistatin, in inflammation and tissue repair. Mol. Cell. Endocrinol. *359*, 101–106.

23. Boström, P., Mann, N., Wu, J., Quintero, P.A., Plovie, E.R., Panáková, D., Gupta, R.K., Xiao, C., MacRae, C.A., Rosenzweig, A., and Spiegelman, B.M. (2010). C/EBPβ controls exercise-induced cardiac growth and protects against pathological cardiac remodeling. Cell *143*, 1072–1083.

24. Bezzerides, V.J., Platt, C., Lerchenmüller, C., Paruchuri, K., Oh, N.L., Xiao, C., Cao, Y., Mann, N., Spiegelman, B.M., and Rosenzweig, A. (2016). CITED4 induces physiologic hypertrophy and promotes functional recovery after ischemic injury. JCI Insight *1*, e85904.

25. Carvalho, F.S., Burgeiro, A., Garcia, R., Moreno, A.J., Carvalho, R.A., and Oliveira, P.J. (2014). Doxorubicin-induced cardiotoxicity: from bioenergetic failure and cell death to cardiomyopathy. Med. Res. Rev. *34*, 106–135.

26. Kerkelä, R., Ulvila, J., and Magga, J. (2015). Natriuretic Peptides in the Regulation of Cardiovascular Physiology and Metabolic Events. J. Am. Heart Assoc. *4*, e002423.

27. Pavo, N., Lukovic, D., Zlabinger, K., Zimba, A., Lorant, D., Goliasch, G., Winkler, J., Pils, D., Auer, K., Jan Ankersmit, H., et al. (2017). Sequential activation of different pathway networks in ischemia-affected and non-affected myocardium, inducing intrinsic remote conditioning to prevent left ventricular remodeling. Sci. Rep. *7*, 43958.

28. Sartori, R., Schirwis, E., Blaauw, B., Bortolanza, S., Zhao, J., Enzo, E., Stantzou, A., Mouisel, E., Toniolo, L., Ferry, A., et al. (2013). BMP signaling controls muscle mass. Nat. Genet. *45*, 1309–1318.

29. Chen, J.L., Walton, K.L., Hagg, A., Colgan, T.D., Johnson, K., Qian, H., Gregorevic, P., and Harrison, C.A. (2017). Specific targeting of TGF-β family ligands demonstrates distinct roles in the regulation of muscle mass in health and disease. Proc. Natl. Acad. Sci. USA *114*, E5266–E5275.

30. Latres, E., Mastaitis, J., Fury, W., Miloscio, L., Trejos, J., Pangilinan, J., Okamoto, H., Cavino, K., Na, E., Papatheodorou, A., et al. (2017). Activin A more prominently regulates muscle mass in primates than does GDF8. Nat. Commun. *8*, 15153.

31. McPherron, A.C., Lawler, A.M., and Lee, S.J. (1997). Regulation of skeletal muscle mass in mice by a new TGF-beta superfamily member. Nature *387*, 83–90.

32. Matsakas, A., Otto, A., Elashry, M.I., Brown, S.C., and Patel, K. (2010). Altered primary and secondary myogenesis in the myostatin-null mouse. Rejuvenation Res. *13*, 717–727.

33. Steelman, C.A., Recknor, J.C., Nettleton, D., and Reecy, J.M. (2006). Transcriptional profiling of myostatin-knockout mice implicates Wnt signaling in postnatal skeletal muscle growth and hypertrophy. FASEB J. *20*, 580–582.

34. Welle, S., Cardillo, A., Zanche, M., and Tawil, R. (2009). Skeletal muscle gene expression after myostatin knockout in mature mice. Physiol. Genomics *38*, 342–350.

35. Cadena, S.M., Tomkinson, K.N., Monnell, T.E., Spaits, M.S., Kumar, R., Underwood, K.W., Pearsall, R.S., and Lachey, J.L. (2010). Administration of a soluble activin type IIB receptor promotes skeletal muscle growth independent of fiber type. J. Appl. Physiol. (1985) *109*, 635–642.

36. Dogra, D., Ahuja, S., Kim, H.T., Rasouli, S.J., Stainier, D.Y.R., and Reischauer, S. (2017). Opposite effects of Activin type 2 receptor ligands on cardiomyocyte proliferation during development and repair. Nat. Commun. *8*, 1902.

37. Javadov, S., Jang, S., and Agostini, B. (2014). Crosstalk between mitogen-activated protein kinases and mitochondria in cardiac diseases: therapeutic perspectives. Pharmacol. Ther. *144*, 202–225.

38. Lessard, S.J., MacDonald, T.L., Pathak, P., Han, M.S., Coffey, V.G., Edge, J., Rivas, D.A., Hirshman, M.F., Davis, R.J., and Goodyear, L.J. (2018). JNK regulates muscle remodeling via myostatin/SMAD inhibition. Nat. Commun. *9*, 3030.

39. Pachori, A.S., Custer, L., Hansen, D., Clapp, S., Kemppa, E., and Klingensmith, J. (2010). Bone morphogenetic protein 4 mediates myocardial ischemic injury through JNK-dependent signaling pathway. J. Mol. Cell. Cardiol. *48*, 1255–1265.

40. Ogura, Y., Ouchi, N., Ohashi, K., Shibata, R., Kataoka, Y., Kambara, T., Kito, T., Maruyama, S., Yuasa, D., Matsuo, K., et al. (2012). Therapeutic impact of follistatin-like 1 on myocardial ischemic injury in preclinical models. Circulation *126*, 1728–1738.

41. Oshima, Y., Ouchi, N., Sato, K., Izumiya, Y., Pimentel, D.R., and Walsh, K. (2008). Follistatin-like 1 is an Akt-regulated cardioprotective factor that is secreted by the heart. Circulation *117*, 3099–3108.

42. Seki, M., Powers, J.C., Maruyama, S., Zuriaga, M.A., Wu, C.L., Kurishima, C., Kim, L., Johnson, J., Poidomani, A., Wang, T., et al. (2018). Acute and Chronic Increases of Circulating FSTL1 Normalize Energy Substrate Metabolism in Pacing-Induced Heart Failure. Circ Heart Fail *11*, e004486.

43. McPherron, A.C., Lawler, A.M., and Lee, S.J. (1999). Regulation of anterior/posterior patterning of the axial skeleton by growth/differentiation factor 11. Nat. Genet. *22*, 260–264.

44. McPherron, A.C., Huynh, T.V., and Lee, S.J. (2009). Redundancy of myostatin and growth/differentiation factor 11 function. BMC Dev. Biol. *9*, 24.

45. Loffredo, F.S., Steinhauser, M.L., Jay, S.M., Gannon, J., Pancoast, J.R., Yalamanchi, P., Sinha, M., Dall'Osso, C., Khong, D., Shadrach, J.L., et al. (2013). Growth differentiation factor 11 is a circulating factor that reverses age-related cardiac hypertrophy. Cell *153*, 828–839.

46. Schafer, M.J., Atkinson, E.J., Vanderboom, P.M., Kotajarvi, B., White, T.A., Moore, M.M., Bruce, C.J., Greason, K.L., Suri, R.M., Khosla, S., et al. (2016). Quantification of GDF11 and Myostatin in Human Aging and Cardiovascular Disease. Cell Metab. *23*, 1207–1215.

47. Harper, S.C., Brack, A., MacDonnell, S., Franti, M., Olwin, B.B., Bailey, B.A., Rudnicki, M.A., and Houser, S.R. (2016). Is Growth Differentiation Factor 11 a Realistic Therapeutic for Aging-Dependent Muscle Defects? Circ. Res. *118*, 1143–1150, discussion 1150.

48. Egerman, M.A., Cadena, S.M., Gilbert, J.A., Meyer, A., Nelson, H.N., Swalley, S.E., Mallozzi, C., Jacobi, C., Jennings, L.L., Clay, I., et al. (2015). GDF11 Increases with Age and Inhibits Skeletal Muscle Regeneration. Cell Metab. *22*, 164–174.

49. Hammers, D.W., Merscham-Banda, M., Hsiao, J.Y., Engst, S., Hartman, J.J., and Sweeney, H.L. (2017). Supraphysiological levels of GDF11 induce striated muscle atrophy. EMBO Mol. Med. *9*, 531–544.

50. Du, G.Q., Shao, Z.B., Wu, J., Yin, W.J., Li, S.H., Wu, J., Weisel, R.D., Tian, J.W., and Li, R.K. (2017). Targeted myocardial delivery of GDF11 gene rejuvenates the aged mouse heart and enhances myocardial regeneration after ischemia-reperfusion injury. Basic Res. Cardiol. *112*, 7.

51. Lee, J.Y., Hopkinson, N.S., and Kemp, P.R. (2011). Myostatin induces autophagy in skeletal muscle in vitro. Biochem. Biophys. Res. Commun. *415*, 632–636.

52. Seldin, M.M., Kim, E.D., Romay, M.C., Li, S., Rau, C.D., Wang, J.J., Krishnan, K.C., Wang, Y., Deb, A., and Lusis, A.J. (2017). A systems genetics approach identifies *Trp53inp2* as a link between cardiomyocyte glucose utilization and hypertrophic response. Am. J. Physiol. Heart Circ. Physiol. *312*, H728–H741.

53. Fillmore, N., Mori, J., and Lopaschuk, G.D. (2014). Mitochondrial fatty acid oxidation alterations in heart failure, ischaemic heart disease and diabetic cardiomyopathy. Br. J. Pharmacol. *171*, 2080–2090.

54. Biesemann, N., Mendler, L., Wietelmann, A., Hermann, S., Schäfers, M., Krüger, M., Boettger, T., Borchardt, T., and Braun, T. (2014). Myostatin regulates energy homeostasis in the heart and prevents heart failure. Circ. Res. *115*, 296–310.

55. Jaswal, J.S., Keung, W., Wang, W., Ussher, J.R., and Lopaschuk, G.D. (2011). Targeting fatty acid and carbohydrate oxidation–a novel therapeutic intervention in the ischemic and failing heart. Biochim. Biophys. Acta *1813*, 1333–1350.

56. Lopaschuk, G.D., Ussher, J.R., Folmes, C.D., Jaswal, J.S., and Stanley, W.C. (2010). Myocardial fatty acid metabolism in health and disease. Physiol. Rev. *90*, 207–258.

57. Ruas, J.L., White, J.P., Rao, R.R., Kleiner, S., Brannan, K.T., Harrison, B.C., Greene, N.P., Wu, J., Estall, J.L., Irving, B.A., et al. (2012). A PGC-1α isoform induced by resistance training regulates skeletal muscle hypertrophy. Cell *151*, 1319–1331.

58. Koncarevic, A., Kajimura, S., Cornwall-Brady, M., Andreucci, A., Pullen, A., Sako, D., Kumar, R., Grinberg, A.V., Liharska, K., Ucran, J.A., et al. (2012). A novel therapeutic approach to treating obesity through modulation of TGFβ signaling. Endocrinology *153*, 3133–3146.

59. Burwell, L.S., Nadtochiy, S.M., and Brookes, P.S. (2009). Cardioprotection by metabolic shut-down and gradual wake-up. J. Mol. Cell. Cardiol. *46*, 804–810.

60. Basheer, W.A., Fu, Y., Shimura, D., Xiao, S., Agvanian, S., Hernandez, D.M., Hitzeman, T.C., Hong, T., and Shaw, R.M. (2018). Stress response protein GJA1-20k promotes mitochondrial biogenesis, metabolic quiescence, and cardioprotection against ischemia/reperfusion injury. JCI Insight *3*, 121900.

61. Latres, E., Pangilinan, J., Miloscio, L., Bauerlein, R., Na, E., Potocky, T.B., Huang, Y., Eckersdorff, M., Rafique, A., Mastaitis, J., et al. (2015). Myostatin blockade with a fully human monoclonal antibody induces muscle hypertrophy and reverses muscle atrophy in young and aged mice. Skelet. Muscle *5*, 34.

62. Lach-Trifilieff, E., Minetti, G.C., Sheppard, K., Ibebunjo, C., Feige, J.N., Hartmann, S., Brachat, S., Rivet, H., Koelbing, C., Morvan, F., et al. (2014). An antibody blocking activin type II receptors induces strong skeletal muscle hypertrophy and protects from atrophy. Mol. Cell. Biol. *34*, 606–618.

63. Lee, S.J., Reed, L.A., Davies, M.V., Girgenrath, S., Goad, M.E., Tomkinson, K.N., Wright, J.F., Barker, C., Ehrmantraut, G., Holmstrom, J., et al. (2005). Regulation of muscle growth by multiple ligands signaling through activin type II receptors. Proc. Natl. Acad. Sci. USA *102*, 18117–18122.

64. Zhou, X., Wang, J.L., Lu, J., Song, Y., Kwak, K.S., Jiao, Q., Rosenfeld, R., Chen, Q., Boone, T., Simonet, W.S., et al. (2010). Reversal of cancer cachexia and muscle wasting by ActRIIB antagonism leads to prolonged survival. Cell *142*, 531–543.

65. Pirruccello-Straub, M., Jackson, J., Wawersik, S., Webster, M.T., Salta, L., Long, K., McConaughy, W., Capili, A., Boston, C., Carven, G.J., et al. (2018). Blocking extracellular activation of myostatin as a strategy for treating muscle wasting. Sci. Rep. *8*, 2292.

66. Gao, E., Lei, Y.H., Shang, X., Huang, Z.M., Zuo, L., Boucher, M., Fan, Q., Chuprun, J.K., Ma, X.L., and Koch, W.J. (2010). A novel and efficient model of coronary artery ligation and myocardial infarction in the mouse. Circ. Res. *107*, 1445–1453.

YMTHE, Volume *27*

Supplemental Information

Systemic Blockade of ACVR2B Ligands Protects Myocardium from Acute Ischemia-Reperfusion Injury

Johanna Magga, Laura Vainio, Teemu Kilpiö, Juha J. Hulmi, Saija Taponen, Ruizhu Lin, Markus Räsänen, Zoltán Szabó, Erhe Gao, Lea Rahtu-Korpela, Tarja Alakoski, Johanna Ulvila, Mika Laitinen, Arja Pasternack, Walter J. Koch, Kari Alitalo, Riikka Kivelä, Olli Ritvos, and Risto Kerkelä

Supplemental Methods and Materials

Figure S1.

(**A**) Timeline for the IR study. (**B**) Echocardiography analysis at 24h after IR injury. Representative M mode echocardiography images are shown. Mice were treated with vehicle, soluble decoy receptor ACVR2B-Fc only at reperfusion or pretreatment of ACVR2B-Fc 24h prior to/at reperfusion. IR was achieved by transient ligation of the left anterior descending coronary artery for 30 min, followed by reperfusion for 24h.

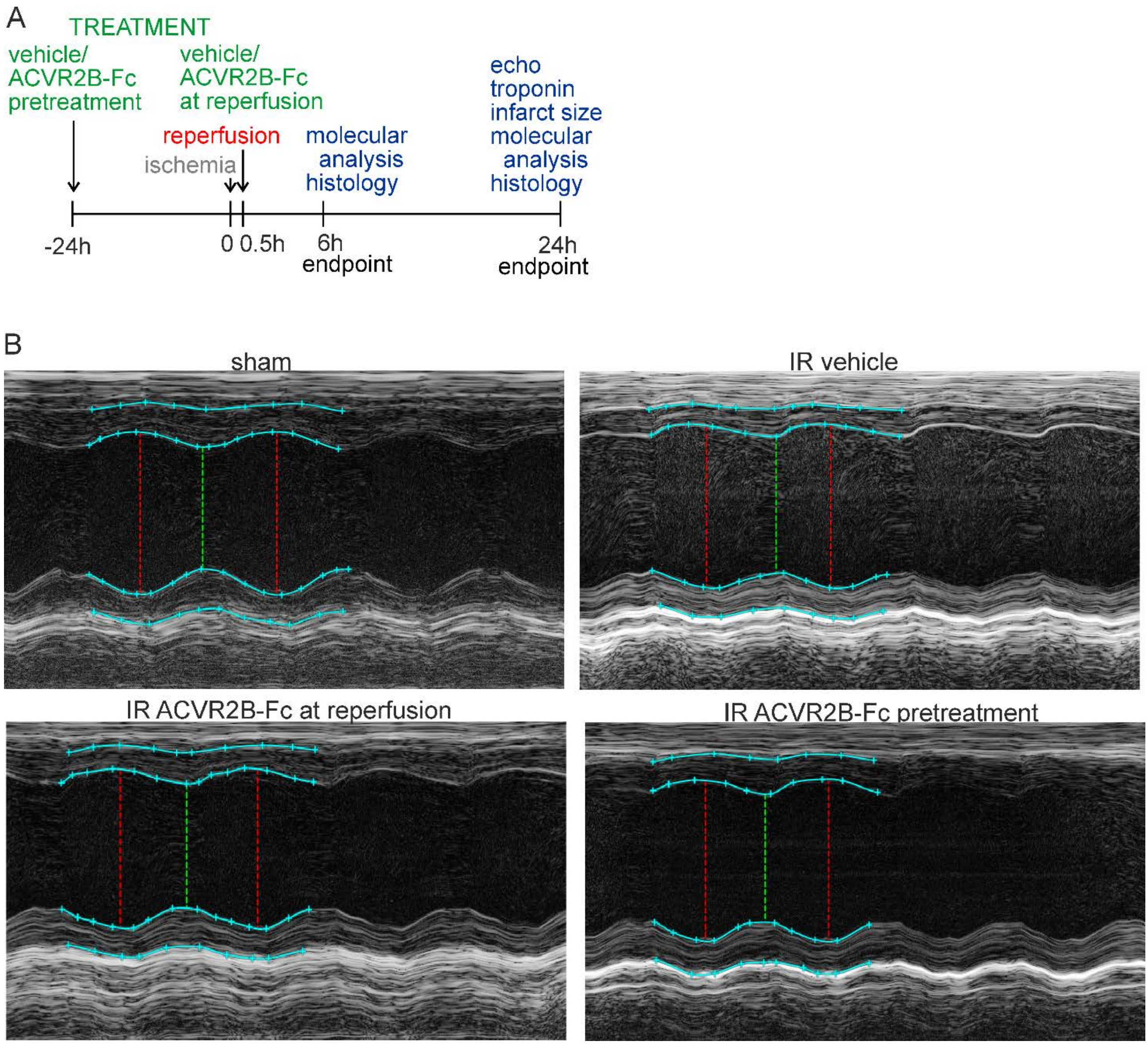

Figure S2.

(**A)** ACVR2B treatment (single dose of 10 mg/kg s.c.) for 48h increased cardiomyocyte cross-sectional area in left ventricles of the healthy mice. (**B**) ACVR2B increases skeletal muscle cross-sectional area in the quadriceps of the healthy mice. (**C**) ACVR2B-Fc induced hypertrophy was accompanied by increased, but transient phosphorylation of GSK3β at Ser9. (**D**) ACVR2B-Fc reduced apoptosis in the left ventricles 6h after IR injury as analyzed by TUNEL. (E) TUNEL positive nuclei were localized in cardiomyocytes stained with sarcomeric α-actinin. Scale bar 50 µm. n=5 and 6 (A, C), n=4 and 5 (B). Data are presented as mean ± SD. *P<0.05, **P<0.01.

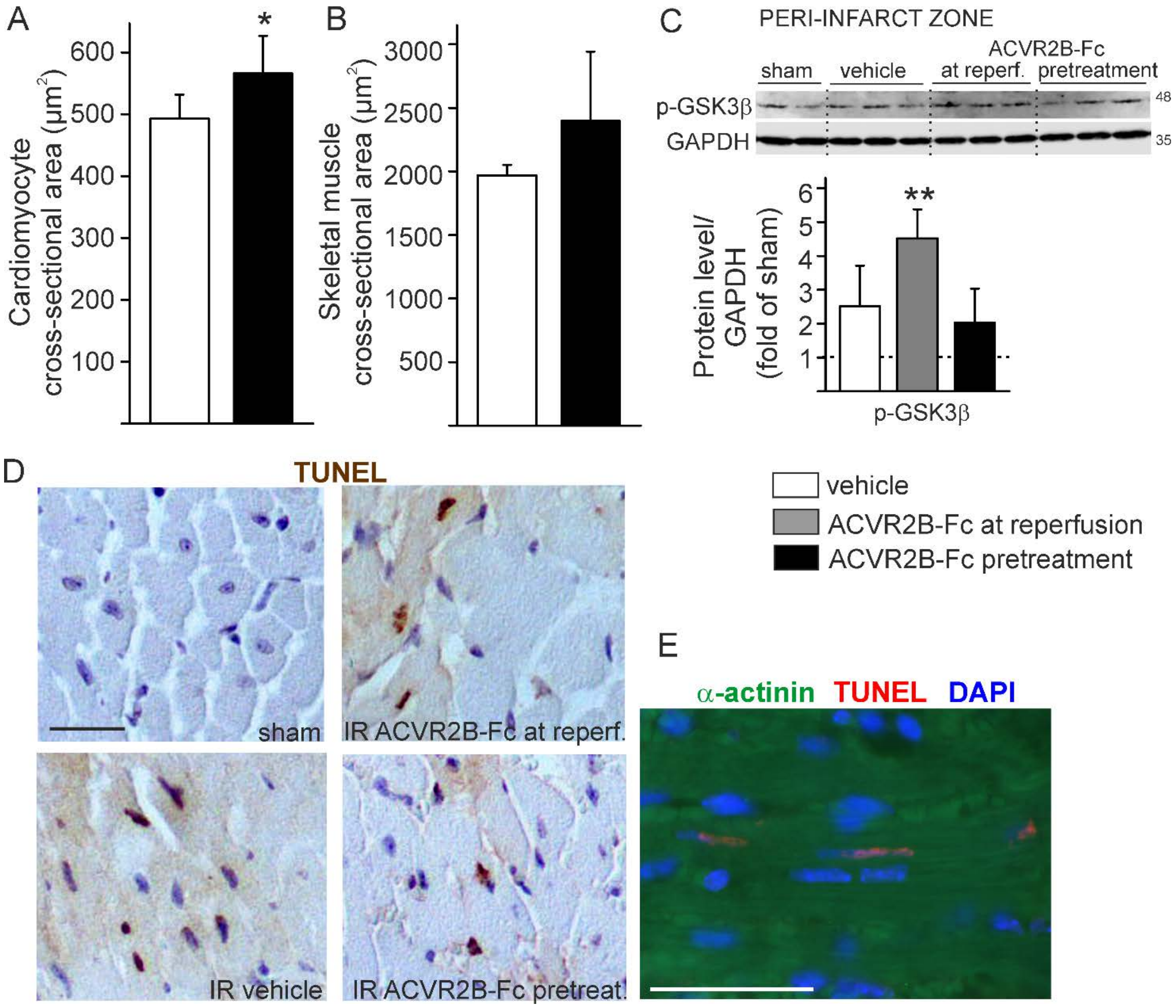

Figure S3.

(**A**) ACVR2B-Fc did not affect infiltration of inflammatory cells into LV at 24h after IR as analyzed with granulocyte stain. (**B**) ACVR2B-Fc did not affect inflammatory response at 6 or 24h after IR as analyzed with qPCR from left ventricle. Scale bar 50 µm. n=5, 6 (A, 6h IR), n=10, 6 (A, 24h IR), n=5, 6 (B, 6h IR), n=9, 8 (B, 24h IR). Data are presented as mean ± SD.

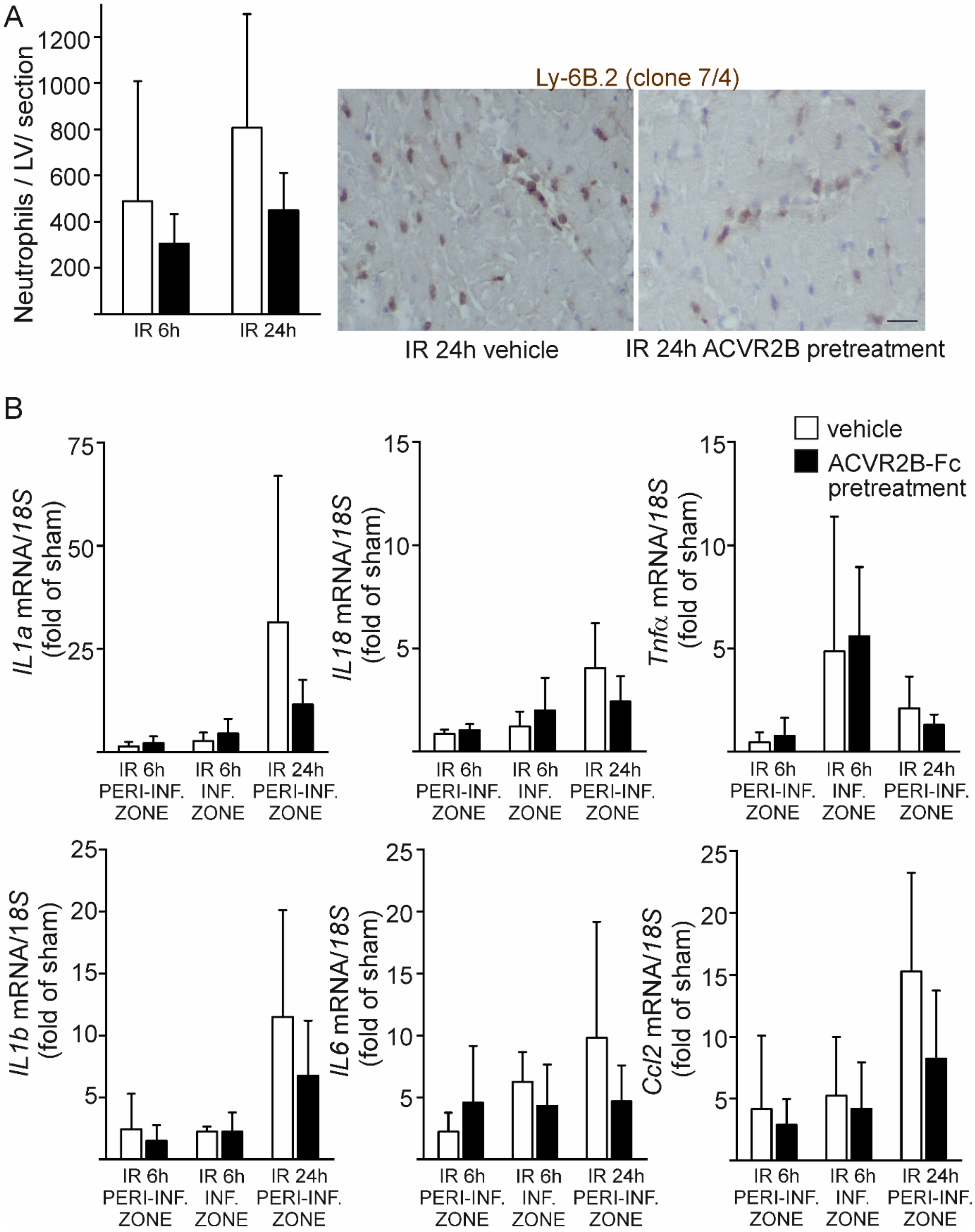

Figure S4.

(**A**) ACVR2B ligands did not induce BRE-luc SMAD activity unlike BMPR ligand BMP4 as determined in neonatal rat ventricular cardiomyocytes. (**B-C**) ACVR2B ligands activin A, activin B and myostatin levels were increased in LV infarct and peri-infarct area, but not remote area, 6h and 24h after IR. (**D**) ACVR2B-Fc increased phosphorylation of acetyl CoA-carboxylase in sham-operated mice but not in IR-operated mice analyzed 24h after IR and 48h after ACVR2B-Fc treatment. Scale bar 50 µm. n=6 (A). Data are presented as mean ± SD. *P<0.05, ***P<0.001.

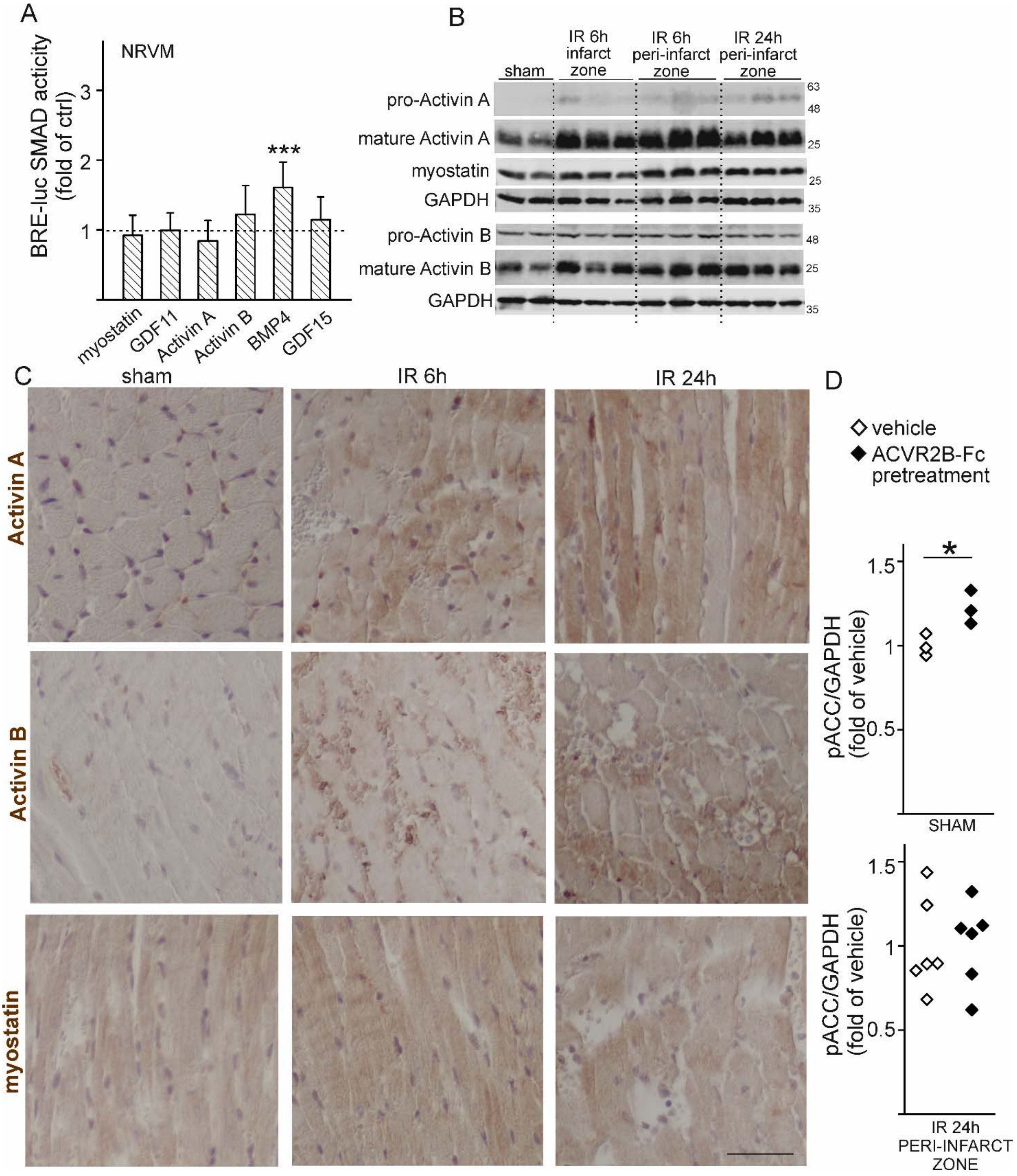

Table S1.

Oligonucleotide primer and detection probe sequences used for mRNA quantitation by qPCR.

Gene	Sequences<br>Fluorogenic Probe (5’-[FAM…TAMRA])	Sense (5’-…)	Antisense (5’-…)
**Activin A (Inhba)**	-	GAACGGGTATGTGGAGATAG	TGAAATAGACGGATGGTGAC
**Activin B (Inhbb)**	-	CTTCGTCTCTAATGAAGGCAACC	CTCCACCACATTCCACCTGTC
**Acvr2A**	-	GTTACACCGAAGCCACCCTA	AACCAAATCTTCCCCTTGCT
**Acvr2B**	-	TTAAGGATCACTGGCTGAAACA	GGATACCCGCTCTTCTACACAG
**ANP (Nppa)**	TCGCTGGCCCTCGGAGCCT	GAAAAGCAAACTGAGGGCTCTG	CCTACCCCCGAAGCAGCT
**Bmpr2**	-	TTGGACTCATCTACTGGGAGGT	TGGACACAAGAACCTGCATATC
**BNP (Nppb)**	CATCATTGCCTGGCCCATCGC	AGGCGAGACAAGGGAGAACA	GGAGATCCATGCCGCAGA
**Ccl2**	CACTCACCTGCTGCTACTCATTCACTGGC	CTCAGCCAGATGCAGTTAATGC	AGCCGACTCATTGGGATCAT
**Cited4**	-	CCTGGCATACGGCTCCTTC	AGACTGCAGGTGCGTGCTAC
**Col1a1**	CTTTGCTTCCCAGATGTCCTATGGCTATGATG	CCCTGGCCTTGGAGGAA	CACGGAAACTCCAGCTGATTTT
**Col3a1**	TGGTGAACGTGGCTCTAATGGCATCA	CCACGAGGTGACAAAGGTGA	GCCAGGGAATCCTCGATGT
**Cytohrome C (Cycs)**	.	GGAGGCAAGCATAAGACTGG	TCCATCAGGGTATCCTCTCC
**Gapdh**	CTGCCGATGCCCCCATGTTTG	GGTCATCATCTCCGCCCC	TTCTCGTGGTTCACACCCATC
**Gdf11**	-	CTTGGAAGAGGACGAGTACCAC	CTGAAGTGGAAATGACAGCAGA
**Glut1**	-	CTCTGTCGGCCTCTTTGTTAAT	CCAGTTTGGAGAAGCCCATAAG
**Glut4**	-	GTGACTGGAACACTGGTCCTA	CCAGCCACGTTGCATTGTAG
**IL1a**	-	CGAAGACTACAGTTCTGCCATT	GACGTTTCAGAGGTTCTCAGAG
**IL1b**	-	TCTTTGAAGTTGACGGACCC	TGAGTGATACTGCCTGCCTG
**IL6**	CAGAATTGCCATTGCACAACTCTTTTCTCA	ACATGTTCTCTGGGAAATCGTGGAA	TGCATCATCGTTGTTCATACAA
**IL18**	-	GACTCTTGCGTCAACTTCAAGG	CAGGCTGTCTTTTGTCAACGA
**MHCα (Myh6)**	CGAGGAATAACCTCTCCAGCAGACCCTC	GGTGCCAAGAAGATGCACG	TTATGTTTATTGTGTATTGGCCACAG
**MHCβ (Myh7)**	AGCCCTCAGACCTGGAGCCTTTGC	AGCTCTAAGGGTGCCCGTG	TGCTTCCACCTAAAGGGCTG
**Mstn**	-	AAGATGGGCTGAATCCCTTT	GCAGTCAAGCCCAAAGTCTC
**Pfkm**	CTGACACAGCACTGAACACC	CGTCCCTGGGTCAGACTTC	CAGACTGCTTGATTCGGTCAC

	ATCTGCAC	AG	A
**Pgam1**	CCCTTCTACAGCAACATCAGCAAGGATC	TTATGATGTCCCACCGCCT	GGTCTTCAGTAAGGTCTGCGTACCT
**Pgc1a1**	-	GGACATGTGCAGCCAAGACTCT	CACTTCAATCCACCCAGAAAGCT
**Pgc1a4**	-	TCACACCAAACCCACAGAAA	CTGGAAGATATGGCACAT
**Tgfβ1 (Tgfb1)**	ACGGAAGCGCATCGAAGCCATC	CATCGACATGGAGCTGGTGA	TTGGACAGGATCTGGCCAC
**Tnfα (Tnf)**	TGCTCCTCACCCACACCGTCAGC	GACAAGGCTGCCCCGACTA	CTCCTGGTATGAGATAGCAAATC
**18S**	CCTGGTGGTGCCCTTCCGTCA	TGGTTGCAAAGCTGAAACTTAAAG	AGTCAAATTAAGCCGCAGGC
**18S**	-	CGCCGCTAGAGGTGAAATTC	CCAGTCGGCATCGTTTATGG

Table S2. ACVR2B-Fc preserves cardiac function in ischemia-reperfusion injury.

Mice were subjected to a sham operation or ischemia-reperfusion injury (IR) and treated with either vehicle or ACVR2B-Fc only at reperfusion or pretreatment with ACVR2B-Fc 24h prior to/at reperfusion. After 24h mice were subjected to echocardiography analysis. Ejection fraction (EF), fractional shortening (FS), heart rate (HR), left ventricular (LV) mass, LV internal diameter in diastole (LVID;d) and systole (LVID;s), LV posterior wall thicknesses (LVPW;d, LVPW;s), endocardial areas (ENDO;d, ENDO;s) and endocardial fractional area change (ENDO-FAC). Data are presented as mean±SD; *P<0.05, ** P<0.01, *** P<0.001 versus sham, # p<0.05, ## p<0.01, ### p<0.001 versus IR vehicle.

	**sham** n=13	**IR vehicle** n=19	**IR ACVR2B-Fc at reperfusion** n=9	**IR ACVR2B-Fc pretreatment** n=18
**EF (%)**	50.4±8.9	36.4±11.9**	39.1±7.4	48.7±11.2##
**FS (%)**	25.6±5.4	17.6±6.5**	18.8±4.1	24.5±6.7##
**HR (BPM)**	415±25	460±48	477±35*	445±66
**LV mass (mg)**	91±18	96±20	96±12	105±21
**LVID;d (mm)**	4.19±0.25	4.36±0.36	4.04±0.25#	4.00±0.33##
**LVID;s (mm)**	3.12±0.33	3.61±0.51*	3.29±0.33	3.03±0.44###
**LVPW;d (mm)**	0.70±0.07	0.72±0.17	0.75±0.03	0.84±0.11*#
**LVPW;s (mm)**	0.99±0.16	0.89±0.29	0.94±0.08	1.11±0.18*#
**ENDO;d (mm^2)**	12.6±1.2	13.5±1.9	12.8±1.1	12.0±1.8#
**ENDO;s (mm^2)**	7.1±1.3	9.5±2.3**	8.8±1.3	7.3±2.2##
**ENDO-FAC (%)**	43.4±6.9	30.1±9.0***	31.7±6.8*	40.3±11.9##

Table S3. Blood cell count of ACVR2B-Fc and vehicle-treated mice.

Mice were treated with vehicle or ACVR2B-Fc 24h prior to/at reperfusion and subjected to ischemia-reperfusion injury (IR). After 24h blood was collected and analyzed with veterinary hematology analyzer for red blood cell analysis and white blood cell differential count. Red blood cell count (RBC), mean cell volume (MCV), hematocrit (HCT), mean cell hemoglobin (MCH), MCH concentration (MCHC), RBC distribution width (RDWR), RDWR absolute volume (RDWA), hemoglobin (HGB), white blood cell count (WBC), absolute counts for lymphocytes (LA), monocytes (MA) and granulocytes (GA). Data are presented as mean±SD.

	**IR vehicle n=8**	**IR ACVR2B-Fc pretreatment n=6**
**RBC (10^9/ml)**	8.3±0.4	8.3±1.2
**MCV (fL)**	39.4±0.2	39.2±0.4
**HCT (%)**	32.6±1.6	32.4±5.0
**MCH (pg)**	15.7±0.2	15.7±0.2
**MCHC (g/dl)**	39.8±0.4	40.0±0.7
**RDWR (%)**	19.9±0.3	19.7±0.2
**RDWA (fL)**	26.4±0.3	26.0±0.6
**HGB (g/dl)**	13.0±0.6	13.0±1.9
**WBC (10^6/ml)**	6.1±1.7	5.0±1.9
**LA (10^6/ml)**	4.6±1.4	3.2±1.0
**MA (10^6/ml)**	0.35±0.12	0.32±0.12
**GA (10^6/ml)**	1.14±0.44	1.53±0.87

Table S4. ACVR2B-Fc preserves cardiac function in doxorubicin-induced cardiomyopathy.

Mice were treated with doxorubicin for two weeks to induce cumulative cardiotoxicity, followed by two additional weeks to develop drug-induced cardiomyopathy. Mice were treated with ACVR2B-Fc or vehicle throughout 4-week study, and subjected to echocardiography analysis. Ejection fraction (EF), fractional shortening (FS), left ventricular (LV) mass, LV internal diameter in diastole (LVID;d) and systole (LVID;s) and LV posterior wall thicknesses (LVPW;d, LVPW;s). Data are presented as mean±SD; *P<0.05, versus control, # P<0.05 versus doxorubicin with vehicle.

	**control**<br><br>**n=9**	**Doxorubicin vehicle**<br>**n=6**	**Doxorubicin ACVR2B-Fc**<br>**n=9**
**EF (%)**	57.4±6.3	47.8±6.8*	54.4±2.2#
**FS (%)**	30.1±4.2	23.9±4.0*	27.1±2.5
**LV mass (mg)**	109±19	91±25	91±12
**LVID;d (mm)**	4.36±0.35	4.25±0.23	4.16±0.15
**LVID;s (mm)**	3.05±0.38	3.23±0.23	3.03±0.16
**LVPW;d (mm)**	0.78±0.07	0.74±0.14	0.75±0.10
**LVPW;s (mm)**	1.13±0.09	0.97±0.15	1.05±0.11

Supplemental Methods

Ischemia-reperfusion

Experimental protocols were approved by the Animal Use and Care Committee of the University of Oulu and the Regional State Administrative Agency of Southern Finland (ESAVI-2010-03931/ Ym-23). The mice were maintained in plastic cages at a constant 21°C temperature with a 12 h light–dark cycle and had free access to food (Teklad Global Rodent diet, Harlan) and water. Immediately after operation, mice were kept in warm room at 25°C temperature overnight.

8-10 week old male C57BL/6J mice (Harlan) were subjected to ischemia-reperfusion (IR) by ligation of the left anterior descending coronary artery (LAD) for 30 minutes, after which the slip knot was released allowing reperfusion of the ischemic myocardium for 6 or 24h as previously described.[1] Male mice were used for the study to have more homogenous population for quantitation of the IR injury. Sham-operated mice were subjected to the same surgical procedure without ligation of the LAD. All operations were performed under isoflurane anaesthesia (Baxter, Vetequip evaporiser, 2% isoflurane with 1 l/min oxygen flow). Carprofen (Rimadyl) 5 mg/kg s.c. (injection volume 0.125-0.15 ml) and buprenorphine (Vetergesic) 0.05 mg/kg s.c. (injection volume 0.1-0.3 ml) were administred as peri-operation analgesia. Post-operation dehydration was prevented with s.c. administration of 5% glucose solution (injection volume 0.5-1.0 ml). All animals were monitored after the surgery and received a dose of buprenorphine (0.05 mg/kg) in the evening of the operation day. Another dose of buprenorphine and a dose of carprofen (5mg/kg) were administered the following morning.

ACVR2B-Fc treatment

ACVR2B-Fc recombinant fusion protein was produced as described earlier.[2] The ectodomain of human ACVR2B was fused with a human IgG1 Fc domain and expressed in Chinese hamster ovary cells grown in suspension culture. The protein is similar but not identical to that originally generated by Lee et al.[3]. ACVR2B-Fc was administered as 10 mg/kg s.c. 24h prior to IR and at reperfusion (termed “ACVR2B-Fc pretreatment”) or only at reperfusion (termed “ACVR2B-Fc at reperfusion”). The same volume of PBS or human IgG1-Fc control was used as a vehicle control. Experimental timeline is depicted in Figure S1.

The littermates were randomly and equally divided into treatment groups to avoid any bias between the mouse litters. *6h endpoint:* In 6h endpoint group, one mouse from IR vehicle group died at operation. Group sizes for the analysis were following: sham n=6, IR vehicle n=5, IR ACVR2B-Fc pretreatment n=6, ACVR2B-Fc at reperfusion n=6. *24h endpoint:* In the 24h endpoint group, one mouse from IR vehicle group and 4 mice from ACVR2B-Fc pretreatment group died during the operation. Group sizes for the analysis were following: sham n=13, IR vehicle n=19, IR ACVR2B-Fc pretreatment n=18, ACVR2B-Fc at reperfusion n=9. All mice in 24h endpoint were subjected to echocardiography. A subgroup of sham n=5, IR vehicle n=9, IR ACVR2B-Fc pretreatment n=10 mice were used for cardiac troponin I assay and infarct size determination by TTC stain. The rest of the mice were used for qPCR, western blotting and histological analysis. The person operating on the mice was blinded from treatment. All analysis to assess functional, histological and other outcome were performed in a blinded manner.

Echocardiography

Mice were anaesthetized with isoflurane and transthoracic echocardiography was performed with Vevo 2100 high frequency, high resolution linear array ultrasound system using a MS-550S transducer (Visual Sonics Vevo 2100, 40 MHz, axial resolution 40 µm, lateral resolution 90 µm). B-mode, M-mode, transmitral flow - Pulse wave and tissue Doppler images were recorded and analyzed with Vevo Workstation software 1.7 by a blinded observer.

Determination of infarct size

Determination of area at risk (AR) and infarct size was performed 24h after IR as described.[1] Mice were reanaesthetized and the ligature around the LAD was retied at the previous ligation site. Following this, 2% Evans blue dye in PBS was injected into the aorta and allowed to circulate uniformly into areas of the heart perfused by the open coronary arteries. The heart was then quickly excised, snap frozen on dry ice and cut into five sections from the apex to the base. Sections were then incubated in 1% triphenyltetrazolium chloride (TTC) (Sigma) solution in PBS 20 min at 37°C, fixed with phosphate-buffered 10% formalin (pH 7.0) for 20 min, rinsed in PBS and photographed. The area not at risk (ANAR; Evans blue-stained area), AR (TTC staining-positive, non-infarct, red) and infarct (TTC staining-negative, white) areas were quantified with the Nikon NIS-Elements BR 2.30 program. Blood samples were collected 24h after IR and cardiac troponin I was measured from plasma with high-sensitivity mouse cardiac troponin I ELISA kit (Life Diagnostics) according to manufacturer's instructions.

Cardiomyocyte cell cultures

Hypoxia and cell survival assays in adult ventricular cardiomyocytes

Adult mouse ventricular cardiomyocytes (AMVMs) and rat ventricular cardiomyocytes (ARVMs) were isolated from 8-12 week old male C57BL/6 mice or SD rats by retrograde perfusion and enzymatic digestion as described before.[4] Mice were deeply anesthetized with isoflurane. Rats were euthanized with CO_2, decapitated and after the excision of the heart aorta was cannulated. For retrograde perfusion, a perfusion buffer (Tyrode's buffer; 24.1 mM HEPES, 127.5 mM NaCl, 5.1 mM KCl, 0.4 mM NaH_2PO_4, 0.55 mM $MgCl_2$) supplemented with 10 mM 2,3-butandione monoxime (BDM, Sigma) and 5.5 mM glucose (Sigma) was used at a flow rate of 3 ml/min/mouse heart or 5 ml/min/rat heart for 1 min before switching to perfusion buffer with 1 mg/ml collagenase type 2 (Worthington) and 12.6 µM $CaCl_2$ (Sigma) and perfused at a flow rate of 3ml/min/mouse heart for 8 min or 10 ml/min/rat heart for 18 min. Then hearts were cut into small pieces and collected to a tube containing perfusion buffer with 5 % FBS, where gently homogenized with trituration. The suspension was filtered through 200 µm mesh before cardiomyocytes were pelleted (300 rpm (18G), 2 min) and resuspended into perfusion buffer including 5 % FBS. Ca^{2+} concentration was gradually increased to 1.26 mM. After Ca^{2+} reintroduction cardiomyocytes were pelleted and resuspended in plating medium (AMVM: αMEM with Hank's salt (21575-022, Thermo Fisher Scientific) supplemented with 5 % FBS, insulin-transferrin-selenium supplement (Thermo Fisher Scientific), 10 mM BDM, 2 mM L-glutamine (Sigma) and penicillin-streptomycin (Sigma); ARVM: αMEM with Earle's salt (10370-047, Thermo Fisher Scientific) supplemented with 5 % FBS, 10 mM HEPES, insulin-transferrin-selenium supplement, 10 mM BDM, 2 mM L-glutamine and penicillin-streptomycin. Cardiomyocytes were plated onto laminin-coated wells at

12 500 cells/cm^2 and incubated in humidified atmosphere at 37°C containing 2% or 5% CO_2 for AMVM and ARVM, respectively. After 2h incubation, nonattached cells were gently removed.

For ARVM hypoxia experiment on the following day, medium was changed to αMEM with Earle's salt supplemented with insulin-transferrin-selenium, 10 mM BDM and 2 mM L-glutamine. Oxygen levels were controlled at 0.1% O_2 in 5% CO_2 for 4h, then medium was changed back to normal cultivation medium in normoxic conditions for reperfusion. Control cells were treated with same media in normoxic conditions. To assess the efficacy of ACVR2B-Fc in hypoxia, 200 ng/ml ACVR2B-Fc or control IgG1-Fc was added 30 min before hypoxia and to reperfusion medium. Medium samples were collected 4h after reperfusion for cell toxicity assay while cell viability was assessed 20h later.

To determine the effect of ligands on cell viability in hypoxic conditions, 100 ng/ml myostatin, GDF11, activin A, activin B, TGFβ or GDF15 were applied for cells 30 min before hypoxia. Thereafter, ARVM were exposed to 4h hypoxia followed by 4h reperfusion as described above. Medium samples were collected 4h after reperfusion for cell toxicity assay while cell viability was assessed 20h later.

Cell toxicity was determined with Toxilight (Lonza) to measure cell membrane integrity by quantifying adenylate kinase released into the medium. Cell viability was measured with resazurin assay (Sigma) which quantifies the reduction of resazurin to a fluorescent compound resorufin within viable cells. For analysis, 10 µM resazurin (Sigma) was added to the cell culture medium and 1h later, medium samples were collected into 96-well plate and measured by excitation at 544 nm and emission at 595 nm on a multiplate reader (Victor Wallac).

Luciferase reporter assays and hypoxia in neonatal rat ventricular cardiomyocytes

Neonatal rat ventricular cardiomyocytes (NRVMs) were isolated from 2-4 day-old Sprague-Dawley rats as described earlier.[5] Rats were sacrificed by quick decapitation, the hearts were excised, the ventricles were minced and digested in 2 mg/ml collagenase type 2 (Worthington) in PBS supplemented with 50 µM $CaCl_2$. Freshly isolated cells were preplated in DMEM/F12 supplemented with 10% fetal bovine serum (FBS) and with penicillin-streptomycin for 2h to remove fibroblasts. Nonattached cells were collected, plated at 150 000 cells/cm^2 and incubated overnight before changing to serum-free culture media (CSFM; DMEM/F12, insulin-transferrin-selenin, 1 mM sodium pyruvate, 0.25% bovine serum albumin (BSA), 1 nM T_3, penicillin-streptomycin). 1-2 days after isolation, NRVMs were transfected with CAGA-luc[6] or BRE-luc luciferase constructs[7] (1.78 µg/ml) or control plasmid pEF-IRESp, combined with pRL-TK Renilla luciferase control reporter (0.89 µg/ml) with Lipofectamine (1:100, Invitrogen) in OptiMEM and incubated for 6h. Two days after transfection, cells were treated with 100 ng/ml myostatin, GDF11, activin A, activin B, TGFβ or GDF15 (Peprotech) for 2h. Thereafter, cells were rinsed with PBS and frozen at -70°C. Luciferase was measured with Dual Luciferase Reporter Assay System (#E1910, Promega) with Luminoskan microplate luminometer (Thermo Fisher Scientific). To determine SMAD-promoter activity, Firefly luciferase activity was divided by Renilla luciferase activity.

For NRVM hypoxia experiment, medium was changed to DMEM (31885-023, Thermo Fisher Scientific), supplemented with 2% FBS, penicillin-streptomycin after luc-transfection. Two days after transfection, NRVM were incubated in DMEM (11966-025, Thermo Fisher Scientific) supplemented with 10 mM deoxyglucose and 1 mM sodium dithionite (Fluka), placed in C-Chamber with oxygen levels controlled at 0.1% in 5% CO_2 with ProOx C21 O_2/CO_2 controller (BioSpherix). Control (normoxia) cells were incubated in DMEM supplemented with 10 mM

glucose. 200 ng/ml ACVR2B-Fc or control IgG1-Fc was added 30 min before hypoxia. SMAD-promoter activity was measured with luciferase reporter assay 4 h after hypoxia as described above. Gene expression changes in NRVM were analyzed with qPCR after 4h hypoxia followed by 5h reperfusion.

Bioenergetic assay for adult mouse ventricular cardiomyocytes

Vehicle or ACVR2B-Fc (10 mg/kg s.c.) was administered to 8-10 week old male C57BL/6 mice 48h prior to AMVM isolation. Assessment of cell energy metabolism was performed by Seahorse XFp (Agilent Technologies). Primary AMVM were seeded onto 8-well microplates at a density of 1500 cells/well in plating medium. After a 2-hour pre-plating, the cells were washed with plating medium and then incubated in αMEM medium supplemented with insulin-transferrin-selenium supplement (Thermo Fisher Scientific), 10 mM BDM and 2 mM L-glutamine. The cells were then subjected to either 4 hours of hypoxia controlled at 0.1% O_2 in 2% CO_2, or normoxia in 2% CO_2. After hypoxia, the medium was immediately changed to plating medium and incubated for 1h in 2% CO_2. Seahorse assay medium (Agilent) was supplemented with 10 mM BDM, 2 mM L-glutamine, 10 mM glucose and 1 mM sodium pyruvate, pH adjusted to 7.4 with 0.1 mM NaOH. The experiments were then carried out as per Agilent Seahorse XFp cell mito stress test protocol using 1 µM oligomycin, 1.5 µM FCCP and 0.5 µM rotenone + antimycin A. Homogeneity of cell seeding density among wells was verified by light microscopy prior to the assay. The test protocol was modified to perform 2 min measurements instead of the standard 3 min in order to avoid consuming the media oxygen reserve and possible restriction of cell oxidative metabolism. The results were analyzed using Agilent Wave and Graphpad Prism 7.

RNA isolation and quantitative real-time PCR

Mice were euthanized with CO_2 and sacrificed by decapitation. Heart was excised, frozen in liquid nitrogen, and stored at -70°C. RNA was isolated with Trizol (Invitrogen). cDNA was synthesized from 500 ng of RNA with Transcriptor First Strand cDNA synthesis kit (Roche). The expression levels were evaluated on an ABI Prism 7700 Sequence Detection System (Applied Biosystems) as previously described.[8] Oligonucleotide primer and detection probe sequences used for mRNA quantitation are presented in Table S1.

Western blotting

Total protein content was extracted from LV peri-infarct zone as described earlier.[9] The protein concentrations were determined with colorimetric protein assay (Bio-Rad Laboratories). Protein samples (40 µg) were denatured at 97°C for 5 min and loaded on sodium dodecyl sulfate polyacrylamide gel electrophoresis (SDS-PAGE), run at 200 V and transferred to 0.45 µm Optitran BA-S 85 nitrocellulose membrane (Whatman, GE Healthcare) at 100 V for 2 h. The membranes were incubated for 1 h at RT in blocking buffer (Odyssey, LI-COR) diluted into Tris-buffered saline (TBS, 50 mM Tris, 200 mM NaCl, pH 7.4). Membranes were then incubated overnight with primary antibody pSMAD2 (Ser465/467) (Cell Signaling #3108, used as 1:1000 dilution), pJNK (Promega #V7931, 1:5000), p-p38 (Thr180/Tyr182) (Cell Signaling #9211, 1:1000), pERK44/42 (Thr202/Tyr204) (Cell Signaling #9106, 1:2000), pAkt (Ser473) (Cell Signaling #9271, 1:1000), pACC (Cell Signaling #3661, 1:1000), Bim (Cell Signaling #2933, 1:1000), LC3 (Sigma, #L7543, 1:1000), pGSK3β (Ser9) (Cell Signaling #9336, 1:1000), Activin A (AnshLabs 18/16, A1006, 1:5000), Activin B (AnshLabs 12/9A, A1005, 1:2500), myostatin (Abcam ab3239, 1:1000) or GAPDH (Millipore #MAB374, 1:500 000) in blocking buffer, washed with TBS-0.05% Tween-20 and incubated with secondary antibody (goat-anti-rabbit Alexa Fluor 680; Molecular Probes or

goat-anti-mouse IRDye 800; Rockland Immunochemicals) 1 h at RT. Antibody binding was detected by Odyssey Infrared Imaging System (LI-COR) and quantified using the public domain NIH Image program (developed at the U.S. National Institutes of Health, Bethesda, MD).

Immunohistochemistry

After excision of the heart, a transversal section of the LV was fixed overnight in phosphate-buffered 10 % formaline (pH 7.0) and thereafter prepared as paraffin-embedded sections. Five µm thick sections were cut from the mid-section of the heart at the level of the papillary muscles. For staining, sections were deparaffinized in xylene and dehydrated in graded ethanol series. The sections were stained with Masson's trichrome stain to visualize cardiomyocytes, cell nuclei and fibrous tissue. Cross-sectional myocyte cell size was quantified (Nikon NIS-Elements) as an average of 50 cardiomyocytes/section obtained from five representative fields of each section (with 20× objective) from epicardial and endocardial sides of the LV. Apoptotic cells were determined by terminal deoxynucleotidyl transferase –mediated dUTP nick end labeling (TUNEL) method according to manufacturer's instructions (ApopTag in situ apoptosis detection kit, Chemicon). Sections were stained with anti-neutrophil (7/4, Abcam ab53453) with EnVision Detection System (Dako) for secondary detection to determine the number of infiltrated granulocytes. LV sections were stained with Activin A (AnshLabs 18/16, A1006), Activin B (AnshLabs 12/9A, A1005) and myostatin (Abcam ab3239) to determine the levels of ACVR2B ligands in heart after IR.

Doxorubicin treatment and analysis of mitochondrial function

The long-term effects of ACVR2B-Fc on cardiac function were analyzed in cardiotoxicity model. Male C57BL/6J mice aged 9-10 weeks were treated with vehicle, doxorubicin (Sigma, doxorubicin hydrochloride D1515) or doxorubicin and ACVR2B-Fc. Doxorubicin was injected i.p. four times every third day for 2 weeks (each injection 6 mg/kg) followed by two additional weeks to develop drug-induced cardiomyopathy. The doxorubicin administration protocol mimics the treatment of human patients with low doxorubicin doses, which induces cardiotoxicity. Mice were treated with ACVR2B-Fc or vehicle throughout 4-week study, administered as 5 mg/kg twice a week during the first 2 weeks and once a week after that. Heart mitochondrial function was measured using high-resolution respirometry (OROBOROS Oxygraph-2k) after 2 weeks of doxorubicin treatment. Five milligrams of cardiac LV tissue was homogenized with a shredder and analyzed using carbohydrate SUIT-protocol as described before.[10] FCCP was used at a concentration of 1 µM (titrated at 0.5, 1 and 1.5 µM). To estimate whether the changes in mitochondrial function may be at the level of Krebs cycle, mitochondrial citrate synthase (CS) activity was measured from the heart. The effect of cumulative doxorubicin toxicity was assessed with qPCR and with echocardiography, performed after 4 weeks of treatment under isoflurane anesthesia with Vevo 2100 ultrasound system.

Statistics

Data are expressed as mean±SD. The data were analyzed with SPSS software using Student *t* test, Mann–Whitney *U* test, or one-way ANOVA when appropriate, followed by Dunnett or Tukey post hoc test. *$p<0.05$, **$p<0.01$ and ***$p<0.001$.

Supplemental References

1. Gao, E., Lei, Y.H., Shang, X., Huang, Z.M., Zuo, L., Boucher, M. et al. (2010). A novel and efficient model of coronary artery ligation and myocardial infarction in the mouse. Circulation research *107*, 1445-1453.
2. Hulmi, J.J., Oliveira, B.M., Silvennoinen, M., Hoogaars, W.M., Ma, H., Pierre, P. et al. (2013). Muscle protein synthesis, mTORC1/MAPK/Hippo signaling, and capillary density are altered by blocking of myostatin and activins. Am. J. Physiol Endocrinol. Metab *304*, E41-E50.
3. Lee, S.J., Reed, L.A., Davies, M.V., Girgenrath, S., Goad, M.E., Tomkinson, K.N. et al. (2005). Regulation of muscle growth by multiple ligands signaling through activin type II receptors. Proc. Natl. Acad. Sci. U. S. A *102*, 18117-18122.
4. Kerkela, R., Karsikas, S., Szabo, Z., Serpi, R., Magga, J., Gao, E. et al. (2013). Activation of hypoxia response in endothelial cells contributes to ischemic cardioprotection. Molecular and cellular biology *33*, 3321-3329.
5. Kaikkonen, L., Magga, J., Ronkainen, V.P., Koivisto, E., Perjes, A., Chuprun, J.K. et al. (2014). p38alpha regulates SERCA2a function. Journal of molecular and cellular cardiology *67*, 86-93.
6. Dennler, S., Itoh, S., Vivien, D., ten Dijke, P., Huet, S., and Gauthier, J.M. (1998). Direct binding of Smad3 and Smad4 to critical TGF beta-inducible elements in the promoter of human plasminogen activator inhibitor-type 1 gene. The EMBO journal *17*, 3091-3100.
7. Korchynskyi, O., and ten Dijke, P. (2002). Identification and functional characterization of distinct critically important bone morphogenetic protein-specific response elements in the Id1 promoter. The Journal of biological chemistry *277*, 4883-4891.
8. Szabo, Z., Magga, J., Alakoski, T., Ulvila, J., Piuhola, J., Vainio, L. et al. (2014). Connective tissue growth factor inhibition attenuates left ventricular remodeling and dysfunction in pressure overload-induced heart failure. Hypertension *63*, 1235-1240.
9. Tolonen, A.M., Magga, J., Szabo, Z., Viitala, P., Gao, E., Moilanen, A.M. et al. (2014). Inhibition of Let-7 microRNA attenuates myocardial remodeling and improves cardiac function postinfarction in mice. Pharmacology research & perspectives *2*, e00056.
10. Rasanen, M., Degerman, J., Nissinen, T.A., Miinalainen, I., Kerkela, R., Siltanen, A. et al. (2016). VEGF-B gene therapy inhibits doxorubicin-induced cardiotoxicity by endothelial protection. Proceedings of the National Academy of Sciences of the United States of America *113*, 13144-13149.
